# Posttranscriptional regulation of PD-1 by PRMT5/WDR77 complex shapes T cell effector function and antitumor immunity

**DOI:** 10.1172/JCI191469

**Published:** 2026-02-02

**Authors:** Yinmin Gu, Yongbo Pan, Chang Pan, Qiang Pang, Zhantong Tang, Yiwen Chen, Haojing Zang, Xiaodong Wang, Chang Huang, Qingqing Zhang, Facai Yang, Xiaofeng Zhu, Yibi Zhang, Xujie Zhao, Shan Gao

**Affiliations:** 1 The Second Affiliated Hospital, Zhejiang University School of Medicine, Hangzhou, China.; 2 Zhongda Hospital, School of Life Sciences and Technology, Advanced Institute for Life and Health, Southeast University, Nanjing, China.; 3 Haiyuan College, Kunming Medical University, Kunming, China.; 4 School of Medicine, Advanced Institute for Life and Health, Southeast University, Nanjing, China.; 5 Department of Microbiology and Immunology, Shanxi Medical University, Taiyuan, China.; 6 Department of Immunology, School of Basic Medical Sciences, Hubei University of Medicine, Shiyan, China.; 7 Medical College, Guizhou University, Guiyang, China.; 8 Department of Gynecology, Women’s Hospital of Nanjing Medical University (Nanjing Women and Children’s Healthcare Hospital), Nanjing, China.

**Keywords:** Genetics, Immunology, Cancer, T cells, Transcriptomics

## Abstract

The regulation of the programmed cell death protein 1 (PD-1) gene, *PDCD1*, has been widely explored at transcription and posttranslational levels in T cell function and tumor immune evasion. However, the mechanism for *PDCD1* dysregulation at the posttranscriptional level remains largely unknown. Here, we identify protein arginine methyltransferase 5 (PRMT5) as a RNA binding protein in a methyltransferase activity–independent manner, which promotes *PDCD1* decay with WD repeat domain 77 protein (WDR77) and Argonaute2. Furthermore, the type-I IFN/STAT1 pathway transcriptionally activates *PRMT5* and *WDR77*, thus enhancing PRMT5/WDR77 binding on a conserved AU-rich element of *PDCD1* 3′ UTR. Functionally, conditional knockout of either *PRMT5* or *WDR77* in T cells disrupts T cell effector function and sensitizes the tumors to anti–PD-1 therapy. Clinically, *PRMT5* and *WDR77* expression in tumor-infiltrating T cells are negatively correlated with *PDCD1* expression and renders tumors resistant to PD-1–targeted immunotherapy. Moreover, fludarabine targeting STAT1 in combination with anti–PD-1 has a synergetic effect on suppressing tumor growth in mice. Overall, this study reveals that the RNA binding–dependent function of PRMT5 regulates *PDCD1* and T cell effector function with WDR77 and identifies potential combinatorial therapeutic strategies for enhancing antitumor efficacy.

## Abstract 2

This study reveals that PRMT5, in association with WDR77, regulates PDCD1 degradation, impacting T cell function and enhancing anti-PD-1 therapy efficacy.

## Introduction

Dysregulation of programmed cell death-1 (PD-1), encoded by the *PDCD1*, mediates T cell dysfunction and tumor immune evasion through engagement with its ligands, PD-L1 and PD-L2 (1, 2). Targeting PD-1/PD-L1 pathway shows some clinical benefits in various cancer types by reversing T cell dysfunction (3), but the limited efficacy highlights the need to study the mechanisms of *PDCD1* dysregulation. The regulation of *PDCD1* at the transcriptional and protein levels has been extensively studied (4–10); however, how *PDCD1* is regulated at RNA level and how this regulation influences antitumor immunity have not been fully elucidated.

RNA-binding proteins (RBPs) ubiquitously bind to RNAs and thereby regulate their life fates, including splicing, stability, localization, and translation, which is generally dependent on RNA binding domains (RBDs) (11). However, recent studies reveal that noncanonical RBPs without classical RBDs can also bind RNA to regulate their molecular fates (12–14), suggesting the pleiotropic potential of RNA regulation. Whether and how *PDCD1* is regulated by either RBPs or noncanonical RBPs remain largely unknown.

Protein arginine methyltransferase 5 (PRMT5) catalyzes the formation of symmetrical dimethylation on arginine residues of substrate proteins in a WD repeat domain 77 protein–dependent (WDR77-dependent) manner, which form a stoichiometric complex to regulate chromatin structure, gene transcription, and mRNA splicing (15, 16). Although PRMT5 also exerts methyltransferase activity–independent functions (17, 18), the underlying mechanisms remain unclear. In this study, we report that PRMT5, acting as a RBP, directly binds to the *PDCD1* 3′ UTR and promotes its mRNA degradation to maintain T cell effector function and antitumor immunity in a WDR77-dependent manner. Moreover, the type-I IFN/STAT1 pathway decreases PD-1 expression through STAT1-mediated transcriptional regulation of *PRMT5* and *WDR77*. In vivo, PRMT5 or WDR77 disruption in T cells enhances tumor response to anti–PD-1 immunotherapy. Furthermore, combined STAT1-targeted fludarabine and PD-1–targeted antibody effectively enhances the antitumor immunity. Thus, we uncover a mechanism for *PDCD1* dysregulation at an RNA level with potential cancer treatment.

## Results

### PRMT5 and WDR77 binds to the PDCD1 3′ UTR.

**Figure 1:**
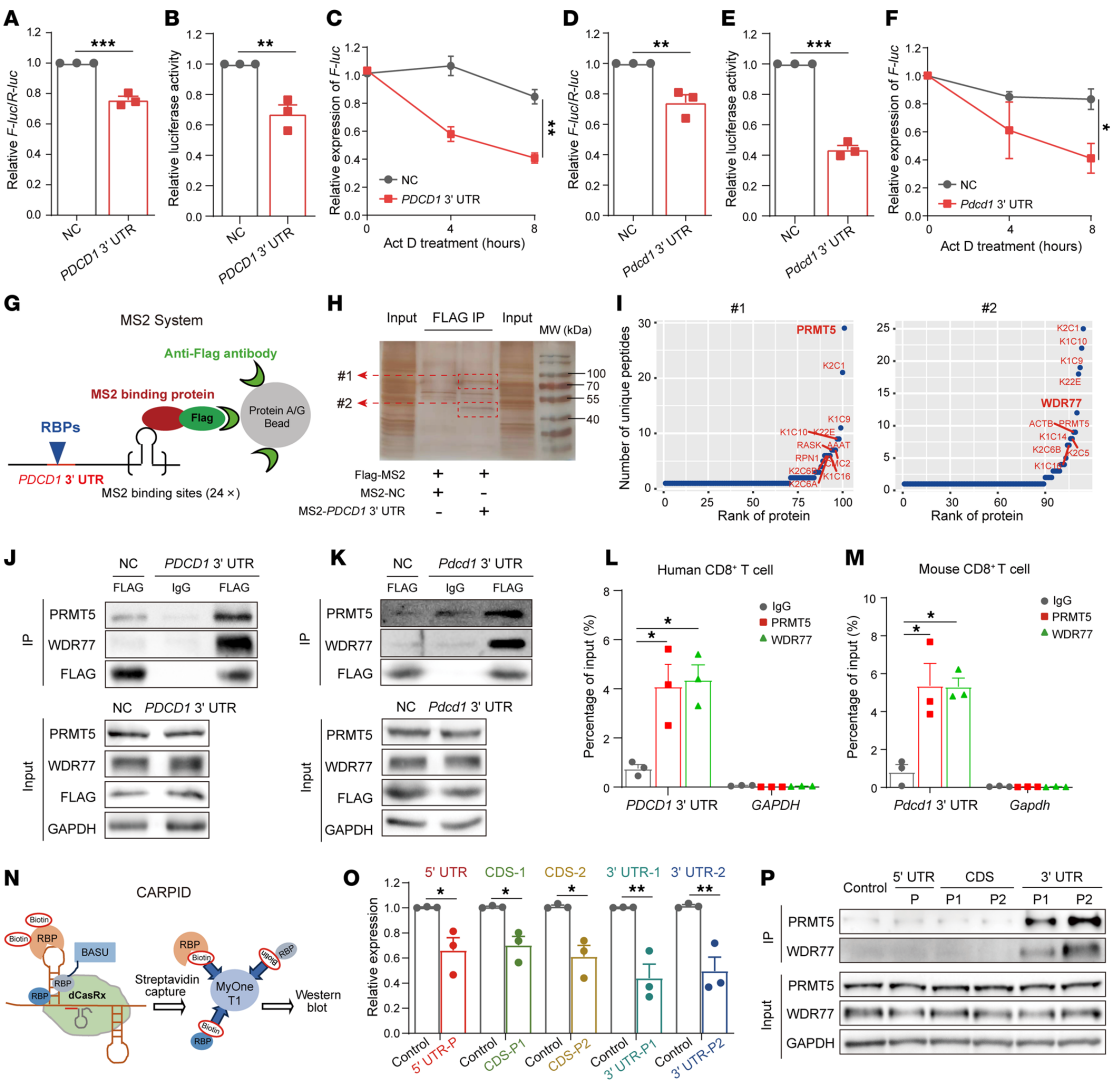
PRMT5 and WDR77 bind to the *PDCD1* 3′ UTR. (**A**–**F**) qPCR assays showing the relative mRNA abundance of *F-Luc* (**A** and **D**) or dual-luciferase assays showing the relative luciferase activities (**B** and **E**) and RNA stability assays showing the half lives of *F-Luc* (**C** and **F**) in HEK293T cells expressing *PDCD1* or *Pdcd1* 3′ UTR reporters. ActD, Actinomycin D; NC, negative control. (**G**) Schematic representation for MS2 aptamer-tagged *PDCD1* 3′ UTR/MS2-FLAG (MS2 system) RNA pull-down system. (**H**) Visualization of silver-stained protein bands from HEK293T cell lysates using MS2 aptamer-tagged *PDCD1* 3′ UTR. Red arrows for specific bands. MW, molecular weight. IP, immunoprecipitation. (**I**) Mass spectrometry showing the number of identified peptides and protein ranking for ~72 KDa (#1) and 40~55 KDa (#2) from **H**. (**J** and **K**) Immunoblotting analysis of the specific association of PRMT5 and WDR77 with MS2 aptamer-tagged *PDCD1* (**J**) or *Pdcd-1* (**K**) 3′ UTR in HEK293T cells. (**L** and **M**) RIP-qPCR analysis of *PDCD1* (**L**) or *Pdcd1* (**M**) 3′ UTR enriched by PRMT5 and WDR77 in human (**L**) and mouse (**M**) CD8^+^ T cells. (**N**) Schematic representation for the CRISPR-assisted RNA-protein interaction detection (CARPID) workflow. (**O**) qPCR assays showing the specificity of the CRISPR/CasRx system for *PDCD1*, normalized with *GADPH* (**P**). Immunoblotting analysis of PRMT5 and WDR77 in streptavidin IP samples of control and 5 XIST gRNA sets. For **A**–**F** and **O** (*n* = 3), by unpaired 2-tailed Student’s *t* test; For **L** and **M** (*n* = 3), by 1-way ANOVA with Dunnett’s test. Data are presented as mean ± SEM or SD. *P < 0.05, **P < 0.01, ***P < 0.001.

To investigate the potential regulatory role of the *PDCD1* 3′ UTR, we constructed a pmirGLO-*PDCD1* 3′ UTR firefly luciferase (F-luc) reporter, which showed that both RNA level and activity of F-luc were significantly reduced in the 3′ UTR reporter compared with the empty vector (Figure 1, A and B, and Supplemental Figure 1, A and B; supplemental material available online with this article; https://doi.org/10.1172/JCI191469DS1). After actinomycin D (Act D) treatment, the *PDCD1* 3′ UTR inhibited the mRNA stability of *F-luc* (Figure 1C and Supplemental Figure 1C). Consistently, mouse *Pdcd1* 3′ UTR also decreased the RNA level/stability and activity of F-luc (Figure 1, D–F), suggesting that in both human and mouse, *PDCD1* is negatively regulated by its 3′ UTR. Given that the 3′ UTRs of mRNA are generally bound and mediated by RBPs (19), we performed a MS2 pull-down assay (Figure 1G) followed by liquid chromatography tandem mass spectrometry (LC-MS/MS) to identify potential RBPs bound to the 3′ UTR of *PDCD1*. Notably, a known PRMT5/WDR77 complex was found to be most associated with the 3′ UTR of *PDCD1* (Figure 1, H and I). We performed a MS2 pull-down assay followed by immunoblotting assays, which showed that *PDCD1* 3′ UTR was binding with either PRMT5 or WDR77 (Figure 1, J and K, and Supplemental Figure 1D). Furthermore, RNA immunoprecipitation-quantitative real-time PCR (RIP-qPCR) assays revealed that the endogenous *PDCD1* 3′ UTR, but not the *GAPDH* control, were significantly enriched in either PRMT5 or WDR77 immunoprecipitation (IP) (Figure 1, L and M, and Supplemental Figure 1, E–G), thereby implying that PRMT5 and WDR77 physically associate with the *PDCD1* 3′ UTR. Consistently, the RPIseq program (20) showed that PRMT5 binds to the 3′ UTRs with high probability using 2 methods, whereas WDR77 positively interacts with the 3′ UTRs using RPISeq-RF method, but not RPISeq-SVM method (Supplemental Figure 1H). Subsequently, we applied CRISPR-assisted RNA-protein interaction detection (CARPID) method (21) to test whether the complex specifically binds to 3′ UTR region of *PDCD1* transcript (Figure 1N and Supplemental Figure 1I). Nuclease-active CasRx showed specific digestion of targeted regions by these gRNAs (Figure 1O). Immunoblotting confirmed the specific associations of PRMT5 and WDR77 with the 3′ UTR region of *PDCD1*, especially the *PDCD1* 3′ UTR tail fragment (Figure 1P). 3′ UTR had been reported to regulate protein complex formation (22), thus we examined whether the *PDCD1* 3′ UTR affect the interaction between PRMT5 and WDR77. Co-Immunoprecipitation (Co-IP) assays showed that neither *PDCD1* 3′ UTR knockdown (KD) in NCI-1299 cells expressing PD-1 (23) nor overexpression (OE) in 293T cells alter the association between PRMT5 and WDR77 (Supplemental Figure 1, J–O). Collectively, these results indicate that either PRMT5 or WDR77 binds to the endogenous *PDCD1* 3′ UTR of either human or mice.

### PRMT5/WDR77 complex decreases PDCD1 mRNA stability.

**Figure 2:**
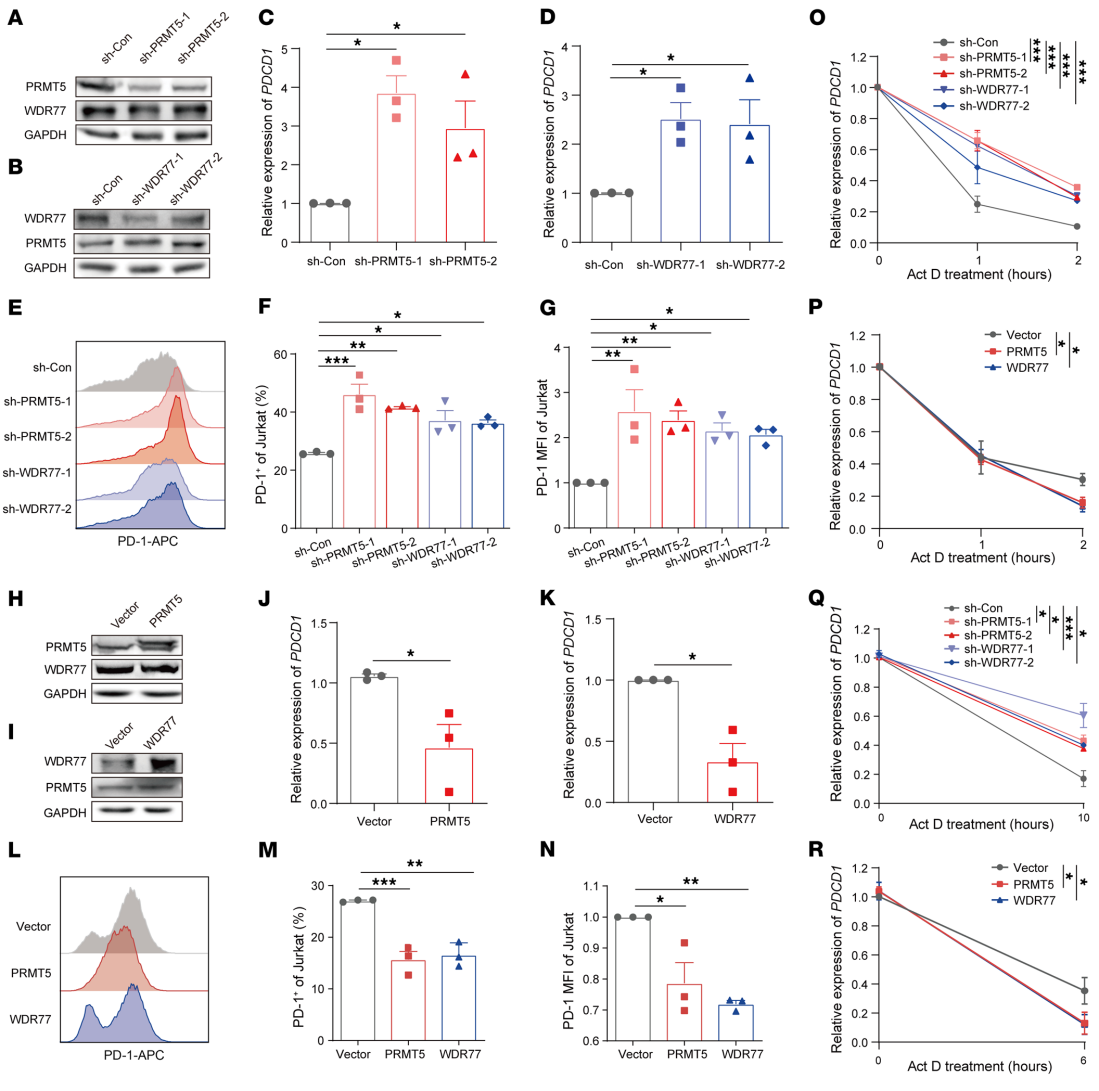
PRMT5 and WDR77 negatively regulate *PDCD1*. (**A** and **B**) Immunoblotting analysis of PRMT5 and WDR77 expression in PRMT5 (**A**) or WDR77 (**B**) KD Jurkat cells. (**C**–**G**) The relative mRNA levels of *PDCD1* (**C** and **D**), mean fluorescence intensity (MFI) plots (**E**), percentages of positive cells (**F**) and relative MFI levels (**G**) of PD-1 in PRMT5 or WDR77 KD Jurkat cells stimulated with phytohaemagglutinin (PHA). (**H** and **I**) Immunoblotting analysis of PRMT5 and WDR77 expression in PRMT5 (**H**) or WDR77 (**I**) OE Jurkat cells. (**J**–**N**) The relative mRNA levels of *PDCD1* (**J** and **K**), MFI plots (**L**), percentages of positive cells (**M**) and relative MFI levels (**N**) of PD-1 in PRMT5 or WDR77 OE Jurkat cells stimulated with PHA. (**O** and **P**) Half-lives of *PDCD1* in the indicated Jurkat cells treated with PHA. (**Q** and **R**) 4sU pulse labeling and pulse chase analysis of the RNA decay for *PDCD1* in the indicated Jurkat cells treated with PHA for 48 hours. For **C**–**G** and **M**–**R** (*n* = 3), by 1-way ANOVA with Dunnett’s test; for **J** and **K** (*n* = 3), by unpaired 2-tailed Student’s *t* test. Data are presented as mean ± SEM or SD. *P < 0.05, **P < 0.01, ***P < 0.001.

To determine the effects of PRMT5/WDR77 complex on *PDCD1* expression, we performed qPCR and fluorescence activated cell sorting (FACS) assays. Results showed that KD of either PRMT5 or WDR77 upregulated, whereas OE of either PRMT5 or WDR77 reduced the mRNA and cell surface expression of PD-1 (Figure 2, A–N, and Supplemental Figure 2, A–H). Consistently, heterozygous knockout (KO) of either PRMT5 or WDR77 increased the mRNA and cell surface expression of PD-1 (Supplemental Figure 3, A–H). These results suggested that PRMT5 and WDR77 suppress the mRNA and protein levels of PD-1. Given that our observation that *PDCD1* 3′ UTR inhibits the stability of *F-luc*, we examined whether PRMT5 and WDR77 affects the mRNA stability of *PDCD1*. RNA stability assays revealed that both KD and KO of either PRMT5 or WDR77 increased, while OE of either PRMT5 or WDR77 inhibited the mRNA stability of *PDCD1* (Figure 2, O and P, and Supplemental Figure 3I). Moreover, RNA decay analysis using nascent RNA in living cells labeled with 4-Thiouridine (4-SU) revealed that both KD and KO of either PRMT5 or WDR77 elevated, whereas OE of either PRMT5 or WDR77 suppressed, the abundance and stability of nascent *PDCD1* (Figure 2, Q and R, and Supplemental Figure 3, J–M). Altogether, these data indicate that PRMT5 and WDR77 negatively regulate *PDCD1* mRNA stability.

We next evaluated the relationship between PRMT5 and WDR77 in regulating the *PDCD1* expression. The rescue experiments showed that WDR77 OE did not abrogate the effects of PRMT5 silencing on the mRNA abundance/stability and cell surface expression of PD-1 (Supplemental Figure 4, A–D). Conversely, PRMT5 OE did not reverse the increased mRNA abundance/stability and cell surface expression of PD-1 induced by WDR77 KD (Supplemental Figure 4, E–H), suggesting that PRMT5 and WDR77 is interdependent for regulation of *PDCD1* expression. Moreover, the simultaneous KD of PRMT5 and WDR77 further enhanced both the mRNA abundance/stability and protein expression levels of PD-1 (Supplemental Figure 4, I–K). Together, these results demonstrate that PRMT5 and WDR77 may cooperate to decrease *PDCD1* expression. Since PD-1 expression is markedly upregulated upon T cell receptor (TCR) activation, maintaining its expression within an appropriate range is critical for preserving T cell homeostasis (9, 24). We therefore assessed whether TCR signals regulate PRMT5 and WDR77. Analysis of quiescent versus activated human and murine T cells revealed significant upregulation of both proteins following activation (Supplemental Figure 4, L and M), suggesting that TCR-induced increases in PRMT5 and WDR77 help maintain PD-1 expression within an optimal range in proficient T cells.

### PRMT5 is a RBP to regulate PDCD1 expression.

As an arginine methyltransferase, PRMT5 in complex with WDR77 regulates gene transcription and mRNA splicing and stability (16), which led us to examine whether PRMT5 regulates *PDCD1* expression dependent on its methyltransferase activity. We used 2 substrate-competitive PRMT5 inhibitors (25, 26) to assess the *PDCD1* expression. The expression levels of p53 as a positive control were increased by EPZ015666 or GSK3326595 in a dose-dependent manner (Supplemental Figure 5, A–D). However, mRNA and cell surface expressions of PD-1 were not affected by the 2 inhibitors (Supplemental Figure 5, E–T), supporting that PRMT5 regulates *PDCD1* expression independently of its methyltransferase activity. Furthermore, we performed LC-MS/MS to determine whether PRMT5 functions as an RNA methyltransferase, which showed that either PRMT5 or WDR77 KD had no significant effect on the levels of 29 common RNA modifications, including RNA methyl modifications (Supplemental Figure 5U), suggesting that PRMT5 has no methyltransferase activity on RNA.

**Figure 3:**
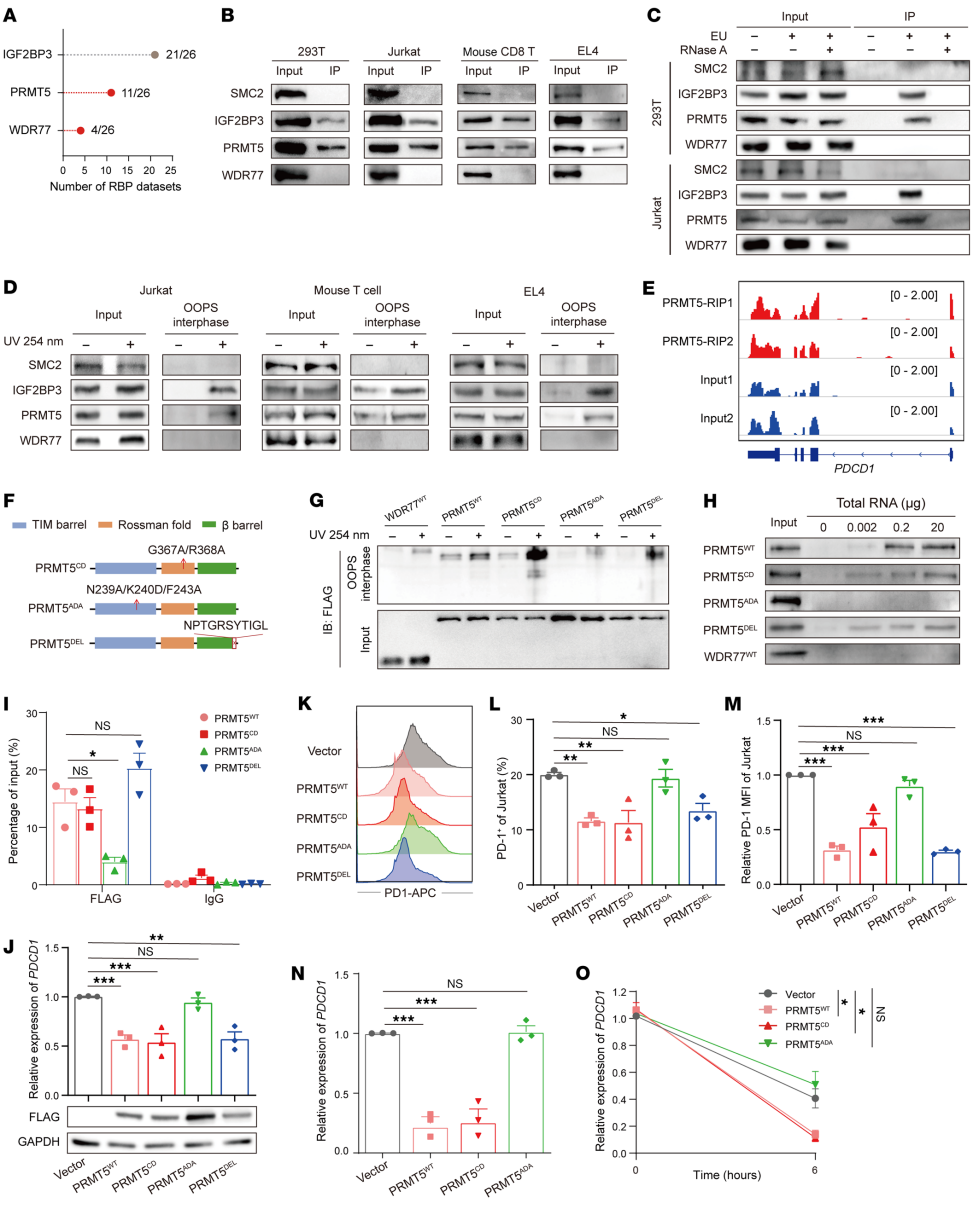
PRMT5 acts as a RBP to regulate *PDCD1* expression. (**A**) Published proteome-wide studies of RBPs showing the possibility of PRMT5 and WDR77 as RBP. (**B**) Immunoblotting analysis of PRMT5 and WDR77 binding to oligo(dT) beads in the indicated cells. SMC2 as a negative control and IGF2BP3 as a positive control. (**C** and **D**) Immunoblotting analysis for PRMT5 and WDR77 either binding to EU-labeled RNA (**C**) or in OOPS interphase (**D**) in the indicated cells. (**E**) RIP-seq showing the enrichment of PRMT5 in *PDCD1* mRNA. (**F**) Schematic representation for the mutants of PRMT5. (**G**) Immunoblotting of OOPS interphase for FLAG in HEK293T cells expressing the indicated constructs. (**H**) Immunoblotting for indicated proteins binding to oligo (dT) beads in varying amounts of total RNA. (**I**) RIP-qPCR analysis of *PDCD1* 3′ UTR enriched by FLAG in Jurkat cells expressing the indicated constructs. (**J**) The relative levels of *PDCD1* (top) and immunoblotting analysis for FLAG (bottom) in PHA-stimulated Jurkat cells expressing the indicated constructs. (**K**–**M**) Representative MFI plots (**K**), percentages of positive cells (**L**) and relative MFI levels (**M**) of PD-1 in PHA-stimulated Jurkat cells expressing the indicate. (**N** and **O**) 4sU pulse labeling and pulse-chase analysis of the nascent RNA levels (**N**) and RNA decay (**O**) of *PDCD1* in PHA-stimulated Jurkat cells expressing the indicated constructs. For **I**, **J**, and **L**–**O** (*n* = 3), by 1-way ANOVA with Dunnett’s test. Data are presented as mean ± SEM or SD. *P < 0.05, **P < 0.01, ***P < 0.001. NS, not significant.

Given that the binding of PRMT5 and WDR77 to the *PDCD1* 3′ UTR, we speculated PRMT5 and WDR77 as potential RBPs. The published proteome-wide studies of RBPs showed that PRMT5 and WDR77 were detected, respectively, in 11 and 4 independent datasets (Figure 3A and Supplemental Table 1), highlighting a possibility for PRMT5 and WDR77 as RBPs. To further validate this, we employed oligo (dT) beads to pull down poly (A) mRNAs and assessed the binding abilities of the complex to mRNAs. The immunoblotting assays showed that PRMT5, but not WDR77 bound to poly (A) RNAs (Figure 3B). In addition, capture of the newly transcribed RNA interactome using click chemistry (RICK) (27) followed by immunoblotting assays also showed that PRMT5, but not WDR77, interacted with EU-labeled RNAs, which was abrogated by RNase A treatment (Figure 3C), implying that PRMT5 is able to directly bind to RNA. To verify the direct binding of endogenous PRMT5 to RNA, we applied the orthogonal organic phase separation (OOPS) method (28), which revealed that UV crosslinking resulted in the partitioning of endogenous IGF2BP3 and PRMT5, but not WDR77 and SMC2, in the acidic guanidinium-thiocyanate-phenol-chloroform (AGPC) interphase (Figure 3D), further confirming that the binding ability of PRMT5 to RNA in intact cells. Moreover, 2 replicate samples of crosslinked RIP-sequencing (RIP-seq) identified 8,297 RNA targets that interact with PRMT5 (Supplemental Figure 6A and Supplemental Table 2), including *PDCD1* mRNA (Figure 3E). Through Kyoto Encyclopedia of Genes and Genomes (KEGG) pathway analysis, some of these targets were enriched in pathways of the immune system, including the “PD-L1 expression and PD-1 checkpoint pathway in cancer” and “T cell receptor signaling pathway” (Supplemental Figure 6B), suggesting that PRMT5 may act as an RBP to regulate immune processes. Together, these data support that PRMT5, but not WDR77, is a RBP in human and mouse.

We next identified which domain of PRMT5 is responsible for binding RNA. We first utilized RNABindRPlus (29) to predict the RNA-binding region (RBR) of PRMT5. Intriguingly, one of the key predicted regions is located in the C-terminal of PRMT5 (Supplemental Figure 6C). As expected, any notable RBR of WDR77 was not observed (Supplemental Figure 6C). Then, a deletion mutant (PRMT5^DEL^) of the predicted RBR for PRMT5 was constructed (Figure 3F). Additionally, we also constructed the other 2 mutants of PRMT5, including the catalytically dead (PRMT5^CD^: G367A, R368A) (30) and substrate recruitment motif mutants (PRMT5^ADA^: N239A, K240D, F243A) (15) (Figure 3F). The OOPS assays showed that PRMT5^WT^, PRMT5^CD^, and PRMT5^DEL^ were detected in the interphase, but not WDR77^WT^ and PRMT5^ADA^ (Figure 3G). Moreover, oligo (dT) pulldown assays revealed that the binding abilities of in vitro translated–PRMT5^WT^ (IVT-PRMT5^WT^), PRMT5^CD^, and PRMT5^DEL^ to RNAs were increased in a dose-dependent manner, which were not observed in IVT-WDR77^WT^ and PRMT5^ADA^ (Figure 3H), indicating that PRMT5 binds to RNA dependent on substrate recruitment motif. Moreover, *PDCD1* 3′ UTR did not affect the level of endogenous symmetric dimethylarginine (SDMA) and p53 expression (Supplemental Figure 6D), suggesting that the enrichment of RNA on the PRMT5 may not affect the PRMT5-dependent arginine methylation. These data support that the substrate recruitment motif is the RBD of PRMT5, which is necessary for its binding to RNA.

To further explore the regulation of these PRMT5 mutants on PD-1 expression, we firstly performed RIP-qPCR assays, which revealed that PRMT5^CD^ and PRMT5^DEL^ had comparable enrichment levels on either human or mouse *PDCD1* 3′ UTR with PRMT5^WT^, but the interactions between PRMT5^ADA^ and the 3′ UTRs were obviously impaired (Figure 3I and Supplemental Figure 6E). Furthermore, PRMT5^WT^, PRMT5^CD^ and PRMT5^DEL^ reduced the mRNA abundance and cell surface expression of PD-1, but these effects were significantly abolished in PRMT5^ADA^ OE cells (Figure 3, J–M, and Supplemental Figure 6, F and G). Moreover, PRMT5^WT^ and PRMT5^CD^, but not PRMT5^ADA^, reduced the abundance and stability of nascent *PDCD1* (Figure 3, N–O). All together, these data suggest that PRMT5 functions as an RBP to regulate *PDCD1* expression at RNA level.

The argonaute (AGO) proteins, AGO1–4, are highly conserved ribonuclease proteins involved in RNA silencing pathways (31). In *Arabidopsis*, AGO2 interacts with PRMT5 (32), prompting us to explore whether PRMT5 regulates *PDCD1* RNA stability through AGO2. Co-IP assays confirmed that PRMT5 associated with AGO2 in T cells (Supplemental Figure 7, A–D), while KD of PRMT5 or AGO2 did not affect each other’s expression (Supplemental Figure 7, E and F). RIP-qPCR assays showed that *PDCD1* 3′ UTR was enriched by AGO2 that markedly reduced upon PRMT5 KD and enhanced upon PRMT5 OE (Supplemental Figure 7, G and H). Functionally, AGO2 KD mirrored PRMT5 KD, increasing PD-1 mRNA abundance, stability, and surface expression (Supplemental Figure 7, I–L), whereas AGO2 OE partially rescued the PD-1 upregulation caused by PRMT5 KD (Supplemental Figure 7, M–P). Collectively, these findings suggest that PRMT5 promotes *PDCD1* mRNA degradation via AGO2.

### PRMT5 regulates PDCD1 mRNA level through an AU-Rich element of 3′UTR.

To further determine the exact *PDCD1* 3′ UTR region responsible for binding the PRMT5/WDR77 complex and inhibiting *PDCD1* expression, we firstly constructed 4 luciferase reporters containing different *PDCD1* 3′ UTR fragments and found that all of these fragments can reduce the F-luc activity (Supplemental Figure 8, A and B), suggesting that any regions of the *PDCD1* 3′ UTR led to reduced inhibitory activity, which was also observed in a previous report (33). Furthermore, MS2 pull-down assays showed the 3′ UTR #4 and full-length (FL) fragments associated with the PRMT5 and WDR77 (Supplemental Figure 8C), suggesting that the 3′ UTR#4 fragment contains potential regulatory elements for binding the PRMT5/WDR77 complex. We further performed a conservative analysis for the *PDCD1* 3′ UTR among mammalian species, which revealed that a well conserved AU-rich element (ARE) across multiple species is defined as conserved ARE (cARE) (Supplemental Figure 8D). Additionally, we noted another ARE termed as non-cARE in human *PDCD1* 3′ UTR, but not in mouse (Supplemental Figure 8E). AREs are widely known as RBP binding sites and control mRNA stability (34), thus, we speculated that these AREs mediate the effect of the PRMT5/WDR77 complex on *PDCD1* expression. To validate this, the point or deletion mutant of 2 AREs for *PDCD1* 3′ UTR were constructed. RIP-qPCR and MS2 pull-down system assays showed that mutated cARE (M-cARE) and deletion (ΔARE) mutants, but not the M-non-cARE, decreased the enrichment of PRMT5/WDR77 complex in the *PDCD1* 3′ UTR (Supplemental Figure 8, F and G). Furthermore, luciferase reporter assays showed that either PRMT5 or WDR77 KD increased F-luc activities of the WT and M-non-cARE reporter, but not of the M-cARE reporter (Supplemental Figure 8H). Similar to the observations of *PDCD1* 3′ UTR, both M-ARE and ΔARE mutants of the mouse *Pdcd1* 3′ UTR restrained the association of PRMT5/WDR77 complex and the 3′ UTR (Supplemental Figure 8, I and J). All together, these results reveal that the cARE is critical for *PDCD1* mRNA decay in a PRMT5/WDR77-dependent manner.

To further investigate the interaction between PRMT5 and WDR77 in association with *PDCD1* 3′ UTR, we performed RIP-qPCR assays, which showed that either PRMT5 or WDR77 KD reduced, whereas either PRMT5 or WDR77 OE increased their enrichment on *PDCD1* 3′ UTR (Supplemental Figure 9, A–D). However, PRMT5^ADA^ did not affect the association of WDR77 with *PDCD1* 3′ UTR (Supplemental Figure 9D). Next, we performed electrophoretic mobility shift assay (EMSA), which showed that either *PDCD1* or *Pdcd1* 3′ UTR probes bound to ITV PRMT5^WT^, PRMT5^CD^, and PRMT5^DEL^, but not ITV WDR77^WT^ and PRMT5^ADA^ (Supplemental Figure 8, E and F), whereas the mutate probes exhibited poor affinity with ITV PRMT5^WT^ (Supplemental Figure 8, G and H). Notably, cold probe decreased the binding of ITV PRMT5^WT^ with cARE (Supplemental Figure 8, G and H), further confirming the specificity of the interaction of PRMT5 with cARE. To confirm that PRMT5 regulates *PDCD1* mRNA in an RBP-dependent manner, we used a tethered reporter (Supplemental Figure 8I) and found that PRMT5^WT^ that was fused to the λΝ peptide reduced F-luc activities of reporters containing the WT *PDCD1* or *Pdcd1* 3′ UTR, but not PRMT5^ADA^ fused to the λΝ peptide (Supplemental Figure 8, J and K). M-cARE also ablated the inhibitory effects of PRMT5^WT^ on either *PDCD1* or *Pdcd1* 3′ UTRs (Supplemental Figure 8, J and K). Overall, these data indicate that PRMT5 restrains *PDCD1* expression by directly binding to the cARE of the *PDCD1* 3′ UTR in a WDR77-dependent manner.

### PRMT5/WDR77 maintains peripheral T cell homeostasis independently of the PD-1 pathway.

To further explore the role of PRMT5 and WDR77 in regulating T cell function and antitumor immunity in vivo, we generated their specific deletion in T cells of conditional knockout (*Prmt5^CKO^* or *Wdr77^CKO^*) and knockdown (*Prmt5^CKD^* or *Wdr77^CKD^*) mice through crossing their flox mice with *CD4^cre^* mice (Supplemental Figure 10, A–D). Immunoblotting assays confirmed that PRMT5 or WDR77 proteins were deleted or reduced in T cells from spleen of these mice (Supplemental Figure 10, E–H). Naive T cells of the both mice had normal cell surface levels of T cell receptor (TCR) and CD28 (Supplemental Figure 10, I and J). Subsequently, we analyzed the T cell populations from the thymus and periphery of *Prmt5^CKO^* and *Wdr77^CKO^* mice. T cell development in the thymus of *Prmt5^CKO^* and *Wdr77^CKO^* mice did not differ markedly from their respective WT littermate controls (Supplemental Figure 10, K–N). However, compared with WT mice in spleen and lymph nodes, both *Prmt5^CKO^* and *Wdr77^CKO^* mice showed decreased numbers and frequencies of CD3^+^ and CD8^+^ T cells, and reduced numbers of CD4^+^ T cells, which accompanied by impaired frequencies of Ki-67, except for the spleen of *Wdr77^CKO^* mice (Supplemental Figure 11, A–L), indicating that the peripheral T cell homeostasis from *Prmt5^CKO^* and *Wdr77^CKO^* mice was dysregulated, which may be due to the defects in T cell expansion. Most notably, the cell surface expression of PD-1 was upregulated in the CD8^+^ T cells of both *Prmt5^CKO^* and *Wdr77^CKO^* mice (Supplemental Figure 11, B, E, H, and K), which led us to test whether the deficiency of PRMT5 or WDR77 disrupt the peripheral T cell homeostasis through the PD-1 pathway. Administration of anti–PD-1 antibody failed to rescue the numbers and frequencies of T cells from the spleen and lymph nodes of both *Prmt5^CKO^* and *Wdr77^CKO^* mice (Supplemental Figure 12, A–J), suggesting that PRMT5 and WDR77 regulate the peripheral T cell homeostasis independently of the PD-1 pathway.

### PRMT5/WDR77 maintains T cell effector function and antitumor immunity through PD-1.

**Figure 4:**
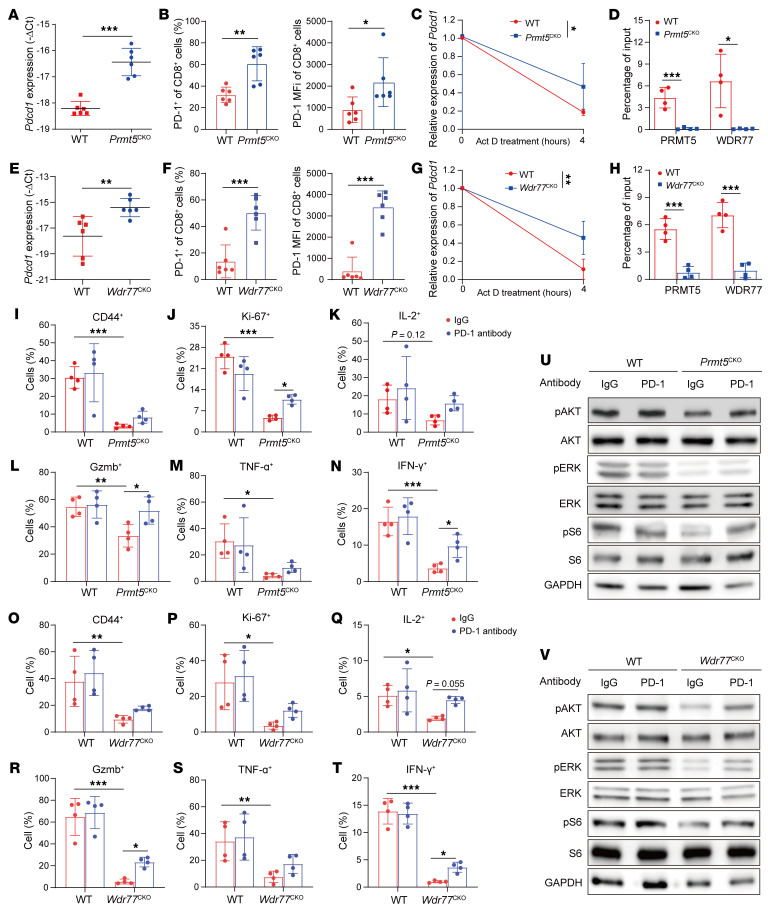
PRMT5/WDR77 maintain CD8^+^ T cell effector function through PD-1. (**A**–**H**) qPCR assays showing mRNA levels of *Pdcd1* (**A** and **E**) or FACS assays showing percentages of positive cells (left) and MFI levels of PD-1 (right) (**B** and **F**) or RNA stability assays showing half-lives of *Pdcd1* (**C** and **G**) and RIP-qPCR analysis of *Pdcd1* 3′ UTR enriched by PRMT5 and WDR77 (**D** and **H**) in the anti-CD3/28–stimulated CD8^+^ T cells from *Prmt5^CKO^* or *Wdr77^CKO^* and their WT mice. Delta threshold cycle (ΔC_t_) normalized to *18S*. (**I**–**T**) Effector molecule production in activated CD8^+^ T cells treated with anti–PD-1 antibody from *Prmt5^CKO^* (**I**–**N**) or *Wdr77^CKO^* (**O**–**T**) and their WT mice measured by CD44, Ki-67, IL-2, Gzmb, TNF-α, and IFN-γ. (**U** and **V**) Immunoblotting for phosphorylated AKT, ERK, and S6 in activated CD8^+^ T cells treated with anti–PD-1 antibody from *Prmt5^CKO^* (**U**) or *Wdr77^CKO^* (**V**) and their WT mice. For **A**, **B**, and **E**–**G** (*n* = 6), for **C** (*n* = 5), for **D** and **H** (*n* = 4), by unpaired 2-tailed Student’s *t* test; for **I**–**T** (*n* = 4), by 2-way ANOVA. Data are presented as mean ± SEM or SD. *P < 0.05, **P < 0.01, ***P < 0.001.

Similar to the Jurkat cells, in activated CD8^+^ or CD4^+^ mouse T cells, the deficiency of PRMT5 or WDR77 increased the mRNA abundance/stability and cell surface expression of PD-1 and almost completely abolished the enrichment of them with the *Pdcd1* 3′ UTR (Figure 4, A–H, and Supplemental Figure 13, A–D), further supporting that either PRMT5 or WDR77 led to reduced expression of PD-1 through binding the *Pdcd1* 3′ UTR. Given that PD-1 has a key role in T cell functions (2), we next tested whether the deficiency of PRMT5 or WDR77 impairs T cell function. FACS assays showed that ex vivo stimulation of CD8^+^ T cells purified from *Prmt5^CKO^* and *Wdr77^CKO^* mice elevated cell death and decreased amounts of CD44, Ki-67, granzyme B (Gzmb), and cytokines, which showed a trend toward partial restoration upon PD-1 blockade (Figure 4, I–T, and Supplemental Figure 13, E–G). The similar results of CD4^+^ T cell effector function were also observed in *Prmt5^CKO^* mice (Supplemental Figure 13, H–M). However, the expression of exhaustion-associated molecules, T cell immunoglobulin and mucin domain–containing protein 3 (TIM-3), and lymphocyte-activation gene 3 (LAG-3) were comparable in activated CD8^+^ T cells from *Prmt5^CKO^* or *Wdr77^CKO^* and control mice (Supplemental Figure 13, N–Q). These results indicate that PRMT5 and WDR77 maintain T cell effector function partially through PD-1 signaling. PD-1 ligation impairs the activity of 2 signaling cascades, the PI3K/AKT and the MAPK/ERK pathways (23, 35), so we explored the effects of PRMT5 or WDR77 deficiency on the 2 signaling pathways. Indeed, phosphorylated levels of AKT, ERK, and S6 were dramatically decreased in activated CD8^+^ T cells from *Prmt5^CKO^* or *Wdr77^CKO^* mice compared with control T cells, which were partially reversed by anti–PD-1 antibody (Figure 4, U and V). Altogether, these data demonstrate that PD-1 is indispensable for PRMT5 and WDR77 in sustaining T cell effector function.

**Figure 5:**
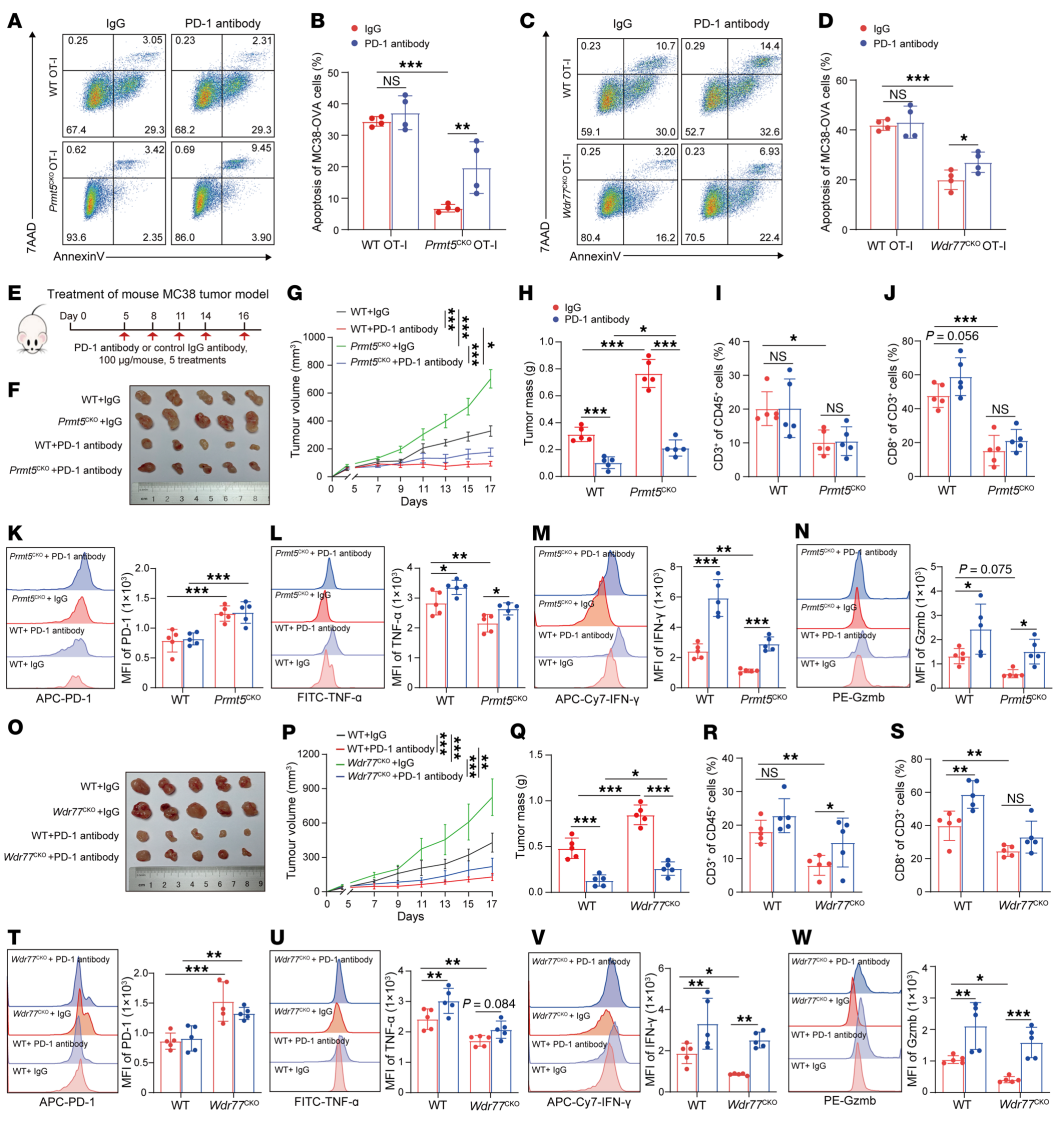
PRMT5/WDR77 maintain antitumor immunity through PD-1. (**A**–**D**) Representative flow cytometry plots (**A** and **C**) and quantification (**B** and **D**) of in vitro killing of MC38-OVA cells by CD8^+^ T cells from *Prmt5^CKO^* or *Wdr77^CKO^* OT-1 mice with or without anti–PD-1 treatment. (**E**) A schematic treatment plan for *Prmt5^CKO^* or *Wdr77^CKO^* and their WT mice bearing subcutaneous MC38 tumors. (**F**–**W**) Endpoint tumor size (**F** and **O**), tumor growth curves (**G** and **P**), and tumor mass (**H** and **Q**) of MC38 tumors in *Prmt5^CKO^* or *Wdr77^CKO^* and their WT mice treated with IgG or anti–PD-1 antibody. Flow cytometry analysis showing the percentage of CD3^+^ (**I** and **R**) /CD8^+^ (**J** and **S**) and the MFI of PD-1 (**K** and **T**) /TNF-α (**L** and **U**) /IFN-γ (**M** and **V**) /Gzmb (**N** and **W**) in the indicated cell populations of subcutaneous MC38 tumors from *Prmt5^CKO^* or *Wdr77^CKO^* and their WT mice with indicated treatments. For **B** and **D** (*n* =4), for **G**–**N** and **P**–**W** (*n* = 5), by 2-way ANOVA. Data are presented as mean ± SEM or SD. *P < 0.05, **P < 0.01, ***P < 0.001.

To examine whether PRMT5 and WDR77 affects the cytotoxic activity of CD8^+^ T cells, we performed in vitro T cell killing assays. Compared with the control group, apoptosis of MC38 cells expressing ovalbumin (OVA) was significantly decreased in the group of coculture with either PRMT5 or WDR77-deficient OT-1 CD8^+^ T cells, which could be partially rescued by anti–PD-1 antibodies (Figure 5, A–D), suggesting that PRMT5 and WDR77 maintain the cytotoxic activity of CD8^+^ T cells through PD-1. We next investigated the importance of PRMT5 and WDR77 deficiency in anti-tumor immunity in vivo. We used mouse B16F10 melanoma or MC38 colorectal carcinoma models and found that both *Prmt5^CKO^* and *Wdr77^CKO^* mice showed faster tumor progression and shorter survival rates than WT mice (Supplemental Figure 14, A–C). Subsequently, we treated these mice with anti–PD-1 or control IgG antibodies, and found that anti–PD-1 treatment produces a larger reduction in tumor growth in *Prmt5^CKO^* or *Wdr77^CKO^* mice than in their respective WT littermate controls, suggesting that PRMT5 or WDR77 deficiency in T cells sensitize tumors to PD-1 blockade (Figure 5, E–H and O–Q). Moreover, tumors from either *Prmt5^CKO^* or *Wdr77^CKO^* mice showed a concomitant decrease in both CD3^+^ T cell and CD8^+^ T cell populations, the increased expression of PD-1, and the reduced expression of cytokines in tumor-infiltrating (TIL) CD8^+^ T cells (Figure 5, I–N and R–W). Anti–PD-1 therapy partially rescued expression of cytokines, but not the CD3^+^ and CD8^+^ population (Figure 5, I–N and R–W), which may account for the slightly larger tumor size observed in *Prmt5^CKO^* or *Wdr77^CKO^* mice compared with WT mice after anti–PD-1 treatment. Collectively, these results suggest that PRMT5 and WDR77 partially maintain antitumor immunity through PD-1.

### Type I IFN/STAT1 signaling mediates PD-1 downregulation through transcriptional activation of PRMT5 and WDR77.

**Figure 6:**
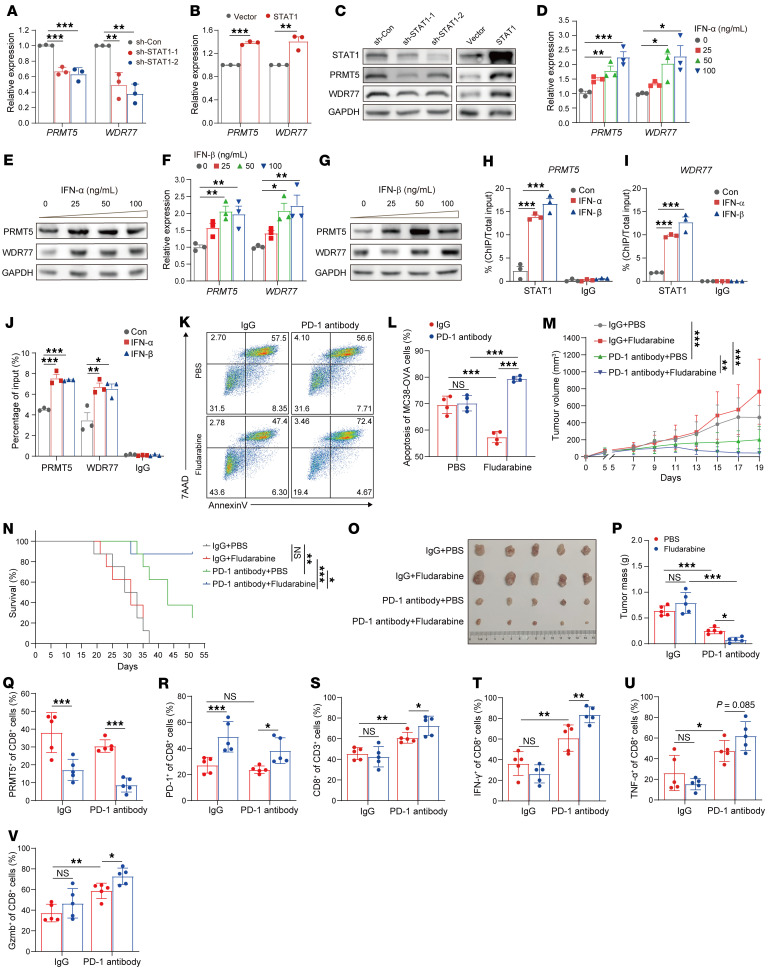
The type I IFN/STAT1 pathway decreases PD-1 expression through activation of PRMT5 and WDR77 transcriptionally. (**A** and **B**) The relative mRNA levels of *PRMT5* and *WDR77* in STAT1 KD (**A**) and OE (**B**) Jurkat cells. (**C**) Immunoblotting of PRMT5 and WDR77 expression in STAT1 KD (left) and OE (right) Jurkat cells. (**D**–**G**) The relative mRNA levels (**D** and **F**) and protein (**E** and **G**) levels of PRMT5 and WDR77 in Jurkat cells treated with IFN-α or IFN-β. (**H** and **I**) ChIP-qPCR analysis of *PRMT5* (**H**) or *WDR77* (**I**) promoter enriched by STAT1 in Jurkat cells treated with IFN-α or IFN-β. (**J**) RIP-qPCR analysis of *PDCD1 3*′ *UTR* enriched by PRMT5 or WDR77 in Jurkat cells treated with IFN-α or IFN-β. (**K** and **L**) Representative flow cytometry plots (**K**) and quantification (**L**) of in vitro killing of MC38-OVA cells by OT-1 CD8^+^ T cells with or without fludarabine or anti–PD-1 treatment. (**M** and **N**) Tumor growth curves (**M**) and survival rates (**N**) were assessed in mice treated with fludarabine and anti–PD-1. (**O**–**V**) Endpoint tumor size (**O**), tumor mass (**P**), and flow cytometry analysis (**Q**–**V**) showing the percentage of CD8^+^ T cells in CD3^+^ T cells (**S**), the percentage of PRMT5^+^ (**Q**), PD-1^+^ (**R**), IFN-γ^+^ (**T**), TNF-α^+^ (**U**),and Gzmb^+^ (**V**) in CD8^+^ T cell populations of subcutaneous MC38 tumors from mice treated with fludarabine and anti–PD-1. For **A**, **D**, **F**, and **H**–**J** (*n* = 3), by 1-way ANOVA with Dunnett’s test; for **B** (*n* = 3), by unpaired 2-tailed Student’s *t* test; For **L** (*n* = 4,) **M** (*n* = 8), and **P**–**V** (*n* = 5), by 2-way ANOVA. Data are presented as mean ± SEM or SD. *P < 0.05, **P < 0.01, ***P < 0.001.

To study upstream signals regulating expression of PRMT5/WDR77 in T cells, we predicted the common transcription factors of PRMT5 and WDR77 using software (36–38) and identified STAT1 as a candidate for its known function in T cells (39, 40) (Supplemental Figure 15A). We further analyzed public datasets (41) and found that both *PRMT5* and *WDR77* were positively correlated with the related genes of the IFN-JAK/STAT1 signaling pathway (Supplemental Figure 15B). Moreover, multiple ChIP-sequence datasets revealed the presence of STAT1 on either *PRMT5* or *WDR77* promoter in both mouse and human (Supplemental Figure 15, C–G), implying that STAT1 is an evolutionarily conserved transcription factor for the PRMT5 and WDR77. We next respectively constructed luciferase reporters with either *PRMT5* or *WDR77* promoter and found that STAT1 OE increased the F-luc activities of either *PRMT5* or *WDR77* promoter reporter (Supplemental Figure 16A). qPCR and immunoblotting assays showed that STAT1 KD decreased, whereas STAT1 OE increased, the mRNA and protein levels of PRMT5 and WDR77 (Figure 6, A–C and Supplemental Figure 16, B–D). Furthermore, STAT1 KD upregulated, while STAT1 OE downregulated, the cell surface levels of PD-1 expression (Supplemental Figure 16, E and F). These data demonstrated that STAT1 transcriptionally activates *PRMT5* and *WDR77*, which subsequently results in reduced expression of PD-1. Both type I (mainly IFN-α and IFN-β) and type II (IFN-γ) IFNs induce the formation of STAT1/STAT1 homodimers that translocate to the nucleus, thereby activating the transcription of genes (42, 43). Thus, we next investigated which IFNs mediated the effects of STAT1 on PRMT5 and WDR77 and found that IFN-α and IFN-β, but not IFN-γ, increased the mRNA and protein levels of PRMT5 and WDR77 (Figure 6, D–G, and Supplemental Figure 16, G–R), suggesting that type I IFN is involved in the regulation of STAT1 on PRMT5 and WDR77. Furthermore, both IFN-α and IFN-β treatments resulted in increased enrichment of STAT1 on either *PRMT5* or *WDR77* promoter (Figure 6, H and I), and led to elevated enrichment of PRMT5 and WDR77 on the *PDCD1* 3′ UTR (Figure 6J and Supplemental Figure 16S). Moreover, either IFN-α or IFN-β partially reduced the enhanced expression of PD-1 induced by either PRMT5 or WDR77 insufficiency (Supplemental Figure 16, T and U). Taken together, these findings indicate that the type I IFN/STAT1 pathway increases the expression of PRMT5 and WDR77, thereby suppressing PD-1 expression.

Fludarabine, a STAT1 inhibitor, is effective in the treatment of patients with advanced chronic lymphocytic leukemia (44), thus, we wondered whether fludarabine treatment enhances sensitivity to anti–PD-1 therapy through promoting PD-1 expression. Indeed, fludarabine treatment reduced the expression of STAT1, PRMT5, and WDR77, while increasing PD-1 expression (Supplemental Figure 17, A and B). When combined with anti–PD-1, fludarabine exerted a markedly synergistic effect on enhancement of the cytotoxic activity of CD8^+^ T cells in vitro (Figure 6, K and L, and Supplemental Figure 17C), suppressing tumor growth and improving survival rates of tumor-bearing mice, compared with anti–PD-1 treatment (Figure 6, M–P). Remarkably, tumors from 4 mice disappeared after 28 days of the combination therapy. FACS analysis of TILs further revealed that fludarabine decreased the proportion of PRMT5^+^ cells while increasing PD-1^+^ cells within the CD8^+^ T cell population (Figure 6, Q and R). The combination therapy further prompted CD8^+^ T cell infiltration and enhanced their cytokine production (Figure 6, S–V). Consistent with previous reports (10, 45, 46), either fludarabine or anti–PD-1 reduced the regulatory T cell (Tregs) percentages (Supplemental Figure 17D). Compared with anti–PD-1 treatment alone, the combination therapy did not significantly alter the frequencies of Tregs, monocytic (M) Myeloid-Derived Suppressor Cells (MDSCs), polymorphonuclear (P) MDSCs, myeloid dendritic cells (mDCs), conventional type 1 DCs (cDC1s), or plasmacytoid DCs (pDCs), tumor-associated macrophages (TAMs) (Supplemental Figure 17, E–J). Importantly, no significant changes in blood pressure, routine blood parameters, or liver function (Supplemental Figure 17, K–W), indicating that fludarabine enhances anti–PD-1 efficacy primarily through suppression of PRMT5, leading to PD-1 upregulation and reinforcement of antitumor immunity. Collectively, these findings highlight a critical role of the type I IFN/STAT1–PRMT5/WDR77–PD-1 axis in regulating antitumor immune responses.

### PRMT5 and WDR77 in T cells are associated with PDCD1 and the outcomes of anti–PD-1 therapy.

**Figure 7:**
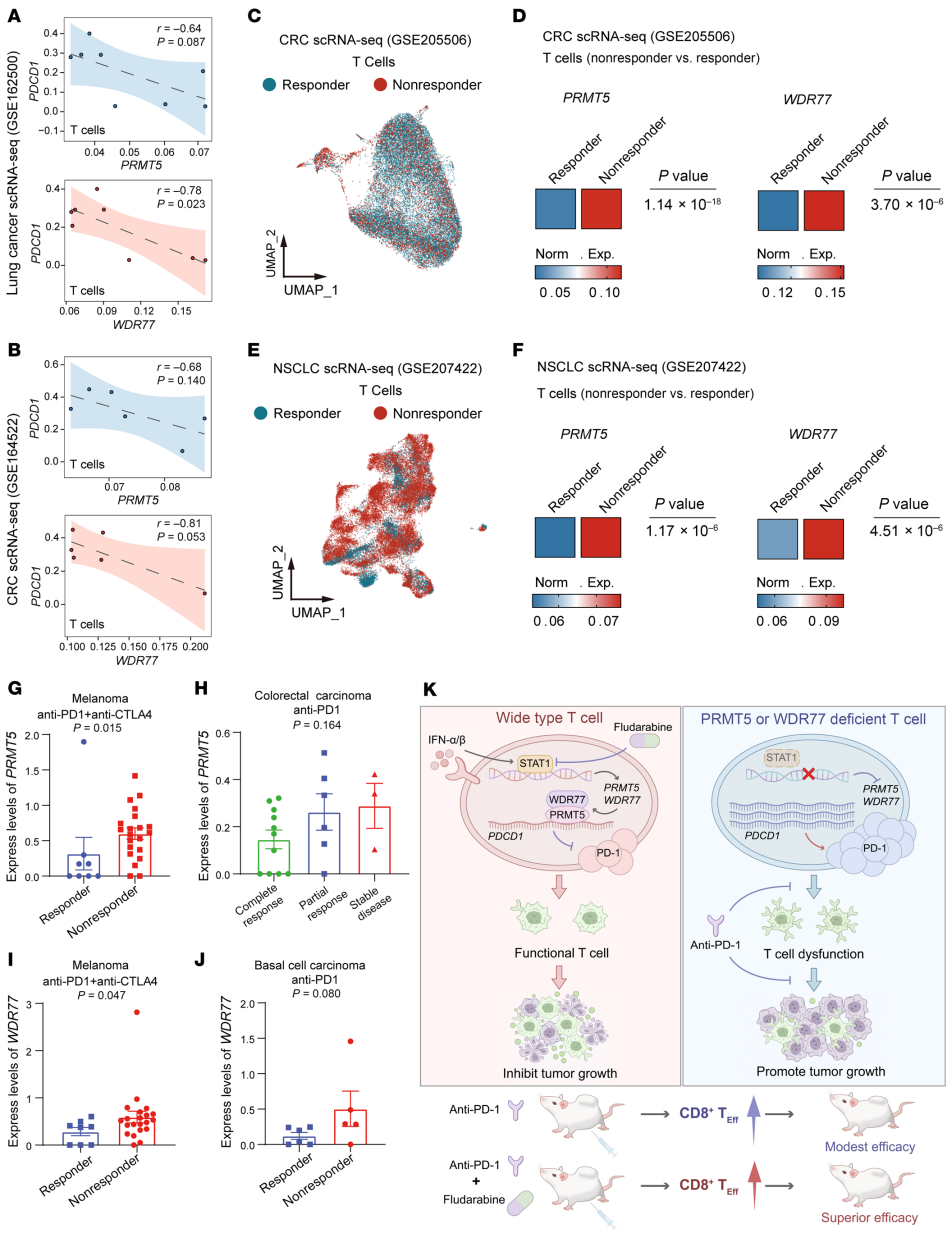
*PRMT5* and *WDR77* in T cells are associated with *PDCD1* and the outcomes of anti–PD-1 therapy. (**A** and **B**) Correlation analysis between *PRMT5* (top) or *WDR77* (down) expression and *PDCD1* in total T cells within TILs with lung cancer (**A**) and colorectal cancer (CRC) (**B**). (**C**–**F**) UMAP plots (**C** and **E**) of T cells from single-cell immunotherapy datasets showing populations from patients with differential responses to PD-1 blockade. Heatmaps (**D** and **F**) showing the normalized expression levels of *PRMT5* and *WDR77* in responder versus nonresponder patient groups based on PD-1 blockade response. (**G**–**J**) Expression levels of *PRMT5* and *WDR77* in patients with differential responses to PD-1 blockade in melanoma (**G** and **I**) or colorectal carcinoma (**H**) or basal cell carcinoma (**J**). (**K**) Proposed model for the type I IFN/STAT1 pathway transcriptionally activates *PRMT5* and *WDR77*, leading to decreased PD-1 expression and improving antitumor immunity (left). In vivo, disruption of PRMT5 or WDR77 in T cells enhances tumor response to anti–PD-1 immunotherapy (right). For **A** and **B**, by Pearson correlation analysis; for **G**, **I**, and **J**, by Mann-Whitney test; for **H**, by Kruskal-Wallis test. Data are presented as mean ± SEM or SD.

To further explore the significance of the PRMT5/WDR77-PD-1 axis in patients with cancer, we first investigated the correlation of expression between PRMT5 /WDR77 and PD-1. Analysis using TISIDB (47) and TIGER (48) softwares revealed a negative correlation between *PRMT5*/*WDR77* and *PDCD1* expression, as well as T cell dysfunction across multiple tumor types in the TCGA dataset (Supplemental Figure 18A), implying a potential immunomodulatory role for PRMT5/WDR77 in the tumor microenvironment. Single-cell RNA-seq (scRNA-seq) data from patients with lung and colorectal cancer (49, 50) further confirmed that *PRMT5* and *WDR77* expression were negatively correlated with *PDCD1* in total and CD8^+^ T cells within TILs (Figure 7, A and B, and Supplemental Figure 18, B and C). Consistently, the 10K Immunomes dataset (51) revealed a negative correlation between *PRMT5* or *WDR77* and *PDCD1* expression across multiple T cell subsets (Supplemental Figure 18, D and E). These data further support that PRMT5/WDR77 indeed inhibit the PD-1 expression in T cell.

We further analyzed the association between *PRMT5*/*WDR77* levels in T cells and response to anti–PD-1 therapy. In 2 cohorts of colorectal cancer and non–small cell lung cancer treated with anti–PD-1 (52, 53), T cells from responders exhibited significantly lower *PRMT5* and *WDR77* expression compared with nonresponders (Figure 7, C–F). Upon further examination of CD8^+^ T cell transcriptomic dataset (54) from patients with varying anti–PD-1 responses, we observed a significantly higher *PRMT5* and *WDR77* expression in the nonresponding group compared with responders (Figure 7, G–J). Collectively, these results suggest that an inverse correlation between *PRMT5*/*WDR77* expression levels in T cells and response to anti–PD-1 therapy, indicating that this axis may represent a potential target for sensitizing tumors to immune checkpoint blockade.

## Discussion

A few studies for posttranslational regulation of PD-1 have provided potential intervention targets to enhance immunotherapeutic efficacy (7–10). However, at RNA level, it is yet unclear regarding the underlying mechanisms by which *PDCD1* is dysregulated and potential therapeutic regimens in T cells. Here, using the *PDCD1* 3′ UTR as a bait, we unexpectedly uncover that PRMT5 functions as a noncanonical RBP to inhibit *PDCD1* stability in a WDR77-dependent manner. T cell–specific deletion of PRMT5 inﬂuences T cell development, differentiation and proliferation, all of which were linked to its methyltransferase activity (55–57). Whereas little is known concerning the roles and mechanistic details for PRMT5 in T cell effector function. Our study provides unexpected roles and mechanism for PRMT5 in RNA binding, T cell effector function, and antitumor immunity. The function of WDR77 in T cell biology is yet unknown, we observed that the roles of WDR77 in peripheral homeostasis and T cell effector function are similar to PRMT5, further highlighting a synergetic action for PRMT5 and WDR77. However, how WDR77 promotes PRMT5/AGO2-mediated regulation of *PDCD1* RNA stability will need to be evaluated in the future. Additionally, tamoxifen-inducible *Prmt5^CKO^* or *Wdr77^CKO^* mice would provide a more precise assessment of PRMT5/WDR77–PD-1 axis’s role in effector T cells independently of developmental context.

Extrinsic stimulus including cytokine signaling usually regulate PD-1 expression during immune responses (2). IFN-α and STAT1 were found to drive PD-1 transcription in macrophages or T cells (58, 59). IFN-β increased the expression of PD-1 on melanoma TILs (60). However, in hepatic cytotoxic T lymphocytes, IFN-I (IFN-α receptor) signaling suppressed PD-1 expression, while IFN-β exerted no influence on PD-1 expression (61). These discrepancies may be due to the diversity of physiological or pathogenic situation. We reveal that when PRMT5 or WDR77 is insufficient in T cells, type I IFN/STAT1 suppress T cell–intrinsic PD-1, suggesting that the regulation of type I IFN on PD-1 expression in T cells may depend on the PRMT5/WDR77 status. Fludarabine is well known for its immunosuppressive effects on T lymphocytes (62, 63); however, emerging evidences suggest that it may also enhance antitumor immunity (45, 46, 64, 65). Here, we propose that fludarabine, by targeting STAT1, in combination with PD-1 blockade, represents a potential strategy to potentiate cancer immunotherapy. Nonetheless, further studies are required to fully evaluate its safety and efficacy before clinical application.

In summary, our work shows a previously unrecognized function for PRMT5 as an RBP, establishes a regulatory mechanism of PD-1 at the posttranscriptional level and demonstrates the crucial role of this mechanism in T cell effector function and antitumor immunity (Figure 7K). This study shed light on a potential cancer immunotherapy strategy.

## Methods

### Sex as a biological variable.

Both female and male mice were included in this study, and similar findings are reported for both sexes.

### Cell culture.

The Jurkat (human acute leukemia T-cell line) and HEK293T (human embryonic kidney) cell lines were purchased from the Shanghai Cell Bank, Chinese Academy of Sciences. The EL4 cell line (murine T-cell lymphoma) was sourced from Bluefcell Technology (Shanghai, China). The Plate-E retroviral packaging cell line was purchased from Meisen Chinese Tissue Culture Collections. MC38 and B16F10 cell lines were sourced from BeNa Culture Collection (Beijing, China). Two heterozygous PRMT5 or WDR77 knockout (KO) Jurkat cell lines were independently generated by Cyagen Biosciences (Suzhou, China) using CRISPR-Cas9 technology. Jurkat and MC38 cells were cultured in RPMI1640 medium supplemented with 10% fetal bovine serum (FBS, Biochannel Biotechnology Co. Ltd.). HEK293T, Plate-E, B16F10 and EL4 cells were maintained in DMEM medium with 10% FBS. All cell lines were verified using short tandem repeat assays for identification and were cultured at 37°C in a humidified incubator with 5% CO2. These cell lines were tested negative for Mycoplasma contamination.

### T cell isolation.

Human PBMCs were isolated using Ficoll-Hypaque (Dakewe Biotech) centrifugation from blood samples obtained from healthy volunteers. Human CD8^+^ T cells were enriched from fresh PBMCs by negative selection using magnetic microbeads (STEMCELL Technologies, #17953). Isolated CD8^+^ T cells were cultured in RPMI1640 medium supplemented with 10% FBS and stimulated with 2 μg/mL anti-human CD3 (eBioscience, clone UCHT1) and 2 μg/mL anti-human CD28 (eBioscience, clone CD28.2), along with 50 U/mL IL-2, for the indicated duration. Mouse T cells, naive CD8^+^ or CD4^+^ cells were isolated from mouse spleens or lymph nodes by negative selection using magnetic beads (STEMCELL Technologies, #19851/#19853/#19852A). These cells were cultured in RPMI1640 medium containing 10% FBS, 0.05 mM 2-mercaptoethanol, 2 mM L-glutamine, 1 mM sodium pyruvate, 2 μg/mL anti-mouse CD3 (eBioscience, clone 145-2C11), and 2 μg/mL anti-mouse CD28 (eBioscience, clone 37.51) in the presence of 10 ng/mL IL-2, as indicated.

### Mice.

*Prmt5^fl/fl^* or *Wdr77^fl/fl^* mice were generated by Cyagen Biosciences (Suzhou, China) using CRISPR-Cas9 gene-targeting technology. These mice were crossed with *CD4^cre^* transgenic mice (Cyagen Biosciences) to obtain *Prmt5^CKO^* and *Wdr77^CKO^* mice, in which PRMT5 or WDR77 is completely deleted (homozygous deletion) in T cells, and *Prmt5^CKD^* and *Wdr77^CKD^* mice, in which PRMT5 or WDR77 is partially deleted (heterozygous deletion) in T cells (Supplemental Figure 10, A–D). Littermate controls with normal PRMT5 (*Prmt5^fl/fl^*) and WDR77 (*Wdr77^fl/fl^*) expression were used as their respective WT controls. C57BL/6J mice were purchased from Vital River Laboratories Co. Ltd. For animal studies, 6–8 week-old adult mice were used, and the mice were randomly assigned to experimental groups. All mice were housed in individually ventilated cages (IVC) in a specific pathogen-free (SPF) environment at room temperature (RT) and 50%–60% relative humidity at the animal facility of the Advanced Research Institute for Life and Health, Southeast University.

### Flow cytometry assay (FACS).

To detect the cell surface levels of PD-1 on Jurkat cells, cells were blocked with Human TruStain FcX Fc Receptor Blocking Solution (Biolegend, #422302) and incubated with APC-conjugated Mouse anti-Human CD279 (BD Biosciences, #558694) for 30 minutes at room temperature (RT) in the dark. After washing twice with PBS containing 1% FBS, cells were analyzed by FACS. For the cell surface levels of Pd-1 on EL4 cells and mouse CD8^+^ or CD4^+^ T cells, cells were blocked with purified anti-mouse CD16/32 antibody (Biolegend, #101302) and incubated with APC-conjugated anti-mouse PD-1 antibody (Biolegend, #329907) for 30 minutes at RT in the dark. After washing two times with PBS buffer containing 1% FBS, cells were analyzed by FACS. To analyze the phenotype of tumor-infiltrating lymphocytes (TILs), tumor tissues were cut into pieces and shredded into single cells. TILs from MC38 tumors were isolated using Mouse Tumor-Infiltrating Lymphocyte Separation Medium (Solarbio, #P9000) and stimulated with Cell Activation Cocktail (with Brefeldin A) (Biolegend, #423303) for 4 hours at 37°C. TILs were then analyzed by cell surface staining for CD45, CD3, CD4, CD8, Pd-1, Foxp3, and Prmt5, as well as intracellular staining for IFN-γ, TNF-α, and granzyme B (Gzmb).

### Statistics.

Data was presented as the mean ± SEM or SD. Two-tailed Student’s *t* test was performed to compare 2 groups. One-way ANOVA or 2-way ANOVA was used to compare more than 2 groups. Spearman correlation was performed to analyze the correlation. RNA stability at the end point was analyzed using 2-sided *t* tests or 1-way ANOVA. *P* < 0.05 was considered statistically significant. All statistical analyses were performed using GraphPad Prism 8.0.

### Study approval.

All animal experiments were approved by the Institutional Animal Care and Use Committee of Southeast University and complied with relevant ethical regulations.

### Data availability.

UV cross-linking RIP-seq data were uploaded to the Gene Expression Omnibus (GEO) database under accession number GSE276385. Values for all data points in graphs are available in the Supporting Data Values file.

Additional methods can be found in the Supplementary Methods.

## Author contributions

YG and YP contributed equally to this work. SG and YG designed research, wrote the paper. YG, YP, CP, ZT, YC, HZ, XW, CH, FY, X Zhu, QZ, and YZ performed research. YG, YP, QP, and ZT analyzed data. X Zhao and SG reviewed the paper.

## Funding support

- The National Natural Science Foundation of China (82203348, 82025029, 82430094).
- Basic Research Program of Jiangsu (BK20253033).
- Start-up Research Fund of Southeast University (RF1028623018, RF1028625166).
- Jiangsu Funding Program for Excellent Postdoctoral Talent (2022ZB106).
- New Round of Xuzhou “Pengcheng Talent Program” - High-level Healthcare Talent Recruitment and Development Project (Project Number: 2025TD07, 2025TD09).

## Supplementary Material

Supplemental data

Unedited blot and gel images

Supplemental table 1

Supplemental table 2

Supporting data values

## Version 1 02/02/2026

Electronic publication
