# Supplementary material for: Posttranscriptional regulation of PD-1 by PRMT5/WDR77 complex shapes T cell effector function and antitumor immunity
Source: J Clin Invest. 2026 Feb 2;136(3):e191469. doi: 10.1172/JCI191469 (PMC12867141; doi:10.1172/JCI191469)
Supplement: Supplemental data [file jci-136-191469-s364.pdf]

Post-transcriptional regulation of PD-1 by PRMT5/WDR77 complex shapes T cell effector function and anti-tumor immunity

This supplementary information contains:

- Supplementary Methods
- Supplementary Figures (18 Figures)
- Supplementary Tables (8 Tables)
- References

Lentivirus and Retrovirus Transduction

For lentivirus production, HEK293T cells were seeded in 10-cm dishes at 60–70% confluency and transfected with lentiviral packaging plasmids (3 μg pMD2.G and 6 μg psPAX2) and 8 μg pSIH1-H1-Puro shRNA vector (knockdown, KD) or pLVX-IRES-Neo vector (overexpression, OE) using polyethyleneimine reagent. For retrovirus production, Plate-E cells were transfected with 4 μg pCL-Eco packaging vector and MSCV-IRES-GFP vector (OE) using Lipofectamine 8000 reagent (Beyotime, #C0533), according to the manufacturer's instructions. Virus-containing supernatants were collected 48 hours (h) post-transfection. Jurkat cells, human $CD8^+$ T cells, and EL4 cells were incubated with the viral supernatants for 48 h. The transfected Jurkat and EL4 cells were stimulated with 2 μg/mL and 500 ng/ mL phytohaemagglutinin (PHA) for 48 h. The transfected human $CD8^+$ T cells were stimulated with 2 μg/mL anti-human CD3 and 2 μg/mL anti-human CD28. Short hairpin RNA (shRNA) target sequences are listed in Supplementary Table 3.

Luciferase Reporter Assay

The luciferase reporter assay was performed as previously described.[1] Wild-type (WT), fragment, or mutant sequences of the *PDCD1* or *Pdcd1* 3' UTR were cloned downstream of the firefly luciferase (*F-Luc*) gene in a pmirGLO Dual-Luciferase miRNA Target Expression Vector. HEK293T cells were seeded in a 24-well plate and co-transfected with the indicated pmirGLO-3' UTR plasmid along with either a PRMT5 or WDR77 KD vector, or a control vector. The WT promoter constructs of *PRMT5* or *WDR77* (−1000 to +500 bp relative to their transcription start site) were cloned upstream of the *F-Luc* reporter gene in a pGL3 vector. HEK293T cells were co-transfected with the indicated pGL3-*PRMT5* or *WDR77* promoter plasmid, a pRL-TK Renilla luciferase (*R-Luc*) reporter plasmid, and an expression vector encoding STAT1 or a control vector. For tethering assays, the pmirGLO reporter plasmid containing the 3' UTR of *PDCD1* or *Pdcd1* with 5 BoxB was co-transfected with λN-FLAG-PRMT5-WT

or mutant expression vectors. 48h post-transfection, F-Luc and R-Luc activities were measured using the Dual-Luciferase Reporter Gene Assay Kit (Yeasen, China). F-Luc activity was normalized to R-Luc activity, and mRNA abundance was determined by qPCR for both F-*Luc* and *R-Luc*.

Western Blot Analysis

Proteins were extracted from the indicated cells using RIPA lysis buffer (20 mM Tris–HCl at pH 7.5, 150 mM NaCl, 1% Triton X-100, 2 mM EDTA, 0.5 mM DTT, and 1:100 protease inhibitor cocktail). Proteins were resolved by SDS-PAGE and transferred to PVDF membranes. Membranes were blocked with 5% nonfat milk and incubated with primary antibody solution (Supplementary Table 4). After hybridization with HRP-conjugated secondary antibodies, protein bands were visualized using BeyoECL Moon reagent (Beyotime, China).

MS2-Tagged RNA Affinity Purification

WT, fragment, or mutant sequences of the *PDCD1* or *Pdcd1* 3' UTR were inserted into the pcDNA3.1-24×MS2-stemloop plasmid. HEK293T cells were seeded in 10 cm dishes and co-transfected with 8 μg pcDNA3.1-24×MS2-based plasmid and 8 μg pcDNA3.1-FLAG-MS2 using JetPrime™ transfection reagent (Polyplus, 101000027). After 48-72 h, transfected cells were collected for RNA-binding protein pull-down. Cells were lysed with 500 μL lysis buffer (10 mM Tris–HCl at pH 7.4, 150 mM NaCl, 1% Nonidet P-40, 1 mM EDTA, 0.1% SDS, and 1 mM DTT). 2 mg of lysate was incubated with magnetic anti-FLAG beads (Sigma, #M8823) for 3 h at 4 °C in NT2 buffer (50 mM Tris–HCl at pH 7.5, 150 mM NaCl, 1 mM $MgCl_2$, and 0.05% NP-40). After washing with NT2 buffer five times, the complexes were eluted from the beads using 80 μL 3×Flag Peptide (Beyotime, China) for 1 h at 4 °C. The eluted proteins were analyzed by SDS-PAGE followed by silver staining using the Pierce Silver Stain Kit (Beyotime, China) or western blotting.

Co-Immunoprecipitation (Co-IP)

HEK293T cells expressing FLAG-tagged PRMT5 or HA-tagged WDR77 were transfected with siRNAs targeting the *PDCD1* or pcDNA3.1-*PDCD1* 3' UTR. After 48-72 h, transfected cells were lysed using cell lysis buffer for western blotting and immunoprecipitation (Beyotime, #P0013), supplemented with protease inhibitor cocktail. 1 mg of lysate was incubated with magnetic anti-FLAG beads (Sigma, #M8823) or anti-HA beads (Thermo Scientific, #88837) at 4 °C overnight. The immunoprecipitates were washed five times with lysis buffer, boiled in SDS loading buffer, and analyzed by western blotting. The siRNAs used are listed in Supplementary Table 3.

RNA Extraction and Quantitative Real-Time PCR (qPCR)

Total RNA was extracted from the indicated cells and reverse-transcribed into cDNA using HiScript III All-in-one RT SuperMix Perfect for qPCR (Vazyme, China). qPCR was performed on an Applied Biosystems QuantStudio™ 7 Flex Real-Time PCR System (Applied BioSystems, ThermoFisher Scientific) with Taq Pro Universal SYBR qPCR Master Mix (Vazyme, China). Data were normalized to the expression of *GAPDH* or *18S*. Relative gene expression levels were analyzed using the $2^{-\Delta\Delta CT}$ method.[2] The sequences of the primers are listed in Supplementary Table 5.

RNA Immunoprecipitation (RIP)

Cells were seeded in 10 cm dishes at 70–80% confluency, cross-linked with UV light (254 nm) on ice, and lysed in NT2 buffer (200 mM Nacl, 50 mM HEPES at pH 7.6, 2 mM EDTA, 0.05% NP-40, 0.5 mM DTT, RNase inhibitors) at 4 °C for 30 min. 2 µg PRMT5 (Thermo Fisher Scientific, #A300-849A), WDR77 (Thermo Fisher Scientific, #A301-562A), FLAG (Proteintech, #66008-4-Ig) antibodies, or corresponding control IgG (Proteintech, #30000-0-AP for Rabbit, #B900620 for

Mouse) were conjugated to Protein A/G Magnetic Beads by incubation for 1 h at 4 °C. The lysate supernatant was incubated with the antibody-conjugated Protein A/G Magnetic Beads in 500 μL NT2 buffer supplemented with RNase inhibitors at 4 °C overnight. After washing with RIP buffer for five times, the beads were resuspended in TRIzol for RNA extraction and analyzed by qPCR. Primer sequences for RIP-qPCR are listed in Supplementary Table 6.

Chromatin Immunoprecipitation (ChIP)

The ChIP assay was performed as previously described[3] using the Magna ChIP HiSens Chromatin IP Kit (Merck Millipore), according to the manufacturer's instructions. Briefly, Jurkat cells were treated with 50 ng/mL IFN-α or IFN-β for 48 h. Cells were washed twice with ice-cold PBS, cross-linked with 4% formaldehyde for 10 min at 37°C, and sonicated using a Covaris M220 to generate 200-500 bp DNA fragments. Immunoprecipitation was performed using an anti-STAT1 antibody (Proteintech, #10144-2-AP). The binding of STAT1 to *PRMT5* or *WDR77* promoter was quantified by qPCR. The specific primers for ChIP-qPCR are listed in Supplementary Table 7.

CRISPR-Assisted RNA-Protein Interaction Detection (CARPID)

The CARPID assay was conducted as described previously[4]. gRNAs targeting specific regions of *PDCD1* were cloned into the CasRx pre-gRNA backbone plasmid (Addgene #109054). To evaluate the specificity of selected gRNA sets, HEK293T cells were co-transfected with the indicated CasRx pre-gRNA plasmids and wild-type CasRx nuclease using Lipofectamine 8000 reagent (Beyotime, #C0533). At 72 h post-transfection, qPCR was performed to determine the knockdown efficiency. For the CARPID assay, HEK293T cells were seeded in 10 cm dishes and co-transfected with the indicated CasRx pre-gRNA plasmids and the BASU-dCasRx plasmid. After 48-72 h, the medium was replaced with fresh medium containing 200 μM biotin, and the cells were

incubated for 15 min at 37°C with 5% CO_2. The cells were then washed three times with PBS to remove residual biotin. Cells were lysed in RIPA buffer at 4 °C for 30 min and incubated with 30 μL of MyOne T1 Streptavidin Beads (Thermo Fisher Scientific, #65601) at 4 °C for 3 h. After washing with lysis buffer for five times, the complexes were boiled in SDS loading buffer and analyzed by western blotting. The sequences of the gRNAs are listed in Supplementary Table 8.

RNA Stability and RNA Decay

For RNA stability assays, the indicated HEK293T, Jurkat, and mouse $CD8^+$ T cells were treated with 5 μM actinomycin D (Act D) for the indicated times. Total RNA was extracted using TRIzol, and mRNA levels were analyzed by qPCR. For RNA decay assays, Jurkat cells were seeded in 10 cm dishes and incubated with 100 μM 4-thiouridine (4sU, Sigma) for 2 h. After the pulse-chase labeling, the medium was replaced, and cells were collected at the indicated times. Total RNA was extracted, and 100 μg of 4sU-labeled total RNA was incubated in biotinylation buffer (10 mM Tris at pH 7.4, 1 mM EDTA) with 0.2 mg/mL EZ-Link Biotin-HPDP (Thermo Fisher Pierce, #21341) at room temperature (RT) in the dark for 1.5 h with rotation. RNA was precipitated by centrifugation at 13,000 rpm for 15 min at 4°C using 1:10 volume of 5 M NaCl and an equal volume of isopropanol. The RNA pellet was washed twice with 75% ethanol and resuspended in DEPC-treated H_2O. 4sU-labeled and biotinylated RNA was separated using streptavidin beads at RT for 30 min. Beads were washed four times with washing buffer (100 mM Tris at pH 7.4, 10 mM EDTA, 1 M NaCl, 0.1% Tween 20). Nascent RNA was eluted twice with 100 μL 0.1 M dithiothreitol (DTT), and RNA was precipitated with 40 μL of 4 M LiCl, 2 μL glycogen, and 600 μL ice-cold ethanol. RNA levels were analyzed by qPCR.

Oligo(dT) Pull-Down

Oligo(dT)-25 magnetic beads (NEB, #S1419S) were swelled in wash buffer (20 mM Tris-HCl at pH 7.4, 250 mM NaCl, 10 mM KCl, 5 mM MgCl2, RNase inhibitors) at 4°C for 1 h with rotation. Cell lysates from HEK293T, Jurkat, EL4, and mouse CD8$^+$ T cells were washed twice with ice-cold PBS and lysed in lysis buffer (20 mM Tris-HCl at pH 7.4, 250 mM NaCl, 10 mM KCl, 5 mM $MgCl_2$, 0.1% Triton X-100, RNase inhibitors, and protease inhibitors) on ice for 30 min. Between 500 μg and 1 mg of lysate were added to the swelled Oligo(dT) beads, and the samples were rotated end-over-end for 3 h at 4°C. Beads were washed five times with 5×wash buffer, boiled in SDS loading buffer, and analyzed by western blotting.

Orthogonal Organic Phase Separation (OOPS)

The OOPS assay was performed as previously described[5]. HEK293T, Jurkat, EL4, and mouse T cells were cultured in 10 cm dishes at 80–90% confluency and washed twice with PBS. For non-crosslinked controls, cells were lysed immediately in TRIzol reagent. For crosslinked samples, UV crosslinking was performed on PBS-washed cells by UV irradiation at 254 nm. Immediately after crosslinking, cells were lysed in TRIzol and incubated at RT for 5 min to dissociate unstable RNA-protein interactions. Then, 200 μL chloroform was added, and the samples were vortexed and centrifuged for 15 min at 13,000×g at 4°C. The upper aqueous phase (containing non-crosslinked RNAs) and the lower organic phase (containing non-crosslinked proteins) were discarded. The interface containing RNA-protein adducts was subjected to another round of TRIzol and chloroform extraction, precipitated by adding 9 volumes of methanol, and pelleted by centrifugation at 14,000×g at RT for 10 min. The precipitated interface was resuspended in 100 μL buffer (100 mM TEAB, 1 mM MgCl2, 1% SDS), incubated at 95°C for 20 min, cooled down and digested with 2 μg RNase A/T1 mix (2 mg/mL RNase A, 5000 U/mL RNase T1) for 2-3 h at 37°C. Released proteins were recovered from the organic phase by methanol precipitation,

boiled in SDS loading buffer, and analyzed by western blotting.

Capture of the Newly Transcribed RNA Interactome Using Click Chemistry (RICK)

HEK293T and Jurkat cells were cultured in 10 cm dishes until 80% confluence. Cells were then treated with 5-ethynyluridine (EU, RiboBio, #C00065) for 16 h. After washing three times with PBS, cells were UV crosslinked at 254 nm, fixed with 90% ethanol for 30 min, washed three times with PBS, and permeabilized with 0.5% Triton X-100 for 15 min. After three times washing with PBS, permeabilized cells were incubated with 10 mL click reaction buffer supplemented with 0.6 mM THPTA (Sigma-Aldrich, #762342) and 1 mM aminoguanidine hydrochloride (Sigma-Aldrich, #396494) for 3 min. The reaction was stopped by washing the cells with 0.5% Triton X-100 supplemented with 2 mM EDTA. Cells were then lysed in lysis buffer (20 mM Tris-HCl at pH 7.5, 500 mM LiCl, 1 mM EDTA at pH 8.0, 0.5% LiDS, 5 mM DTT) with protease and RNase inhibitors added. Cell lysates were isolated with Dynabeads MyOne Streptavidin C1 Beads (Thermo Fisher Scientific, # 65001). After incubating with the lysates for 2 h with rotation, beads were washed with buffer 1 (20 mM Tris–HCl at pH 7.5, 500 mM LiCl, 1 mM EDTA at pH 8.0, 0.1% LiDS, and 5 mM DTT), buffer 2 (20 mM Tris–HCl at pH 7.5, 500 mM LiCl, 1 mM EDTA at pH 8.0, and 5 mM DTT), and buffer 3 (20 mM Tris–HCl at pH 7.5, 200 mM LiCl, 1 mM EDTA at pH 8.0, and 5 mM DTT) for two times, then boiled in SDS loading buffer and analyzed by western blotting.

Electrophoretic Mobility Shift Assay (EMSA)

3′ biotin-labeled *PDCD1*/*Pdcd1* 3' UTR probes were synthesized by Hippo Co., Ltd. (Huzhou, China). The biotin-labeled RNAs were annealed by heating at 65°C for 5 min and then slowly cooled to RT. 10 pmol of labeled RNA probes were used for each EMSA reaction. Purified WT or mutant FLAG-tagged

PRMT5 or WDR77 proteins were generated by an *in vitro* transcription-translation reaction using TNT T7-coupled rabbit reticulocyte lysate (Promega, #L1170). EMSA was performed with the chemiluminescent EMSA Kit (Beyotime, #GS009). Negative control and competitor control conditions were applied as per the manufacturer's instructions. The sequences of the probes are listed in Supplementary Table 9.

T Cell Effector Function Analysis

CD8$^+$ and CD4$^+$ T cells were isolated from mice and stimulated with 2 μg/mL anti-CD3 and anti-CD28 in the presence of 10 ng/mL IL-2, and treated with anti-PD-1 (Bioxcell, #J43) or IgG antibody for 72 h. T cell death was detected using 7-Aminoactinomycin D (7-AAD, BD Biosciences, #559925). T cell proliferation was detected using anti-Ki67. T cell activation was detected using anti-CD44. T cell cytotoxicity was detected using anti-granzyme B (Gzmb). To measure cytokine secretion (including IFN-γ, TNF-α, IL-2, IL-4, and IL-17α), cells were treated with Brefeldin A for 4-6 h to block cytokine transportation before harvesting. Cells were then stained with anti-IFN-γ, anti-TNF-α, anti-IL-2, anti-IL-4, and anti-IL-17α for FACS analysis. The used fluorescent antibodies for FACS are listed in Supplementary Table 10.

In Vitro Co-Culture Tumor Killing Assay

CD8$^+$ T cells isolated from the spleens of OT-I mice were stimulated with 1 μg/mL OVA peptide in the presence of 10 ng/mL IL-2 for 48 h. For the specific cytotoxicity assay, MC38-OVA tumor cells (target) were plated at 0.5×10^5 cells per well in a 48-well plate. OT-1 CD8$^+$ T cells (effector) were then added at a 5:1 effector:target ratio. The coculture was incubated for 36 h, after which the cells were collected, resuspended in Annexin V Binding Buffer, and stained with Annexin V and 7-AAD viability solution (BioLegend) for 15 min at 25°C.

Mouse Tumor Models and Therapeutic Treatments

Age- and sex-matched *Prmt5*^{CKO} or *Wdr77*^{CKO} mice and their WT controls (6–8 weeks old) were inoculated subcutaneously with 2×10^5 MC38 or B16F10 cells in the dorsal area. For anti-PD-1 antibody treatments, mice bearing subcutaneous MC38 tumors (around 50 mm^3) were pooled and randomly divided into the indicated groups. Seven days after MC38 inoculation, mice were intraperitoneally injected with 100 µg IgG (Selleck, #A2123) or 100 µg anti-PD-1 (Selleck, #A2122) every three days (four times in total), and tumor sizes were recorded every two days. For fludarabine treatments, mice bearing subcutaneous MC38 tumors were pooled and randomly divided into the indicated groups. Fludarabine (Selleck, #S1491) treatment was administered via intraperitoneal injections at 35 mg/kg every three days (three times in total). Tumor size was measured over time and calculated as length × ($width^2$)/2. Mouse survival was monitored daily. Tumor-bearing mice were euthanized when the tumor size exceeded 2,000 mm^3 or the tumor size was larger than 18 mm in any direction.

Single-cell RNA-seq Data Processing and Analysis

Single-cell RNA sequencing data were processed using the Seurat package. The expression matrix was normalized using the NormalizeData function. Subsequently, 2000 highly variable genes were identified with the FindVariableFeatures function under default parameters. The UMI counts and mitochondrial gene percentage were regressed out using the ScaleData function. Principal component analysis (PCA) was performed based on these variable genes. Batch effects across samples were corrected using the RunHarmony function from the Harmony package. Dimensionality reduction and visualization were conducted on the harmonized space via UMAP implemented in the RunUMAP function. Cell clusters were identified using the FindNeighbors and FindClusters functions, with a resolution parameter set to 1.

Cell clusters were annotated into specific cell states based on canonical marker genes. T cells were subsetted for further analysis. Differential expression analysis was then performed between patient groups from which the T cells were derived.

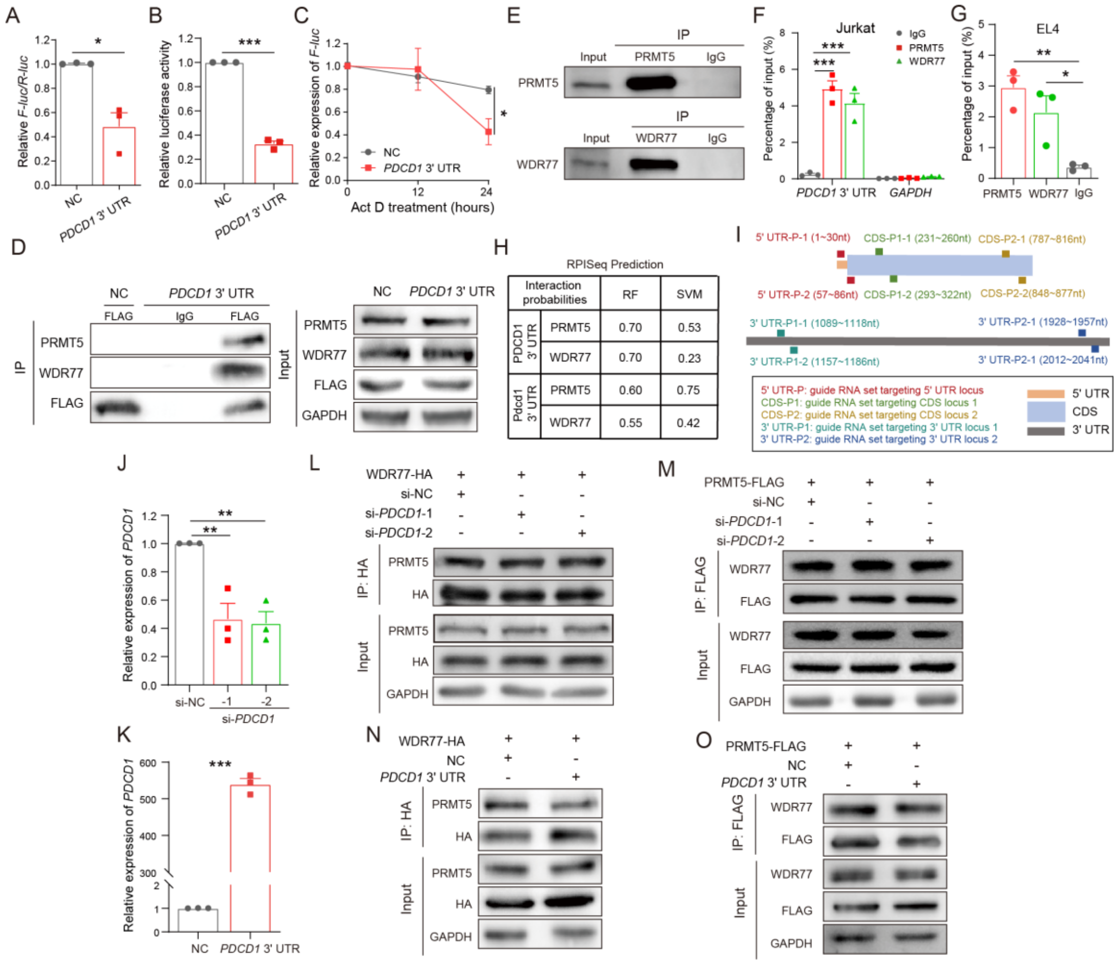

Supplemental Figure 1. *PDCD1* 3' UTRs is associated with the PRMT5 and WDR77. (A-C) qPCR showing mRNA abundance of *F-Luc* (A) or dual-luciferase assay showing relative luciferase activities (B) or RNA stability assay showing half-lives of *F-Luc* (C) in Jurkat cells expressing *PDCD1* 3' UTR reporter. ActD, Actinomycin D. NC, negative control. (D) Immunoblotting analysis of the specific association of PRMT5 and WDR77 with MS2 aptamer-tagged *PDCD1* 3' UTR in Jurkat cells. IP, immunoprecipitation. (E) Immunoblotting analysis of the precipitation efficiencies of anti-PRMT5 (top) and anti-WDR77 (bottom) in Jurkat cells. (F-G) RIP-qPCR analysis of *PDCD1* 3' UTR enriched by PRMT5 and WDR77 in Jurkat (F) and EL4 (G) cells. (H) RPIseq program predicting the binding affinities between PRMT5 or WDR77 and *PDCD1* or *Pdcd1* 3' UTRs using RPISeq-RF and RPISeq-SVM methods. Probabilities greater than 0.5 indicate a positive interaction. (I) The locations of the five sets of gRNAs on *PDCD1* for the CARPID assay. (J-O) qPCR assays (J and K) showing mRNA

levels of *PDCD1* or Co-IP assays (L-O) showing association between PRMT5 and WDR77 in NCI-H1299 cells using siRNAs to knock down endogenous *PDCD1* or 293T cells expressing the *PDCD1* 3' UTR. For A-C and K (n = 3), by unpaired two-tailed Student's t test; For F-G and J (n = 3), by one-way ANOVA with Dunnett's test. Data are presented as mean ± SEM or SD. *$p < 0.05$, **$p < 0.01$, ***$p < 0.001$.

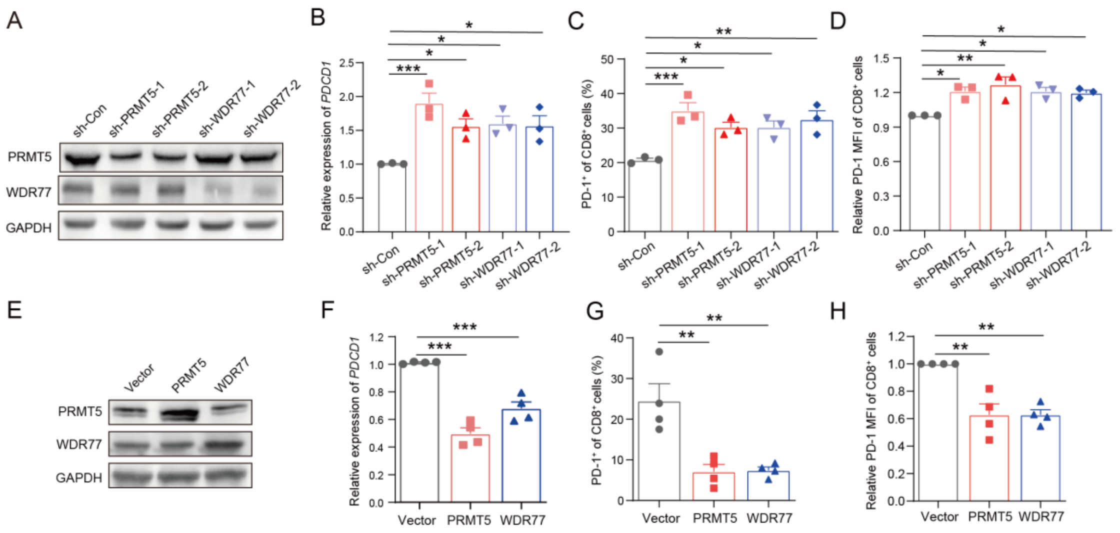

Supplemental Figure 2. PRMT5 and WDR77 suppress *PDCD1* expression. (A-H) Immunoblotting (A and E) showing expression of PRMT5 and WDR77 or qPCR assays (B and F) showing mRNA levels of *PDCD1* or FACS assays showing the percentages of positive cells (C and G) and relative MFI levels (D and H) of PD-1 in the indicated human $CD8^+$ cells stimulated with anti-CD3 and anti-CD28. For B-D (n = 3) and F-H (n = 4), by one-way ANOVA with Dunnett's test. Data are presented as mean ± SEM or SD. *$p < 0.05$, **$p < 0.01$, ***$p < 0.001$.

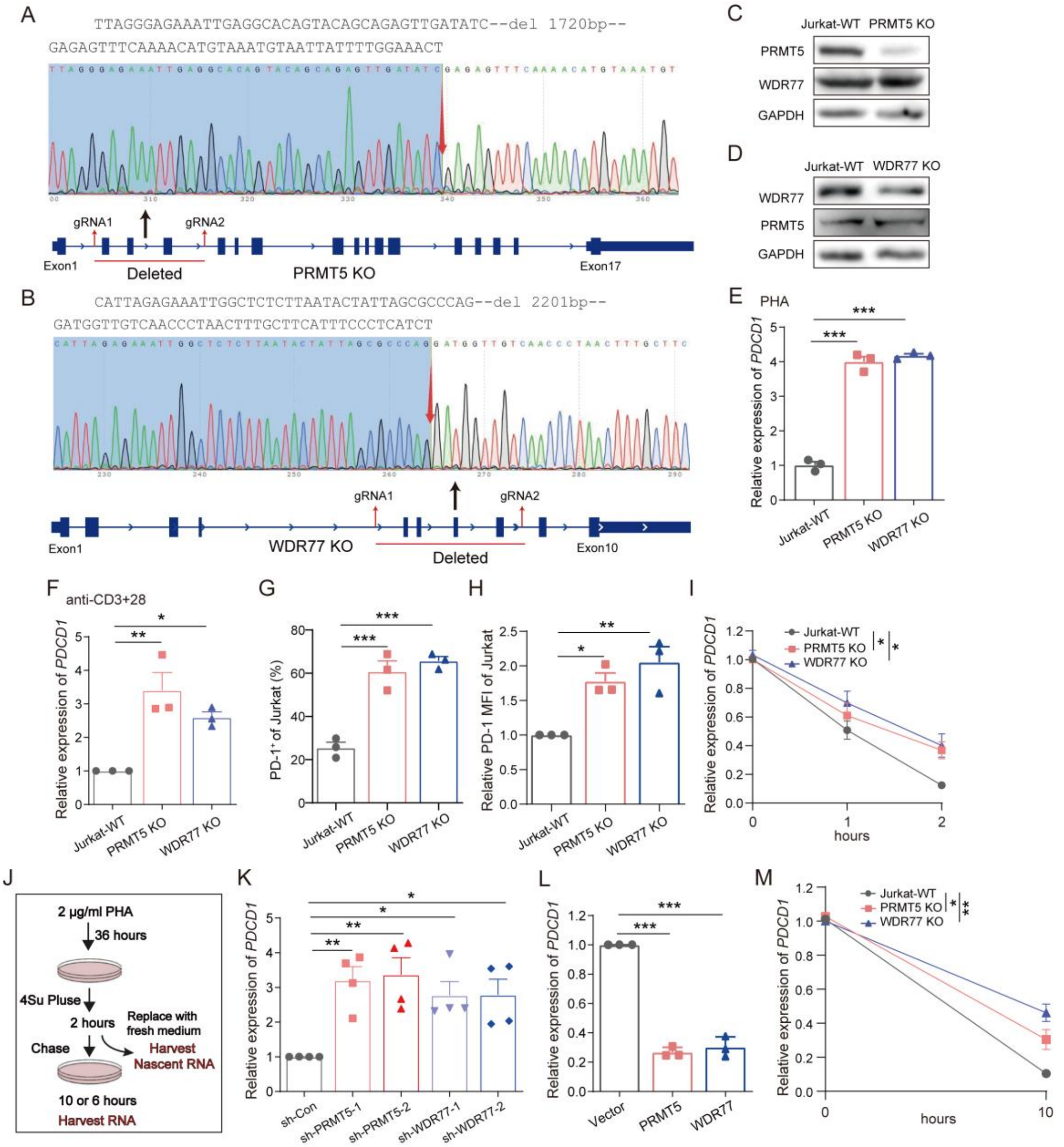

Supplemental Figure 3. The heterozygous KO of PRMT5 or WDR77 reduces *PDCD1* expression. (A-B) DNA sequence analysis of PRMT5 KO (A) and WDR77 KO (B) clones. (C-D) Immunoblotting analysis of PRMT5 and WDR77 expression in PRMT5 KO (C) and WDR77 KO (D) Jurkat cells. (E-F) qPCR assay showing mRNA levels of *PDCD1* in PRMT5 KO or WDR77 KO Jurkat cells stimulated with PHA (E) or anti-CD3 and anti-CD28 (F). (G and H) Percentages of positive cells (G) and relative MFI levels (H) of PD-1 in PRMT5 KO or WDR77 KO Jurkat cells stimulated with PHA. (I) RNA stability assay showing half-lives of *PDCD1* in PRMT5 KO or WDR77 KO Jurkat cells

stimulated with PHA. (J) Schematic diagram of the 4sU pulse-labeling and pulse-chase analysis workflow. (K and L) Nascent RNA levels of *PDCD1* in PRMT5 or WDR77 KD (K) and PRMT5 or WDR77 OE (L) Jurkat cells stimulated with PHA. (M) 4sU pulse-labeling and pulse-chase analysis of *PDCD1* RNA decay in PRMT5 or WDR77 KO Jurkat cells stimulated with PHA. For E-I and L-M (n = 3), for K (n = 4), by one-way ANOVA with Dunnett's test. Data are presented as mean ± SEM or SD. *$p < 0.05$, **$p < 0.01$, ***$p < 0.001$.

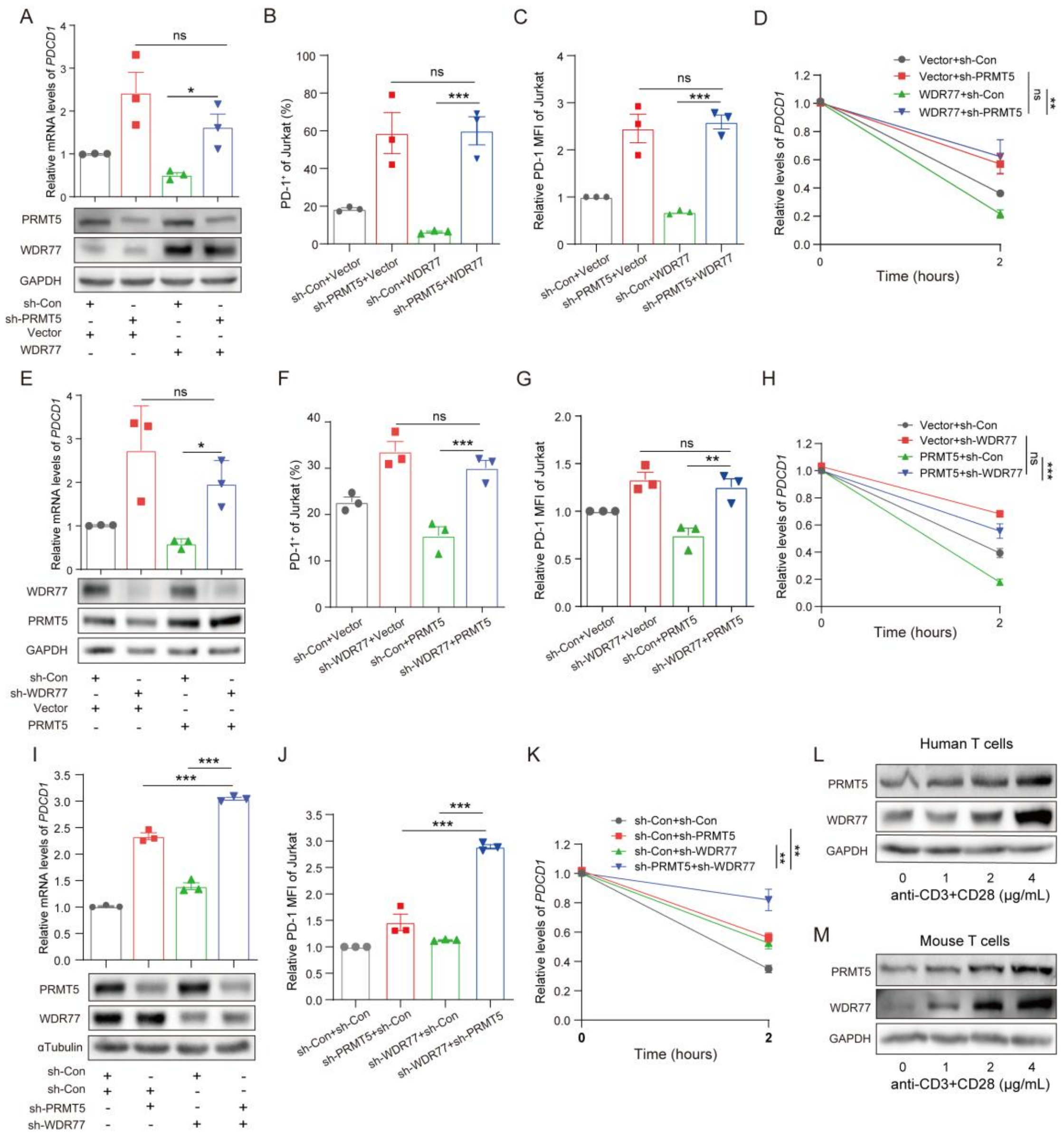

Supplemental Figure 4. PRMT5 and WDR77 cooperate to decrease *PDCD1* expression. (A-K) qPCR analysis (top) of *PDCD1* and immunoblotting (bottom) of PRMT5 and WDR77 (A, E and I) or FACS assays showing percentages of positive cells (B and F) and relative MFI levels (C, G and J) of PD-1 and RNA stability assays showing half-lives of *PDCD1* (D, H and K) in the indicated Jurkat cells stimulated with PHA. (L-M) Immunoblotting analysis of PRMT5 and WDR77 expression in Human (L) and mouse (M) $CD8^+$ T cells treated with anti-CD3+CD28. For A-K (n = 3), by one-way ANOVA with Bonferroni's test. Data are presented as mean ± SEM. *$p < 0.05$, **$p < 0.01$. ns, no significant.

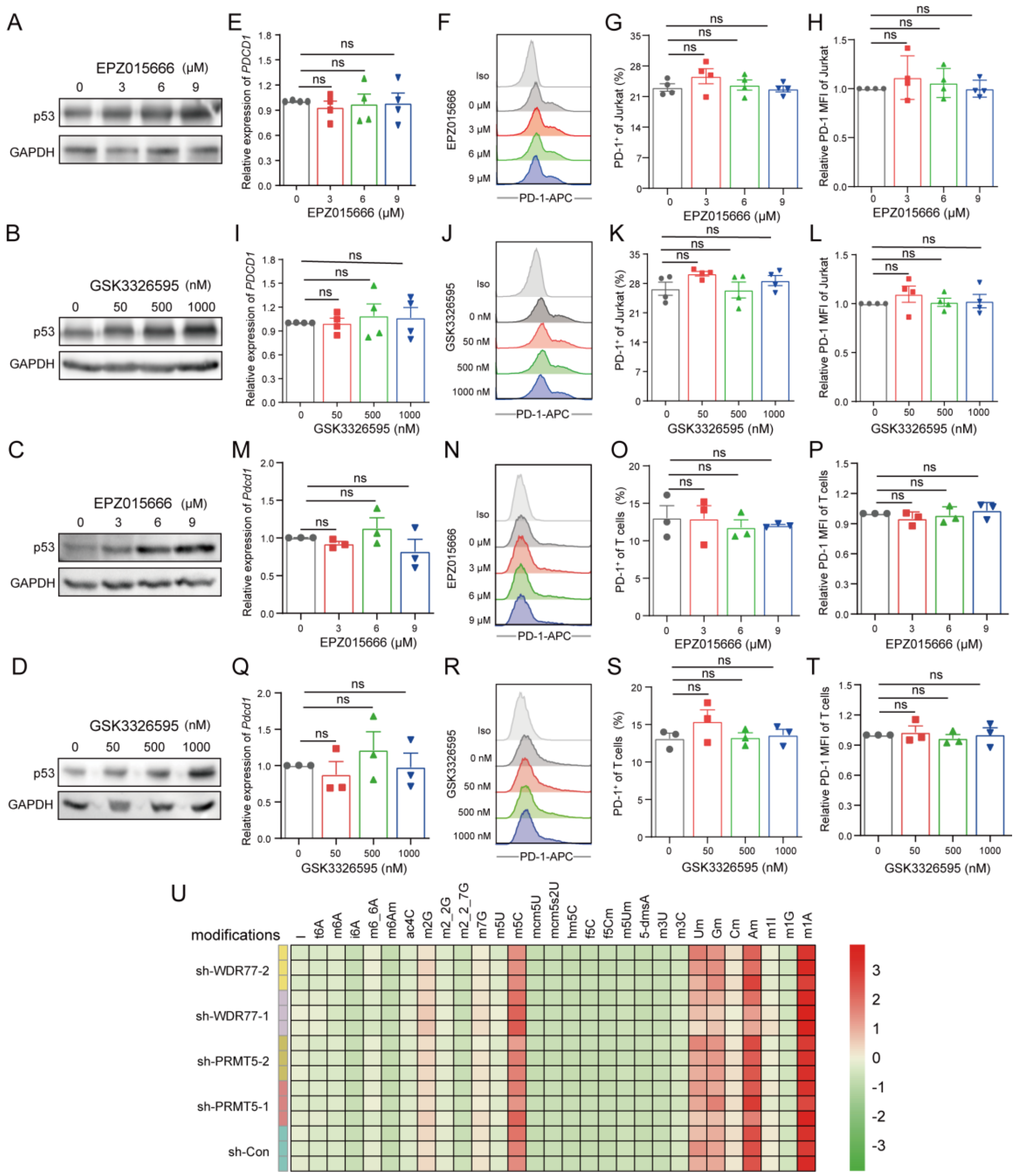

Supplemental Figure 5. PRMT5 regulates PD-1 expression independently of its methyltransferase activity. (A-D) Immunoblotting analysis of p53 in PHA-stimulated Jurkat cells (A and B) and anti-CD3/CD28-stimulated mouse T cells (C and D) treated with EPZ015666 or GSK3326595. (E-L) qPCR assays (E and I) showing mRNA levels of *PDCD1* and FACS assays showing the representative MFI plots (F and J), percentages of positive cells (G and K) and relative MFI levels (H and L) of PD-1 in PHA-stimulated Jurkat cells treated with EPZ015666 or GSK3326595. (M-T) qPCR assays (M and Q) showing the mRNA levels of *Pdcd1* and FACS assays showing representative MFI plots (N

and R), percentages of positive cells (O and S) and relative MFI levels (P and T) of PD-1 in anti-CD3/CD28-stimulated mouse T cells treated with EPZ015666 or GSK3326595. (U) Liquid chromatography-tandem mass spectrometry analysis of 29 different RNA modifications in PRMT5 KD or WDR77 KD HEK293T cells. For E-L (n = 4) and M-T (n = 3), by one-way ANOVA with Dunnett’s test. Data are presented as mean ± SEM or SD. ns, no significant.

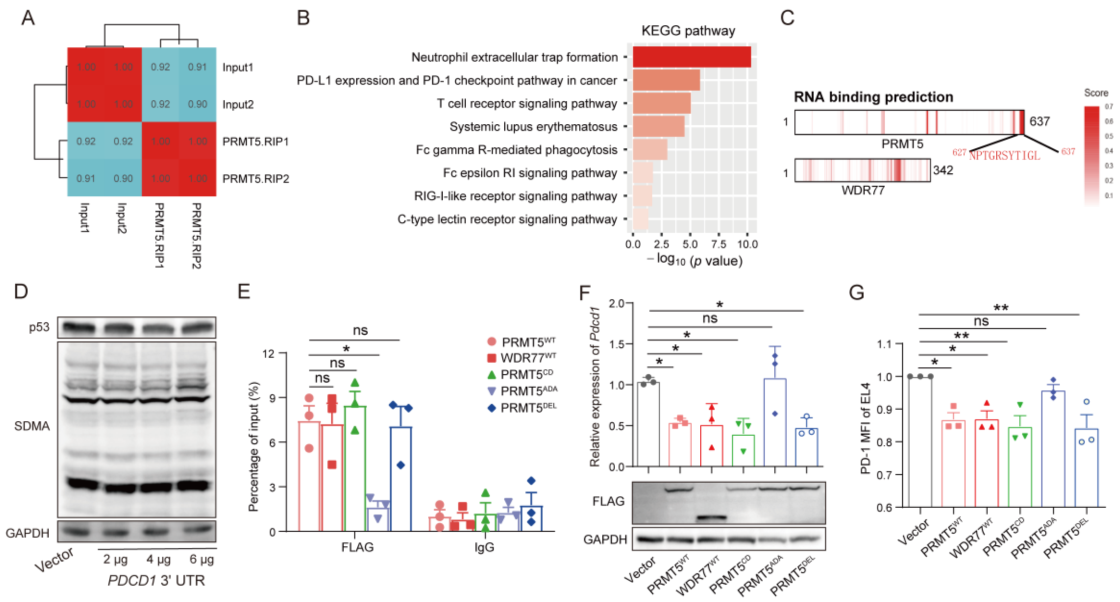

Supplemental Figure 6. PRMT5 suppresses PD-1 expression in an RBP-dependent manner.

(A) Correlation analysis of two replicate Jurkat cells in crosslinked RIP-sequencing analysis. (B) Kyoto Encyclopedia of Genes and Genomes (KEGG) pathway analysis of pathways of the immune system for PRMT5 targets. (C) RNABindRPlus prediction of the RNA-binding regions of PRMT5 and WDR77. (D) Immunoblotting analysis of p53 and symmetric dimethylarginine (SDMA) in HEK293T cells expressing the *PDCD1* 3' UTR. (E) RIP-qPCR analysis of *Pdcd1* 3' UTR enriched by FLAG in EL4 cells expressing the indicated constructs. (F) qPCR analysis (top) of *Pdcd1* and immunoblotting (bottom) of FLAG in PHA-stimulated EL4 cells expressing the indicated constructs. (G) Relative MFI levels of PD-1 in PHA-stimulated EL4 cells expressing the indicated constructs. For E-G (n = 3), by one-way ANOVA with Dunnett’s test. Data are presented as mean ± SEM or SD. *$p < 0.05$, **$p < 0.01$, ***$p < 0.001$. ns, no significant.

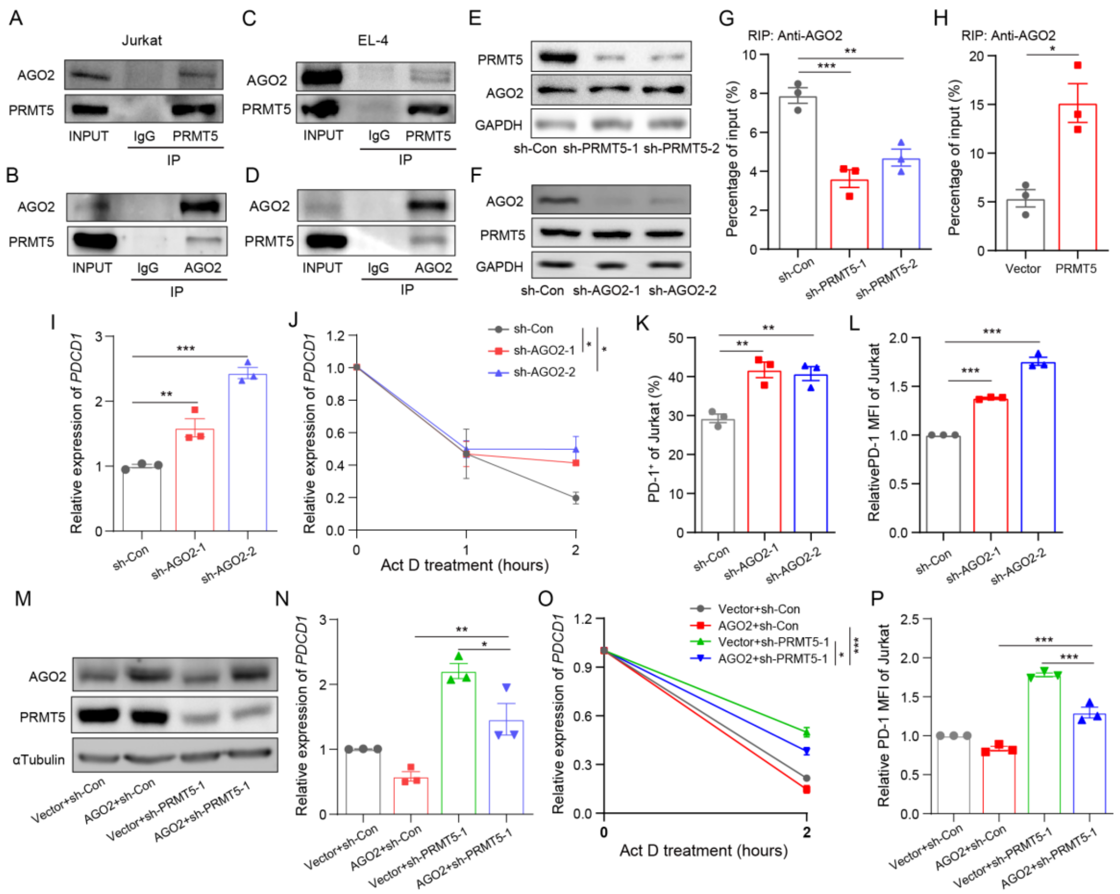

Supplemental Figure 7. PRMT5 recruits AGO2 to promote *PDCD1* mRNA degradation. (A-D) Co-IP assays showing association between PRMT5 and AGO2 in Jurkat (A-B) and EL-4 (C-D) cells. (E-F) Immunoblotting analysis of PRMT5 and AGO2 expression in PRMT5 KD (E) and AGO2 KD (F) Jurkat cells. (G and H) RIP-qPCR analysis of *PDCD1* 3' UTR enriched by AGO2 in PRMT5 KD (G) and OE (H) Jurkat cells. (I and J) qPCR assay (I) showing mRNA levels of *PDCD1* or RNA stability assay (J) showing half-lives of *PDCD1* in AGO2 KD Jurkat cells stimulated with PHA. (K and L) Percentages of positive cells (K) and relative MFI levels (L) of PD-1 in AGO2 KD Jurkat cells stimulated with PHA. (M-P) Immunoblotting of PRMT5 and AGO2 (M) or qPCR assay (N) showing mRNA levels of *PDCD1* or RNA stability assay (O) showing half-lives of *PDCD1* or FACS assays (P) showing relative MFI levels of PD-1 in the indicated Jurkat cells. For G and I-L (n = 3), by one-way ANOVA with Dunnett’s test; for H (n = 3), by unpaired two-tailed Student’s t test; for N-P (n = 3), by one-way ANOVA with Bonferroni’s test. Data are presented as mean ± SEM or SD. *$p < 0.05$, **p

< 0.01, ***$p < 0.001$. ns, no significant.

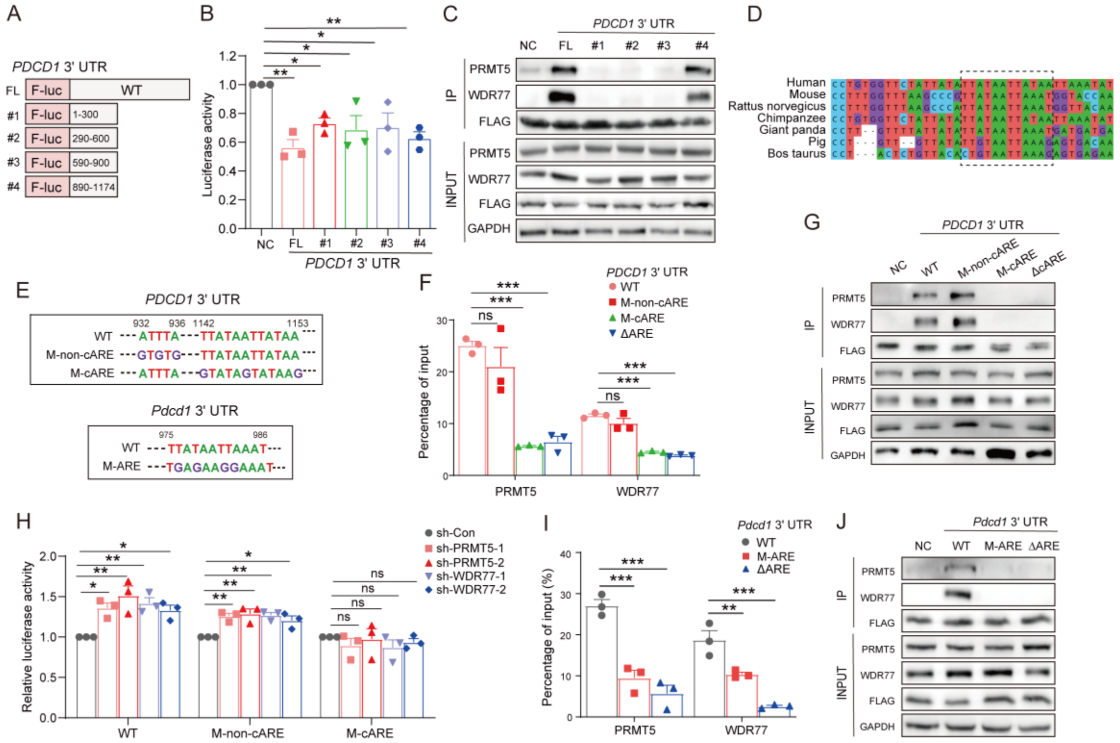

Supplemental Figure 8. A conserved ARE is critical for PRMT5 and WDR77 in degrading *PDCD1*. (A) Presentation of *PDCD1* 3’ UTR fragments. (B) Dual-luciferase assay showing relative luciferase activities in HEK293T cells expressing luciferase reporters of *PDCD1* 3' UTR fragments. (C) Immunoblotting analysis of the specific association of PRMT5 and WDR77 with *PDCD1* 3' UTR fragments in HEK293T cells expressing the indicated constructs. (D) Comparison of a *PDCD1* 3’ UTR fragment among different species. A non-classical ARE (cARE) is highlighted in the box. (E) Mutation strategies for the ARE sites of *PDCD1* or *Pdcd1* 3' UTRs. (F) RIP-qPCR analysis of *PDCD*1 3' UTR enriched by PRMT5 and WDR77 in HEK293T cells expressing the indicated constructs. (G) Immunoblotting analysis of the specific association of PRMT5 and WDR77 with *PDCD1* 3' UTR WT or mutants in HEK293T cells expressing the indicated constructs. (H) Dual-luciferase assay showing relative luciferase activities in PRMT5 KD or WDR77 KD HEK293T cells expressing the indicated constructs. (I) RIP-qPCR analysis of *Pdcd1* 3' UTR enriched by PRMT5 and WDR77 in HEK293T cells expressing the indicated constructs. (J) Immunoblotting analysis of the specific association of PRMT5 and WDR77 with

Pdcd1 3' UTR WT or mutants in HEK293T cells expressing the indicated constructs. For B, F and H-I (n = 3), by one-way ANOVA with Dunnett's test. Data are presented as mean ± SEM or SD. *$p < 0.05$, **$p < 0.01$, ***$p < 0.001$. ns, no significant.

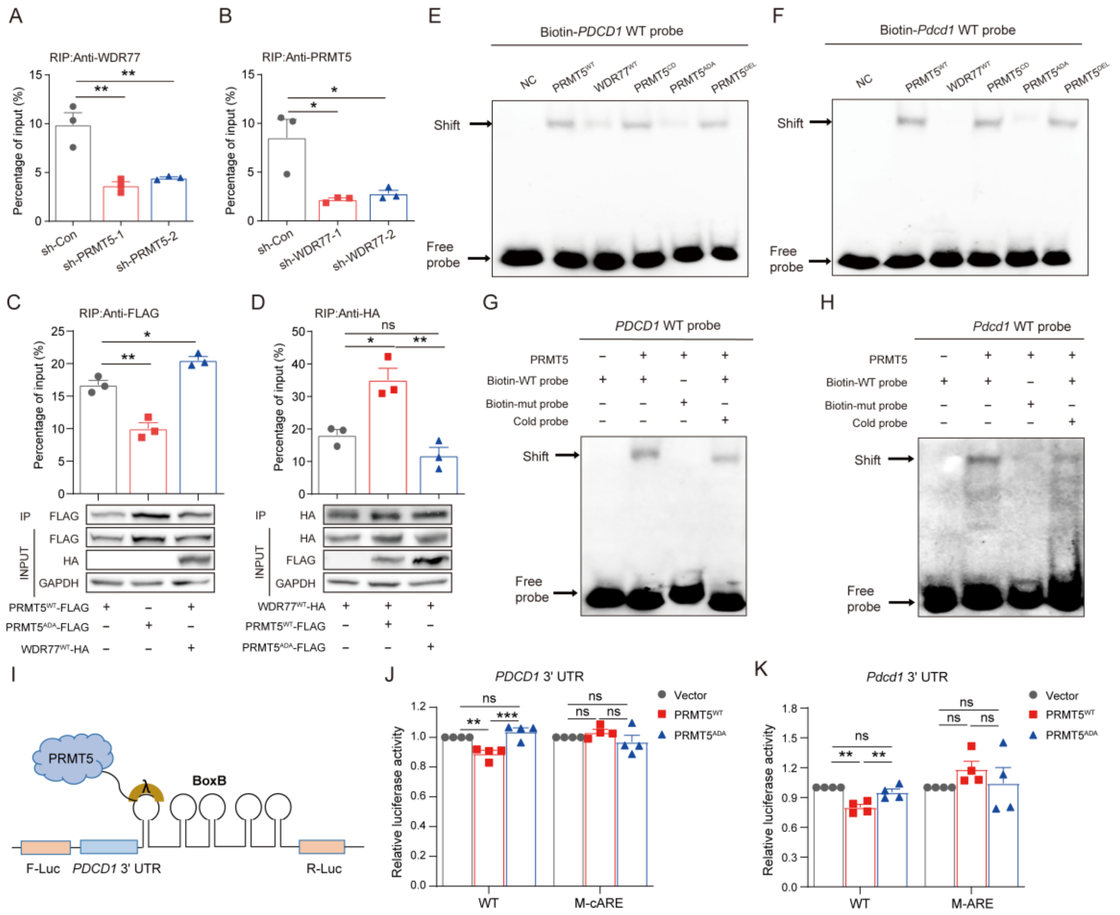

Supplemental Figure 9. PRMT5 binds to *PDCD1* 3' UTR in a WDR77-dependent manner. (A and B) RIP-qPCR analysis of *PDCD1* 3' UTR enriched by WDR77 (A) or PRMT5 (B) in the indicated Jurkat cells. (C and D) RIP-qPCR analysis (top) of *PDCD1* 3' UTR enriched by FLAG and immunoblotting analysis (bottom) of FLAG (C) or HA (D) in Jurkat cells expressing the indicated constructs. (E-F) EMSA analysis of biotinylated *PDCD1* (E) or *Pdcd1* 3' UTR (F) using IVT-WDR77^{WT}, PRMT5^{WT}, PRMT5^{CD}, PRMT5^{ADA}, and PRMT5^{DEL}. (G-H) EMSA analysis of the association of the indicated *PDCD1* (G) or *Pdcd1* (H) 3' UTR probes and IVT-PRMT5^{WT}. (I) Schematic diagram of the tethering reporter assay. (J-K) Dual-luciferase assay showing relative luciferase activities of *PDCD1* (J) or *Pdcd1* (K) 3' UTR WT or mutants in HEK293T cells expressing the indicated constructs. For A-C (n = 3), by one-way ANOVA with Dunnett's test; for D (n = 3) and J-K (n = 4), by one-way ANOVA with Tukey's test. Data are presented as mean ± SEM or SD. *p < 0.05, **p < 0.01, ***p < 0.001. ns, no significant.

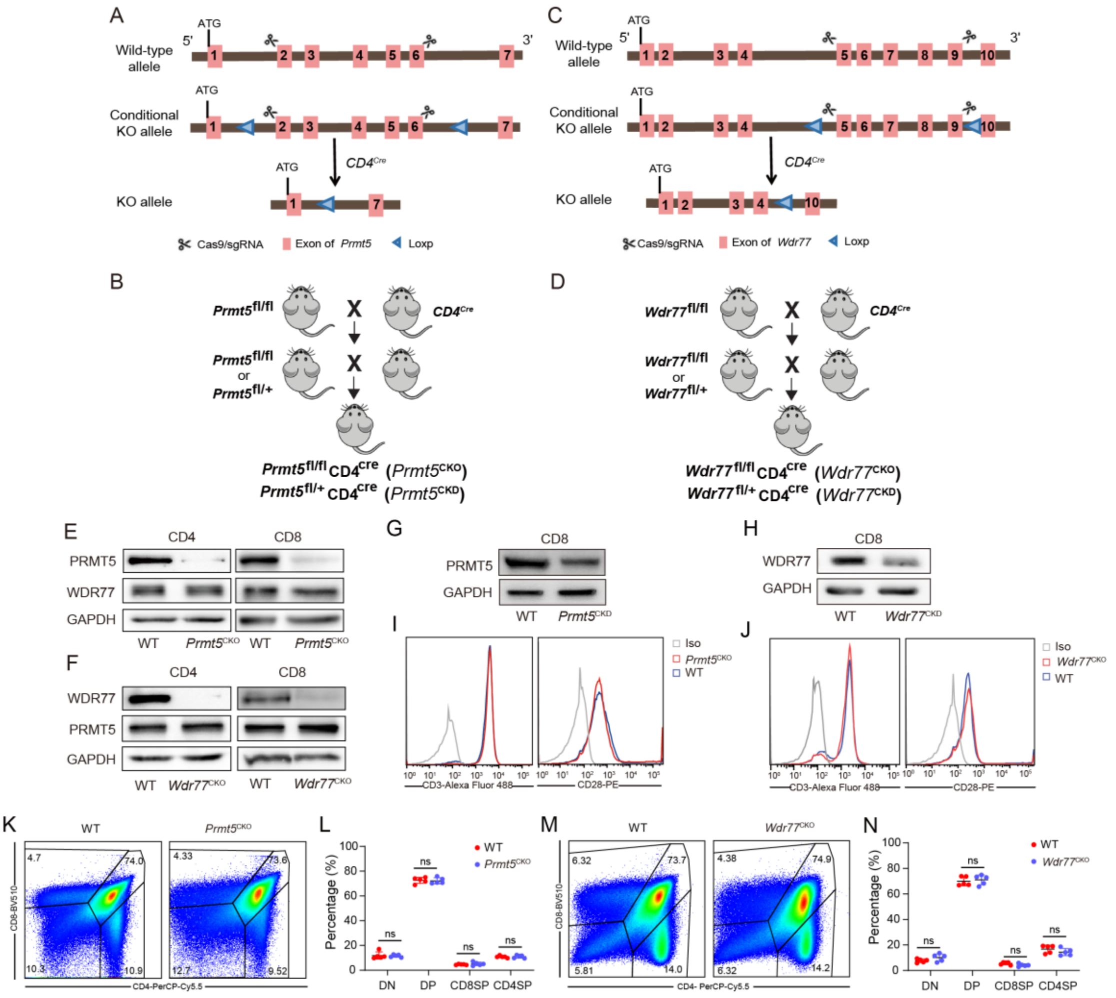

Supplemental Figure 10. PRMT5 and WDR77 do not affect T cell development in the thymus. (A-D) Schematic representation of the gene targeting and mice breeding strategies to generate *Prmt5^{CKO}* or *Prmt5^{CKD}* (A and B) and *Wdr77^{CKO}* or *Wdr77^{CKD}* (C and D) mice. (E-H) Immunoblotting analysis of PRMT5 and WDR77 in CD4$^+$ or CD8$^+$ T cells from the spleens of *Prmt5^{CKO}*/*Prmt5^{CKD}* (E and G) or *Wdr77^{CKO}*/*Wdr77^{CKD}* (F and H) and their WT littermate controls. (I-J) FACS assay showing cell surface levels of CD3 and CD28 in CD8$^+$ T cells from *Prmt5^{CKO}* (I) or *Wdr77^{CKO}* (J) and their respective WT littermate controls. (K-N) Representative plots of CD4 and CD8 expression (K and M) and percentages of CD4$^-$CD8$^-$ double-negative (DN), CD4$^+$CD8$^+$ double-positive (DP), CD4$^+$ single-positive (CD4SP), and CD8$^+$ single-positive (CD8SP) cells (L and N) out of the total thymocytes from *Prmt5^{CKO}* (L) or

Wdr77^{CKO} (N) and their respective WT littermate controls. For L and N (n = 5), by unpaired two-tailed Student's t test. Data are presented as mean ± SEM or SD. ns, no significant.

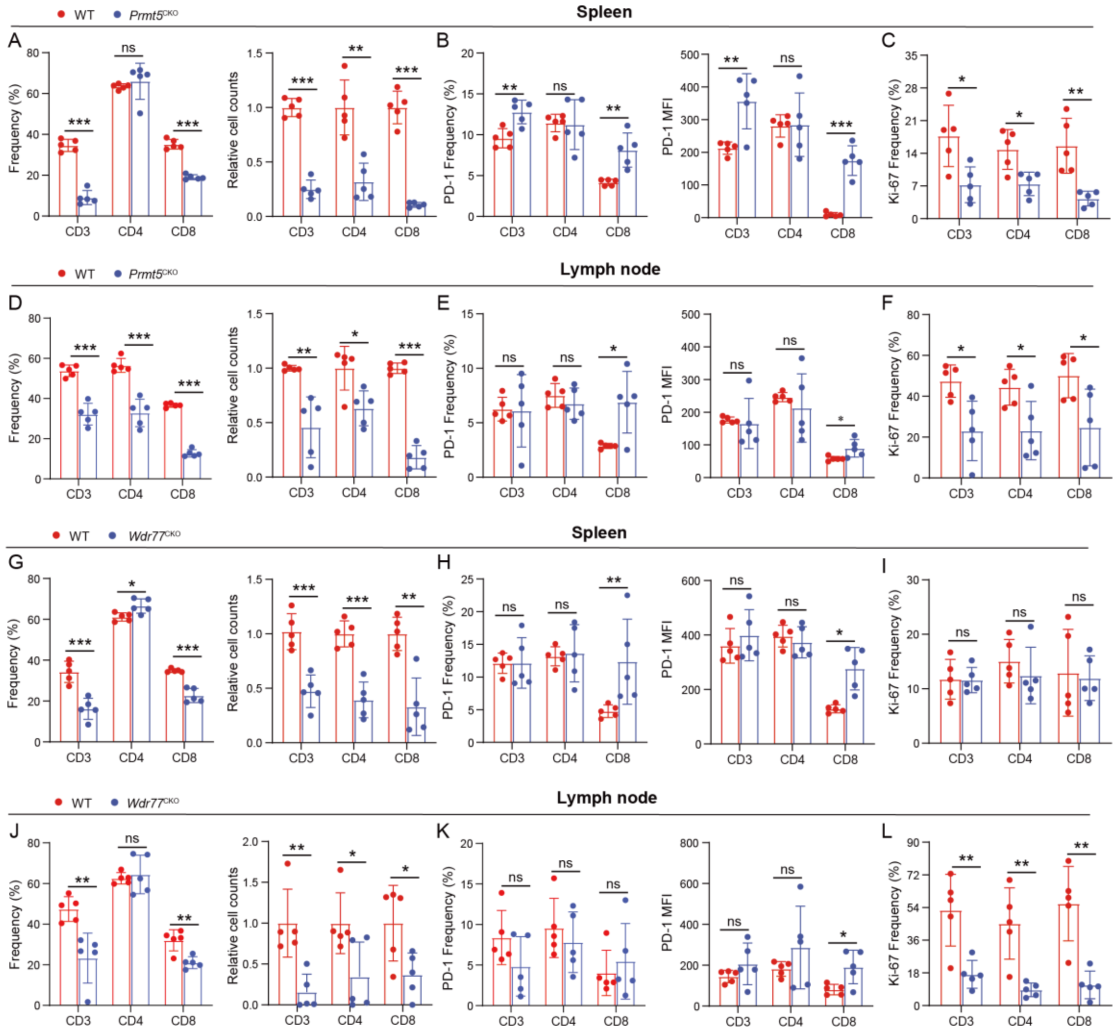

Supplemental Figure 11. T cell-specific *Prmt5* or *Wdr77* deficiency dampens peripheral T cell homeostasis. (A-L) FACS assays showing frequencies (left) and relative cell counts (right) of $CD3^+$ and their $CD4^+$ and $CD8^+$ subsets (A, D, G and J), percentages of positive cells (left) and relative MFI levels (right) of Pd-1 (B, E, H and K) and percentages of Ki-67$^+$ cells (C, F, I and L) in $CD3^+$, $CD4^+$ and $CD8^+$ T cells in the spleen and lymph nodes of *Prmt5^{CKO}* or *WDR77^{CKO}* and their WT controls. For A-L (n = 5), by unpaired two-tailed Student's t test. Data are presented as mean ± SEM or SD. *p < 0.05, **p < 0.01, ***p < 0.001. ns, no significant.

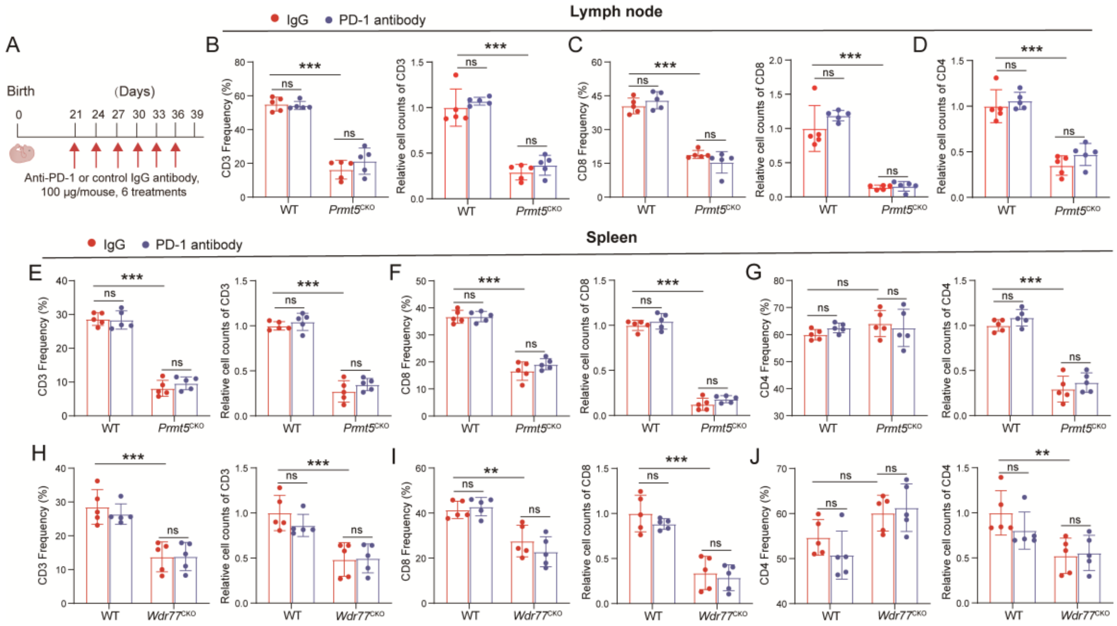

Supplemental Figure 12. PRMT5 and WDR77 regulate peripheral T cell homeostasis independently of the PD-1 pathway. (A) Schematic representation of the anti-PD-1 treatment plan for 3-week-old *Prmt5*^{CKO} or *WDR77*^{CKO} mice and their WT controls. (B-J) Frequencies (left) and relative cell counts (right) of CD3^{+} (B, E and H), their CD8^{+} subsets (C, F and I) and CD4^{+} subsets (D, G and J) in the spleen or lymph nodes of *Prmt5*^{CKO} or *WDR77*^{CKO} and their WT controls treated with IgG or anti-PD-1 antibody. For B-J (n = 5), by two-way ANOVA. Data are presented as mean ± SEM or SD. $^{**}p < 0.01$, $^{***}p < 0.001$. ns, no significant.

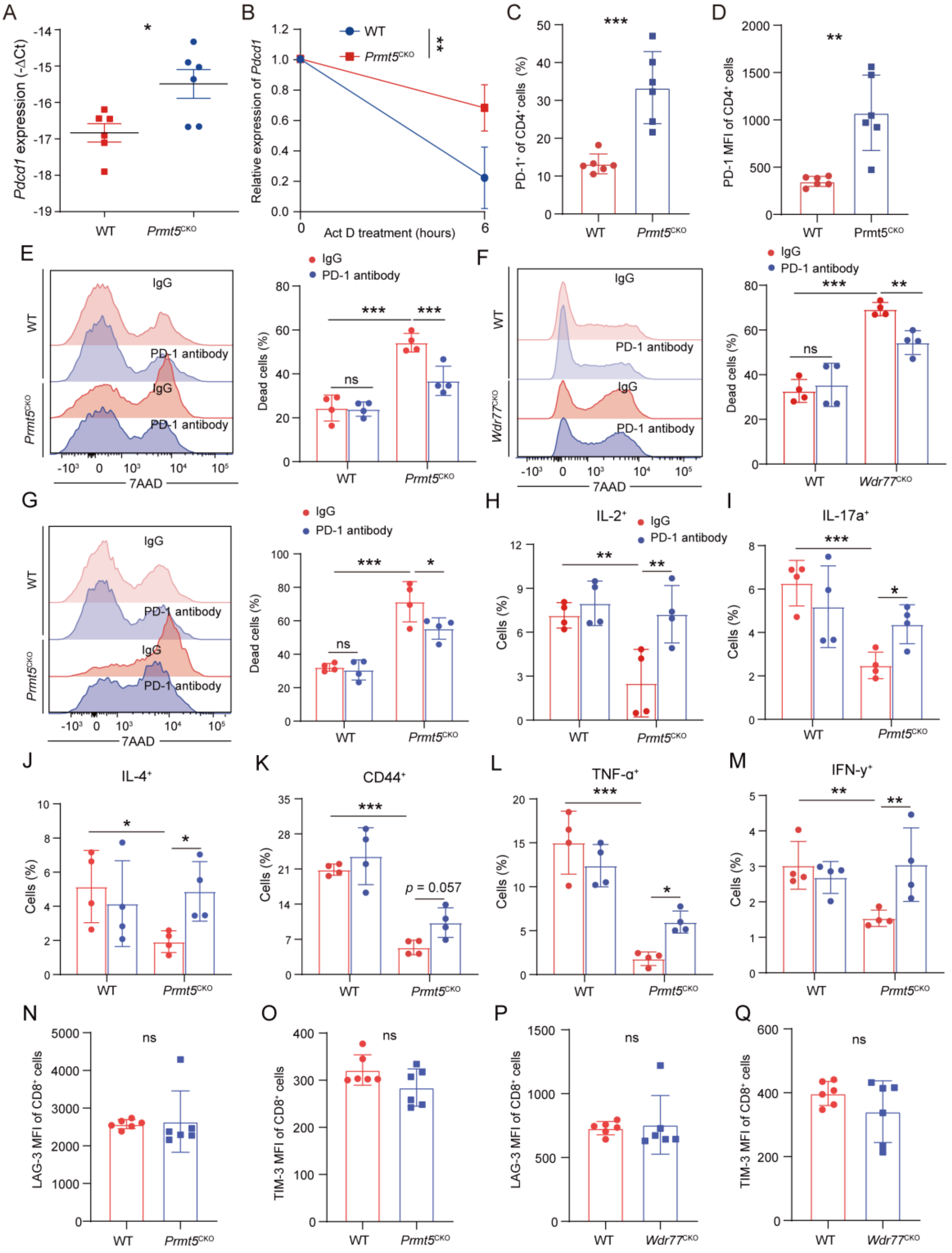

Supplemental Figure 13. PRMT5 and WDR77 maintain T cell effector function through PD-1. (A-B) mRNA levels (A) and half-lives of *Pdcd1* (B) in activated CD4⁺ T cells from *Prmt5*^{CKO} and WT mice. (C-D) Percentages of positive cells (C) and MFI levels (D) of PD-1 in activated CD4⁺ T cells from *Prmt5*^{CKO} and WT mice. (E-G) Cell death of activated CD8⁺ (E and F) or CD4⁺

T (G) cells treated with anti-PD-1 from *Prmt5*^{CKO} or *Wdr77*^{CKO} and WT mice. (H-M) Effector molecules production of the activated CD4$^+$ T cells treated with anti-PD-1 antibody from *Prmt5*^{CKO} mice and their WT mice, measured by IL-2, IL-17α, IL-4, CD44, TNF-α and IFN-γ. (N-Q) MFI levels of LAG-3 (N and P) and TIM-3 (O and Q) in activated CD8$^+$ T cells from *Prmt5*^{CKO} or *Wdr77*^{CKO} and their WT controls. For A-D and N-Q (n = 6), by unpaired two-tailed Student's t test; for E-M (n = 4), by two-way ANOVA. Data are presented as mean ± SEM or SD. *$p < 0.05$, **$p < 0.01$, ***$p < 0.001$. ns, no significant.

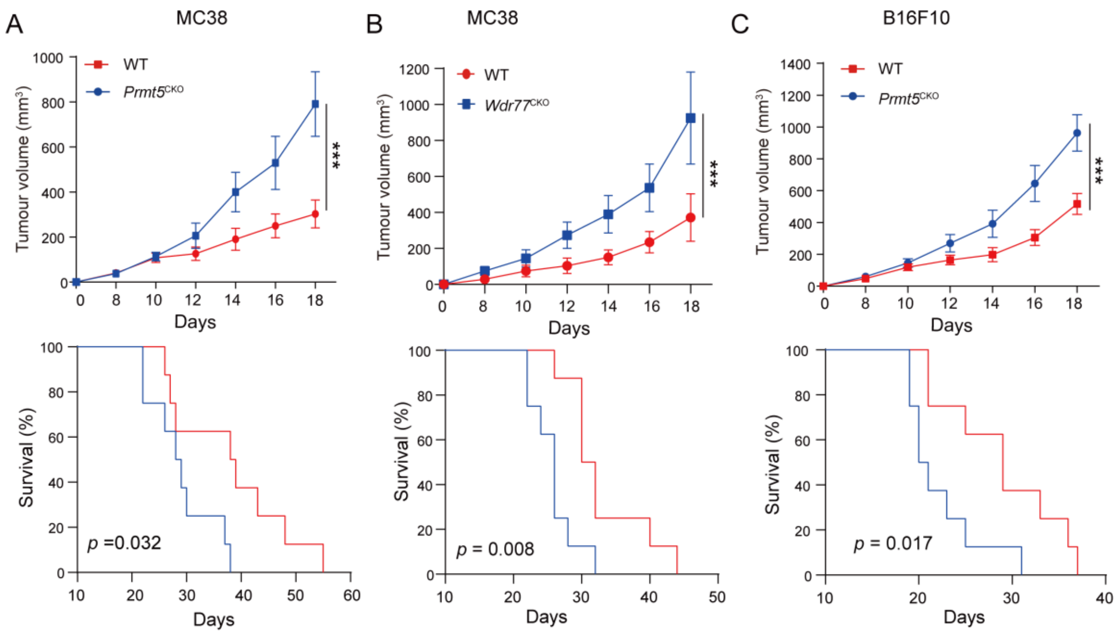

Supplemental Figure 14. T cell-specific *Prmt5* or *Wdr77* deficiency enhances tumor progression. (A-C) Tumor growth curves (top) and survival rates (bottom) of MC38 colorectal carcinoma (A and B) and B16F10 melanomas (C) in *Prmt5*^{CKO} or *Wdr77*^{CKO} and their WT mice. For A-C (n =8), by two-way ANOVA. Data are presented as mean ± SEM or SD. ***p < 0.001.

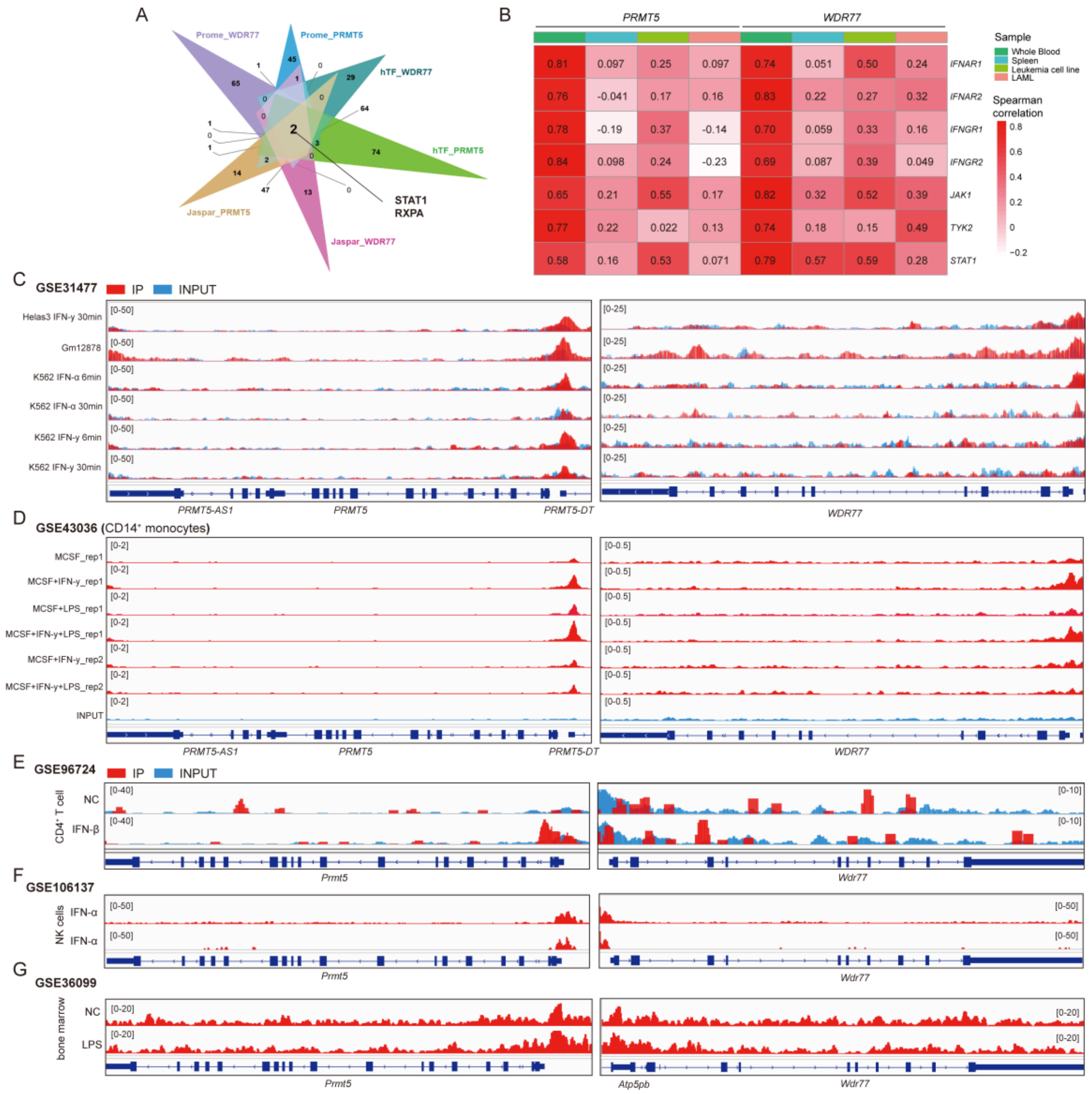

Supplemental Figure 15. STAT1 is a potential transcription factor for *PRMT5* and *WDR77*. (A) Prediction of common transcription factors for *PRMT5* and *WDR77*. (B) Correlation analysis between *PRMT5* or *WDR77* and related genes in the IFN-JAK/STAT1 signaling pathway. (C-G) ChIP-seq datasets for STAT1 displayed in the promoters of *PRMT5* (left) and *WDR77* (right) in human (C and D) and mouse (E-G).

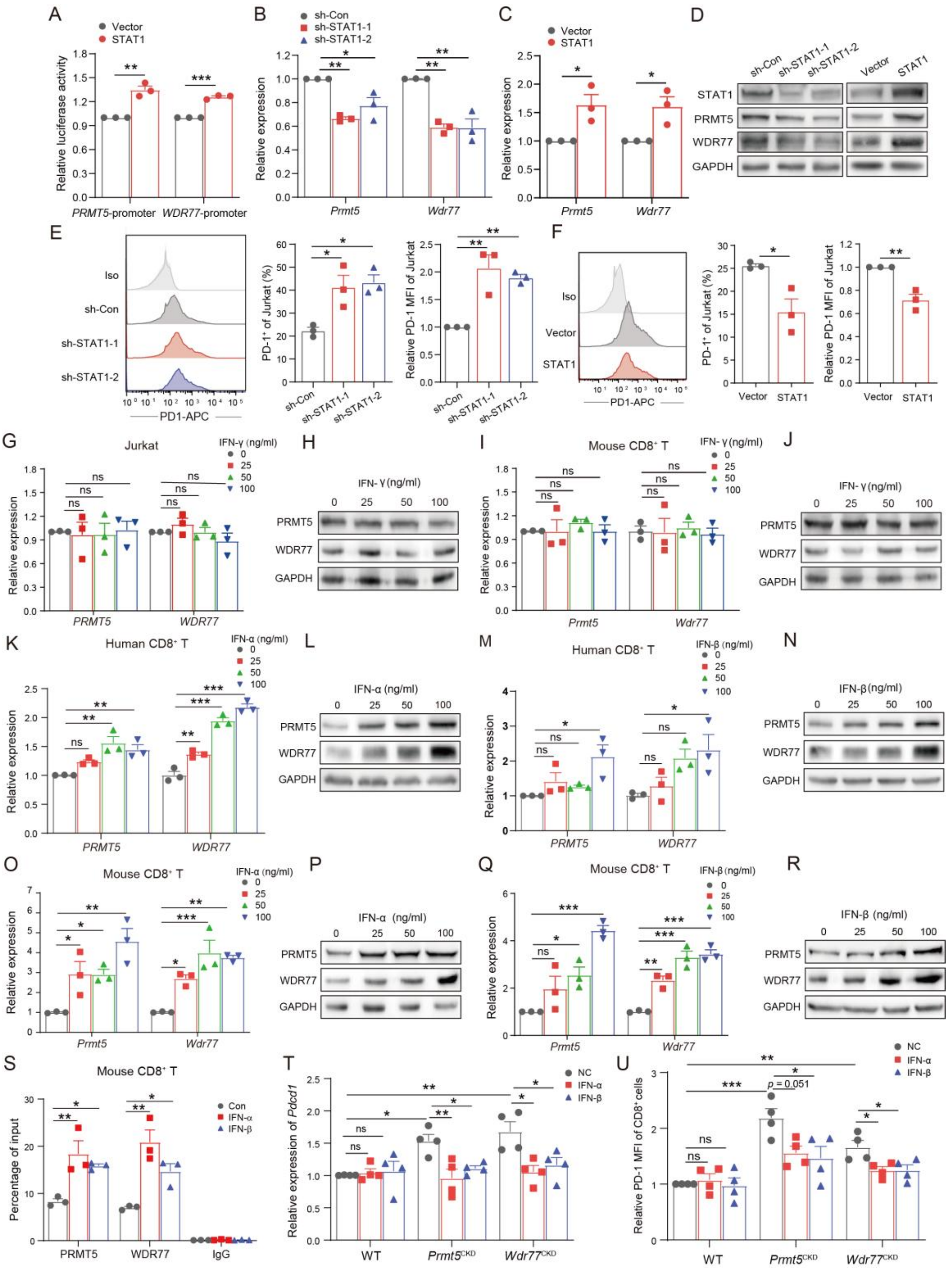

Supplemental Figure 16. The type I IFN/STAT1-PRMT5/WDR77 axis inhibits PD-1 expression. (A) Dual-luciferase assay showing relative luciferase activities of the promoters of *PRMT5* or *WDR77* in HEK293T cells expressing STAT1. (B-D) qPCR analysis (B and C) and immunoblotting analysis (D) of PRMT5 and WDR77 in STAT1 KD (left) and OE (right) EL4 cells.

(E-F) Representative MFI plots (left), percentages of positive cells (middle) and relative MFI levels (right) of PD-1 in STAT1 KD (E) and OE (F) EL4 cells. (G-R) mRNA levels (G, I, K, M, O and Q) and protein levels (H, J, L, N, P and R) of PRMT5 and WDR77 in Jurkat and human or mouse $CD8^+$ T cells treated with IFN-γ, IFN-α or IFN-β. (S) RIP-qPCR analysis of *Pdcd*1 3' UTR enriched by PRMT5 and WDR77 in mouse $CD8^+$ cells treated with IFN-α or IFN-β. (T and U) mRNA levels (T) and relative MFI levels (U) of PD-1 in activated $CD8^+$ T cells treated with IFN-α or IFN-β from $Prmt5^{CKD}$ or $Wdr77^{CKD}$ mice. For A, C and F (n = 3), by unpaired two-tailed Student's t test; For B, E, G, I, K, M, O, Q and S (n = 3), for T-U (n = 4), by one-way ANOVA with Dunnett's test. Data are presented as mean ± SEM or SD. *$p < 0.05$, **$p < 0.01$, ***$p < 0.001$. ns, no significant.

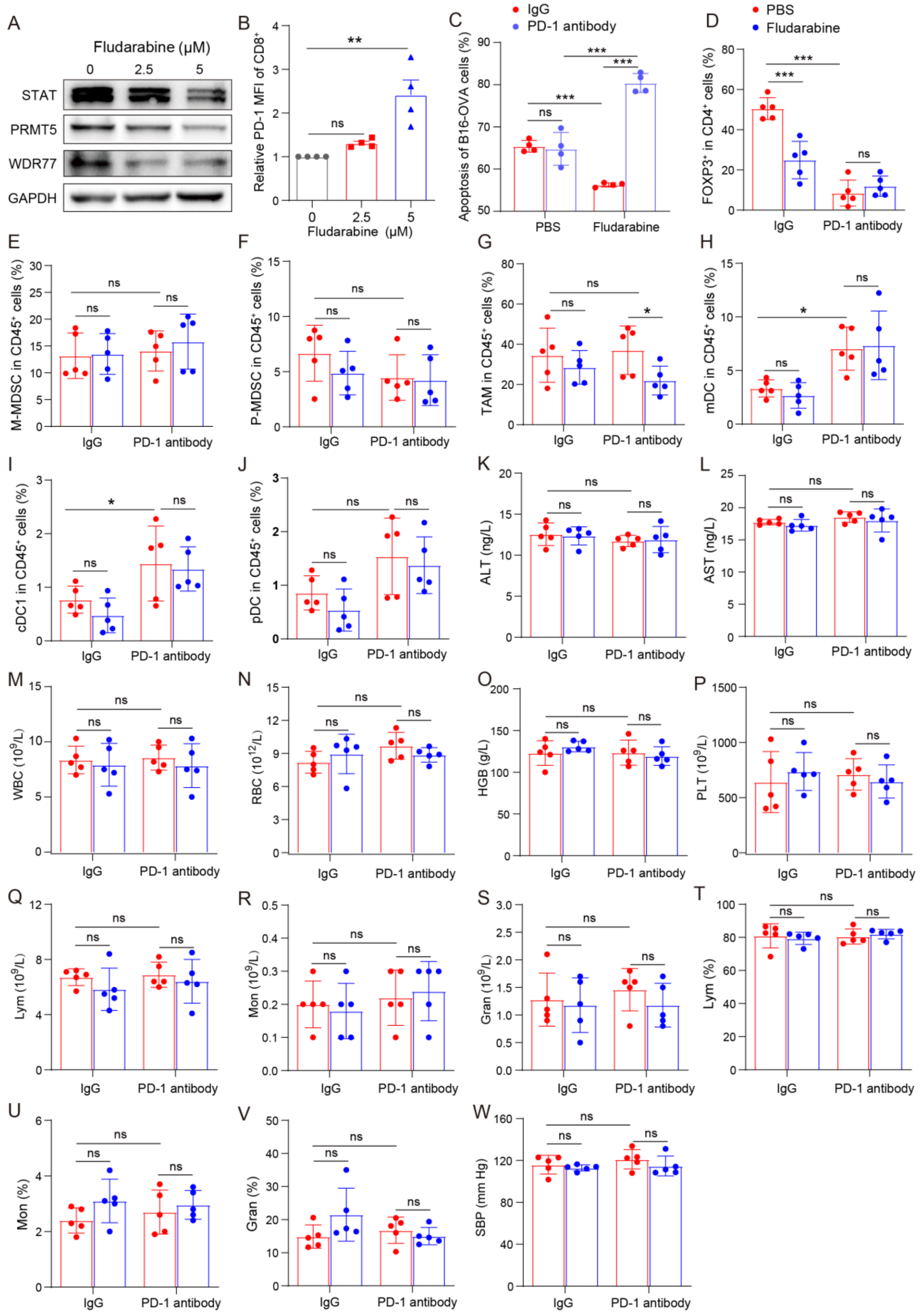

Supplemental Figure 17. Fludarabine in combination with PD-1 antibody therapy may represent a feasible therapeutic strategy. (A) Immunoblotting analysis of STAT1, PRMT5 and WDR77 expression in mouse CD8⁺ T cells

treated with fludarabine. (B) Relative MFI levels of PD-1 in mouse $CD8^+$ T cells treated with fludarabine. (C) Quantification of *in vitro* killing of B16-OVA cells by OT-1 $CD8^+$ T cells with or without fludarabine or anti-PD-1 treatment. (D-J) Flow cytometry analysis showing the percentage of Treg ($CD4^+FOXP3^+$) (D), M-MDSC ($CD45^+CD11b^+Ly6G^-Ly6C^+$) (E), P-MDSC ($CD45^+CD11b^+Ly6G^+Ly6C^-$) (F), TAM ($CD45^+CD11b^+F4/80^+$) (G), mDC ($CD45^+CD11c^+F4/80^-MHC\text{-}II^+$) (H), cDC1 ($CD45^+CD11b^-CD11c^+$ $F4/80^-MHC\text{-}II^+XCR1^+$) (I) and pDC ($CD45^+CD11b^-$ $CD11c^+$ $F4/80^-MHC\text{-}II^+B220^+$) (J) in the $CD45^+$ cell population of subcutaneous MC38 tumors from mice treated with fludarabine and anti-PD-1. (K-L) The content of Alanine aminotransferase (ALT) (K) and aspartate aminotransferase (AST) (L) from mice treated with fludarabine and anti-PD-1. (M-V) The content or percentage of white blood cell (WBC) (M), red blood cell (RBC) (N), hemoglobin (HGB) (O), Platelet (PLT) (P), Lymphocyte (Lym) (Q and T), Monocyte (Mon) (R and U) and Granulocyte (Gran) (S and V) from mice treated with fludarabine and anti-PD-1. (W) The systolic blood pressure (SBP) from mice treated with fludarabine and anti-PD-1. For B (n = 4 per group), by one-way ANOVA with Dunnett's test; For C (n = 4 per group), for D-W (n = 5 per group), by two-way ANOVA. Data are presented as mean ± SEM or SD. *$p < 0.05$, **$p < 0.01$, ***$p < 0.001$. ns, no significant.

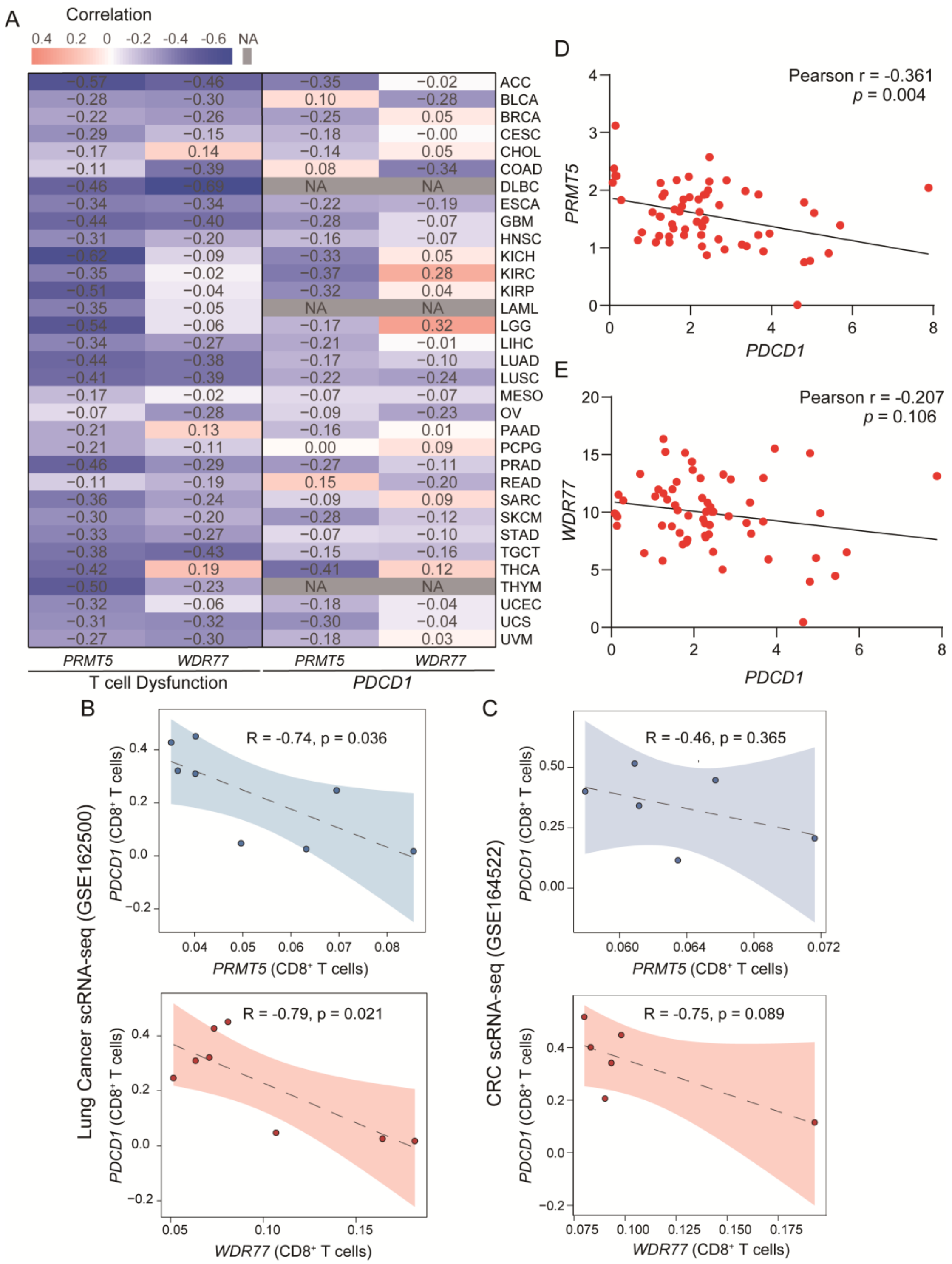

Supplemental Figure 18. ***PRMT5*** **and** ***WDR77*** **are associated with** ***PDCD1.***
(A) Correlation analysis of the expression levels between *PRMT5* or *WDR77* and T cell dysfunction or *PDCD1* in 33 tumor types from TCGA datasets. (B and C) Correlation analysis between *PRMT5* (top) or *WDR77* (down) expression and *PDCD1* in infiltrating CD8$^+$ T cells with lung cancer (B, GSE162500) and

colorectal cancer (C, GSE164522). (D-E) Correlation analysis of the expression levels between *PRMT5* (D) or *WDR77* (E) and *PDCD1* across multiple combinations of T cell subsets from 10K Immunomes dataset. For B-E, by Pearson correlation analysis.

Supplementary Table 3 Target sequences of shRNA and siRNA for KD.

Targets	Target sequences (5'-3')
sh-PRMT5-1	GGTGAACACAGTACTACATGG
sh-PRMT5-2	GGAATCTCAGACATATGAAGT
sh-WDR77-1	GGGAACTAGATGAGAATGA
sh-WDR77-2	GTGGACACCAAGAGTACAA
sh-human STAT1-1	GAACAGAAATACACCTACGAA
sh-human STAT1-2	CCCTGAAGTATCTGTATCCAA
sh-mouse STAT1-1	GGAAATCAAGACCCTAGAAGA
sh-mouse STAT1-2	GCCTCTCATTGTCACCGAAGA
sh-AGO2-1	GGTCTAAAGGTGGAGATAACG
sh-AGO2-2	GCACCTGAAGAACACGTATGC
si-PDCD1-1	CCACAGGACTCATGTCTCA
si-PDCD1-2	CAGGCCTAGAGAAGTTTCA

Supplementary Table 4 List of primary antibodies.

Antibodies	Manufacturer	Application
PRMT5	Thermo Fisher Scientific, #A300-849A	1:2000 for WB, 2μg for RIP
WDR77 for human	Thermo Fisher Scientific, #A301-562A	1:2000 for WB, 2μg for RIP
WDR77 for mouse	Abcam, #ab154190	1:2000 for WB
STAT1	Proteintech, #10144-2-AP	1:2000 for WB, 2μg for ChIP
IGF2BP3	Proteintech, #15073-1-AP	1:2000 for WB
SMC2	Abcam, #ab10399	1:2000 for WB
AGO2	Selleck, #F0519	1:2000 for WB，2 μg for IP
FLAG	Proteintech, #66008-4-Ig	1:3000 for WB, 2 μg for RIP
GAPDH	Proteintech, #10494-1-AP	1:4000 for WB
p53	Proteintech, # 10442-1-AP	1:2000 for WB
HA	Cell Signaling Technology, #3724	1:4000 for WB
ERK	Cell Signaling Technology, #4695	1:1000 for WB
pERK	Cell Signaling Technology, #4370	1:1000 for WB
AKT	Cell Signaling Technology, #9272	1:1000 for WB
pAKT	Cell Signaling Technology, #4060	1:1000 for WB
S6	Cell Signaling Technology, #2317	1:1000 for WB
pS6	Cell Signaling Technology, #4858	1:1000 for WB
SDMA	Cell Signaling Technology, #13222	1:1000 for WB
HRP-conjugated goat anti-rabbit IgG	Beyotime, #A0208	1: 4000 for WB
HRP-conjugated goatigg anti-mouse IgG	Beyotime, #A0216	1: 4000 for WB
HRP-conjugated Mouse anti-Rabbit IgG Light Chain	ABclonal, #AS061	1: 2000 for WB
HRP-conjugated goat anti-mouse IgG light chain	ABclonal, #AS062	1: 2000 for WB

Supplementary Table 5 List of qPCR primers.

	**Forwards (5'-3')**	**Reverses (5'-3')**
PRMT5	GAAAACGTGGATGTGGTGGC	CTAGTGGGGAGAATGGCTGC
WDR77	TAGAAGCCAAGCCCACAGAG	AGTAACACTTGCAGGTCCAGG
GAPDH	GGAGCGAGATCCCTCCAAAAT	GGCTGTTGTCATACTTCTCATGG
18S	CGGACAGGATTGACAGATTGATAGC	TGCCAGAGTCTCGTTCGTTATCG
F-LUC	TGCTGGTGCCCACACTATTT	CGTGCAAGTTGCTTAGGTCG
R-LUC	GGGCGAGAAAATGGTGCTTG	GCCCTTCTCCTTGAATGGCT
PDCD1	TCGTGCTAAACTGGTACCGC	CTGACCACGCTCATGTGGAA
Prmt5	AACGAGCTGTCACCTGAGTG	GGAAGTTGTGCAACCGAACC
Wdr77	GCCTCTCCCCACAAAGACTC	GGTGTCCACAAGGGAGACAG
Pdcd1	GTCCCAGCAACCAGACTGAA	GCCGTGTGTCAAGGATGTTC

Supplementary Table 6 List of RIP-qPCR primers.

	Forwards (5'-3')	Reverses (5'-3')
PDCD1 3' UTR	TCCTCACATCCACACACTGC	TAGAACCACAGGGAAGGGGG
Pdcd1 3' UTR	GTCCTGGAGTTGGGTTCTGG	AAGCCCTGGTCATTTTGGGT

Supplementary Table 7 List of ChIP-qPCR primers.

	Forwards (5'-3')	Reverses (5'-3')
PRMT5 promoter	GTCCCCAACGAGCTATGGTG	GCTGGCAGAACTTAGGGACC
WDR77 promoter	GAAAGTACCACCCGGGACAG	ACCGCAACTCTACTTCTCGC

Supplementary Table 8 Sequences of gRNAs for CARPID.

Targets	gRNAs-F	gRNAs-R
5' UTR-P-1	AAACGCCTTCTCCACTGCTCAGGCGGAGGTGAGC	CTTGGCTCACCTCCGCCTGAGCAGTGGAGAAGGC
5' UTR-P-2	AAACGACTGGCCAGGGCGCCTGTGGGATCTGCAT	CTTGATGCAGATCCCACAGGCGCCCTGGCCAGTC
CDS-P1-1	AAACGTACCAGTTTAGCACGAAGCTCTCCGATGT	CTTGACATCGGAGAGCTTCGTGCTAAACTGGTAC
CDS-P1-2	AAACGGCTGGCTGCGGTCCTCGGGGAAGGCGGCC	CTTGGGCCGCCTTCCCCGAGGACCGCAGCCAGCC
CDS-P2-1	AAACGAAAGACAATGGTGGCATACTCCGTCTGCT	CTTGAGCAGACGGAGTATGCCACCATTGTCTTTC
CDS-P2-2	AAACGCACTCCGAGGGCCGTCAGCTGAGCCCCTG	CTTGCAGGGGCTCAGCTGACGGCCCTCGGAGTGC
3' UTR-P1-1	AAACGTGGGCATTGAGACATGAGTCCTGTGGTGG	CTTGCCACCACAGGACTCATGTCTCAATGCCCAC
3' UTR-P1-2	AAACGACCTGAAGCAGTGACTGCATCTGGCCCTC	CTTGGAGGGCCAGATGCAGTCACTGCTTCAGGTC
3' UTR-P2-1	AAACGATGTGAGGAGTGGATAGGCCACGGCGGGG	CTTGCCCCGCCGTGGCCTATCCACTCCTCACATC
3' UTR-P2-2	AAACGGATCCCTTGTCCCAGCCACTCAGGTGCCT	CTTGAGGCACCTGAGTGGCTGGGACAAGGGATCC

Supplementary Table 9 Sequences of probes for EMSA.

Probes	Sequence (5’-3’)
Biotin-*PDCD1* WT	UaUUaUaUUaUaaUUaUaaUUaaaU-3`Biotin
PDCD1 WT	UaUUaUaUUaUaaUUaUaaUUaaaU
Biotin-*PDCD1* mut	UaUUaUaGUaUaaGUaUaaGUaaaU-3`Biotin
Biotin-*Pdcd1* WT	agcccgUUaUaaUUaaaUggUacca-3`Biotin
Pdcd1 WT	agcccgUUaUaaUUaaaUggUacca
Biotin-*Pdcd1* mut	agcccgGUaUaaGUaaaGggUacca-3`Biotin

Supplementary Table 10 List of fluorescent antibodies for FACS.

Antibodies	Manufacturer
Brilliant Violet 605™ anti-mouse CD45	Biolegend, #103140
Brilliant Violet 510™ anti-mouse CD8a	Biolegend, #100752
PerCP/Cyanine5.5 anti-mouse CD4	Biolegend, #100434
Alexa Fluor® 700 anti-mouse CD4	Biolegend, #100430
Alexa Fluor® 488 anti-mouse CD3ε	Biolegend, #152322
Brilliant Violet 650™ anti-mouse CD3	Biolegend, #100229
APC anti-mouse CD279	Biolegend, #109112
PE anti-mouse CD28	Biolegend, #102106
Brilliant Violet 421™ anti-mouse/human Ki-67	Biolegend, #151208
APC anti-mouse TNF-α	Biolegend, #506308
FITC anti-mouse TNF-α	Biolegend, #506304
Brilliant Violet 421™ anti-mouse IL-2	Biolegend, #503826
Brilliant Violet 605™ anti-mouse Ki-67	Biolegend, #652413
FITC anti-mouse IFN-γ	Biolegend, #505806
APC/Cyanine7 anti-mouse IFN-γ	Biolegend, #505850
PE anti-mouse/human CD44	Biolegend, #103008
PerCP/Cyanine5.5 anti-mouse/human CD44	Biolegend, #103031
PE anti-mouse IL-4	Biolegend, #504104
Brilliant Violet 421™ anti-mouse IL-17A	Biolegend, # 506925
PE anti-human/mouse Granzyme B Recombinant	Biolegend, #372208
PerCP/Cyanine5.5 anti-human/mouse Granzyme B	Biolegend, #372212
PE anti-mouse CD366 (Tim-3)	Biolegend, #134004
APC anti-mouse CD223 (LAG-3)	Biolegend, #125210
PE anti-mouse/human CD11b	Biolegend, #101208
APC/Cyanine7 anti-mouse Ly-6G/Ly-6C (Gr-1)	Biolegend, #108423
Spark Red™ 718 anti-mouse CD11c	Biolegend, #117371
APC anti-mouse Ly-6C	Biolegend, #128016
FITC anti-mouse Ly-6G	Biolegend, #127606
PE/Cyanine7 anti-mouse/human CD45R/B220	Biolegend, #103222
Brilliant Violet 421™ anti-mouse F4/80	Biolegend, #123137
Brilliant Violet 510™ anti-mouse/rat XCR1	Biolegend, #148218
PerCP/Cyanine5.5 anti-mouse I-A/I-E	Biolegend, #107626
APC Rat IgG2b, κ Isotype Ctrl	Biolegend, #400611
Brilliant Violet 421™ Rat IgG2b, κ Isotype Ctrl	Biolegend, #400639
APC Rat IgG1, κ Isotype Ctrl	Biolegend, #400411
FITC Rat IgG1, κ Isotype Ctrl	Biolegend, #400405
PE Rat IgG2b, κ Isotype Ctrl	Biolegend, #400607
PerCP/Cyanine5.5 Rat IgG2b, κ Isotype Ctrl	Biolegend, #400631
PE Rat IgG1, κ Isotype Ctrl	Biolegend, #400407
Brilliant Violet 421™ Rat IgG1, κ Isotype Ctrl	Biolegend, #400429
PE Mouse IgG1, κ Isotype Ctrl	Biolegend, #400111

PerCP/Cyanine5.5 Mouse IgG1, κ Isotype Ctrl	Biolegend, #400149
Brilliant Violet 605™ Rat IgG2a, κ Isotype Ctrl	Biolegend, #400539
APC/Cyanine7 Rat IgG1, κ Isotype Ctrl	Biolegend, # 400422
PRMT5 (A-11) Alexa Fluor 488	SantaCruz, #sc-376937
normal mouse IgG1 Alexa Fluor® 488	SantaCruz, # sc-3890

References

1 Pan, Y. *et al.* Epitranscriptic regulation of HRAS by N(6)-methyladenosine drives tumor progression. *Proceedings of the National Academy of Sciences of the United States of America* **120**, e2302291120, doi:10.1073/pnas.2302291120 (2023).

2 Livak, K. J. & Schmittgen, T. D. Analysis of relative gene expression data using real-time quantitative PCR and the 2(-Delta Delta C(T)) Method. *Methods (San Diego, Calif.)* **25**, 402-408, doi:10.1006/meth.2001.1262 (2001).

3 Zhu, Y. *et al.* DMDRMR promotes angiogenesis via antagonizing DAB2IP in clear cell renal cell carcinoma. *Cell death & disease* **13**, 456, doi:10.1038/s41419-022-04898-3 (2022).

4 Yi, W. *et al.* CRISPR-assisted detection of RNA-protein interactions in living cells. *Nature methods* **17**, 685-688, doi:10.1038/s41592-020-0866-0 (2020).

5 Queiroz, R. M. L. *et al.* Comprehensive identification of RNA-protein interactions in any organism using orthogonal organic phase separation (OOPS). *Nature biotechnology* **37**, 169-178, doi:10.1038/s41587-018-0001-2 (2019).
