# Supplementary material for: Posttranscriptional regulation of PD-1 by PRMT5/WDR77 complex shapes T cell effector function and antitumor immunity
Source: J Clin Invest. 2026 Feb 2;136(3):e191469. doi: 10.1172/JCI191469 (PMC12867141; doi:10.1172/JCI191469)

Figure 1

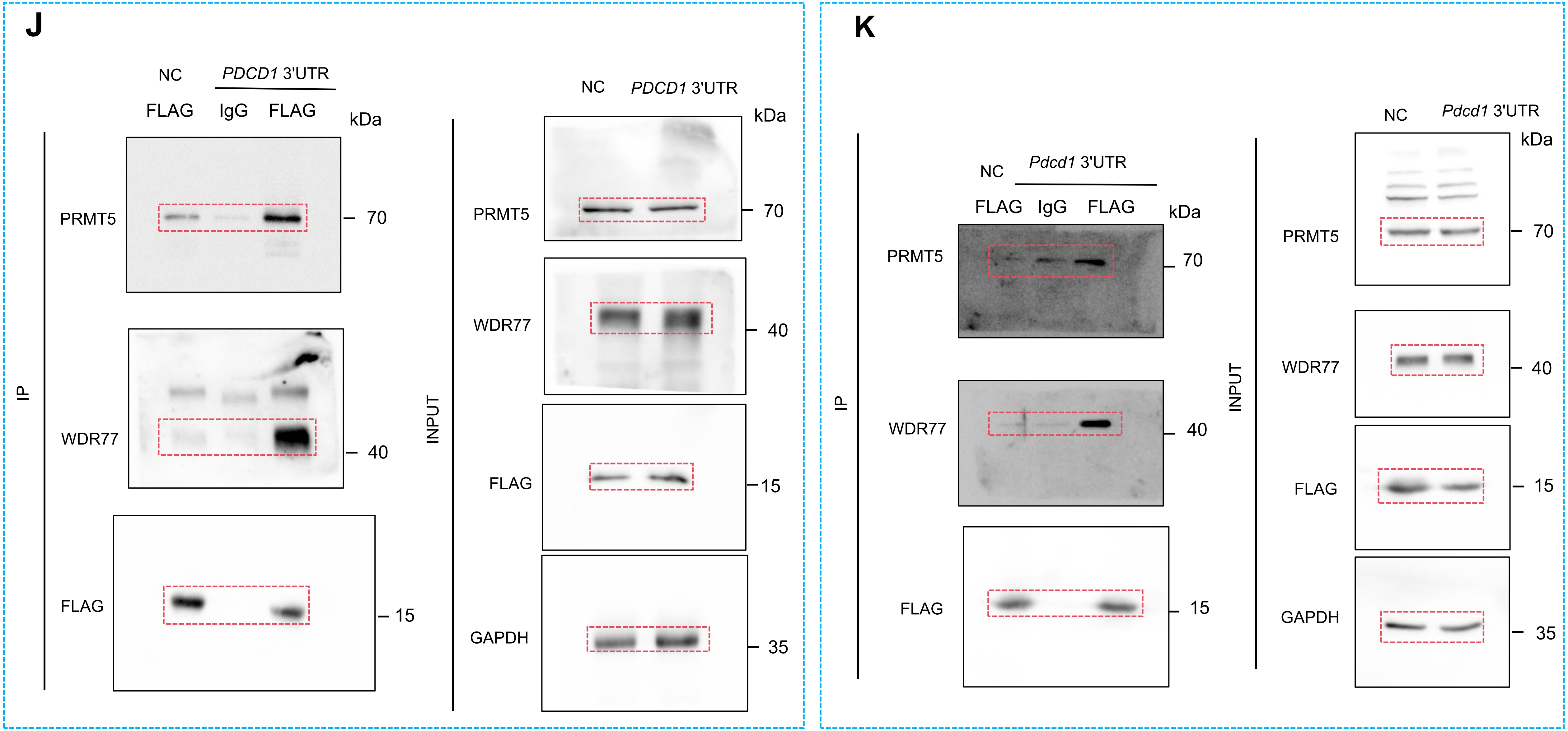

J
NC
PDCD1 3'UTR
FLAG
IgG
FLAG
kDa
IP
PRMT5
70
WDR77
40
FLAG
15
INPUT
NC
PDCD1 3'UTR
kDa
PRMT5
70
WDR77
40
FLAG
15
GAPDH
35
K
IP
NC
Pdcd1 3'UTR
FLAG
IgG
FLAG
kDa
PRMT5
70
WDR77
40
FLAG
15
INPUT
NC
Pdcd1 3'UTR
kDa
PRMT5
70
WDR77
40
FLAG
15
GAPDH
35

Figure 1

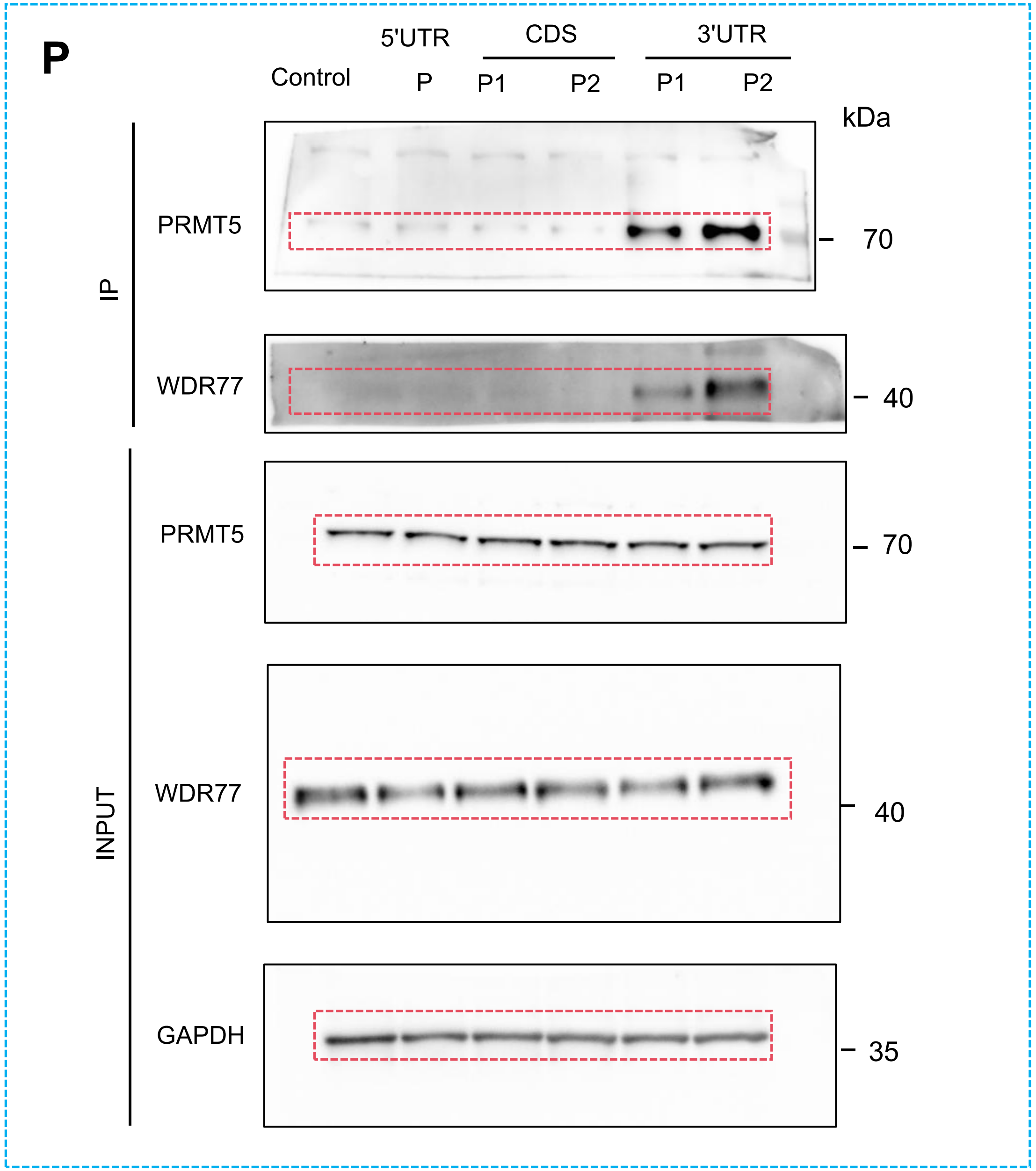

P
5'UTR
CDS
3'UTR
Control
P
P1
P2
P1
P2
kDa
IP
PRMT5
70
WDR77
40
INPUT
PRMT5
70
WDR77
40
GAPDH
35

Figure 2

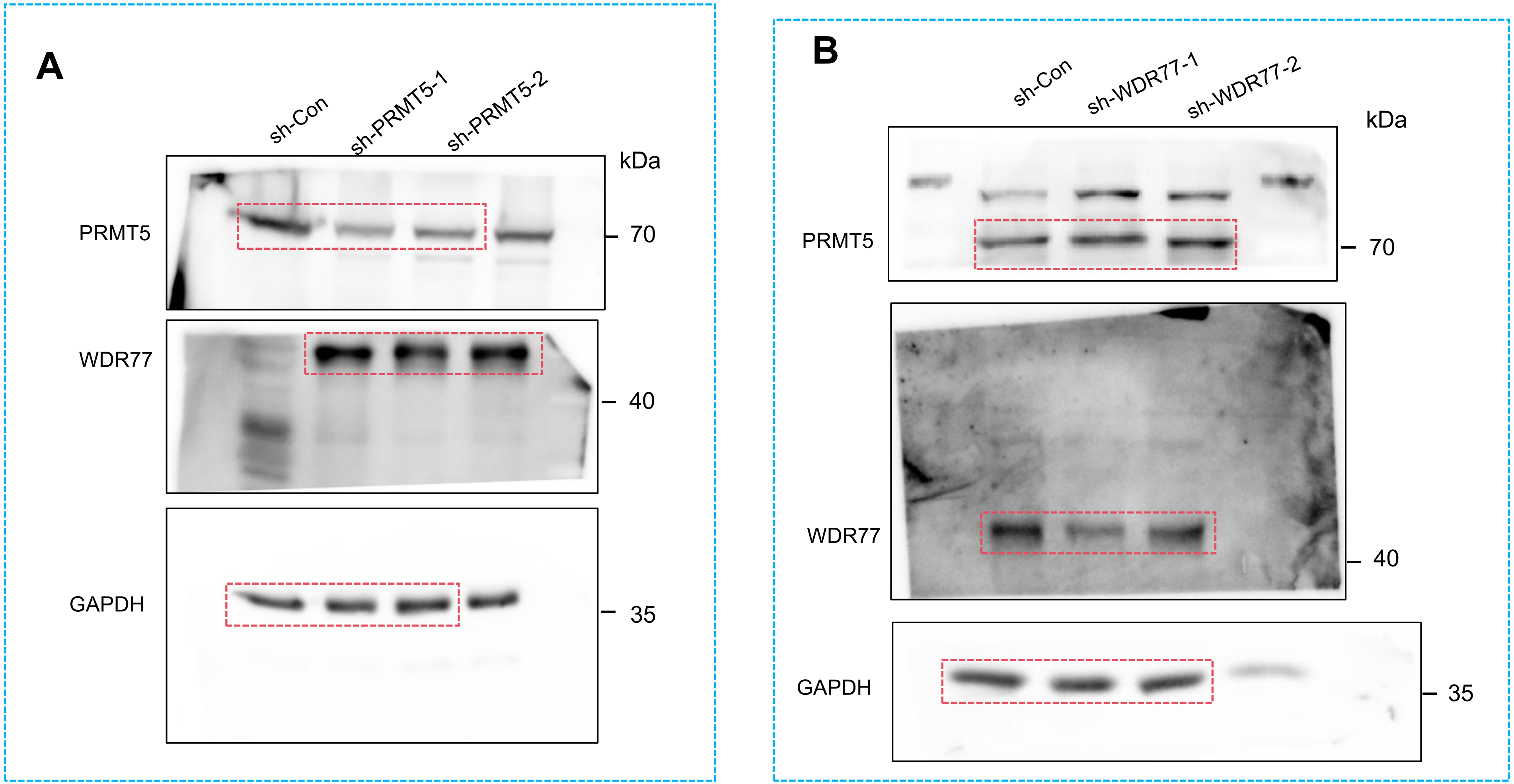
A
sh-Con
sh-PRMT5-1
sh-PRMT5-2
kDa
PRMT5
70
WDR77
40
GAPDH
35
B
sh-Con
sh-WDR77-1
sh-WDR77-2
kDa
PRMT5
70
WDR77
40
GAPDH
35

Figure 2

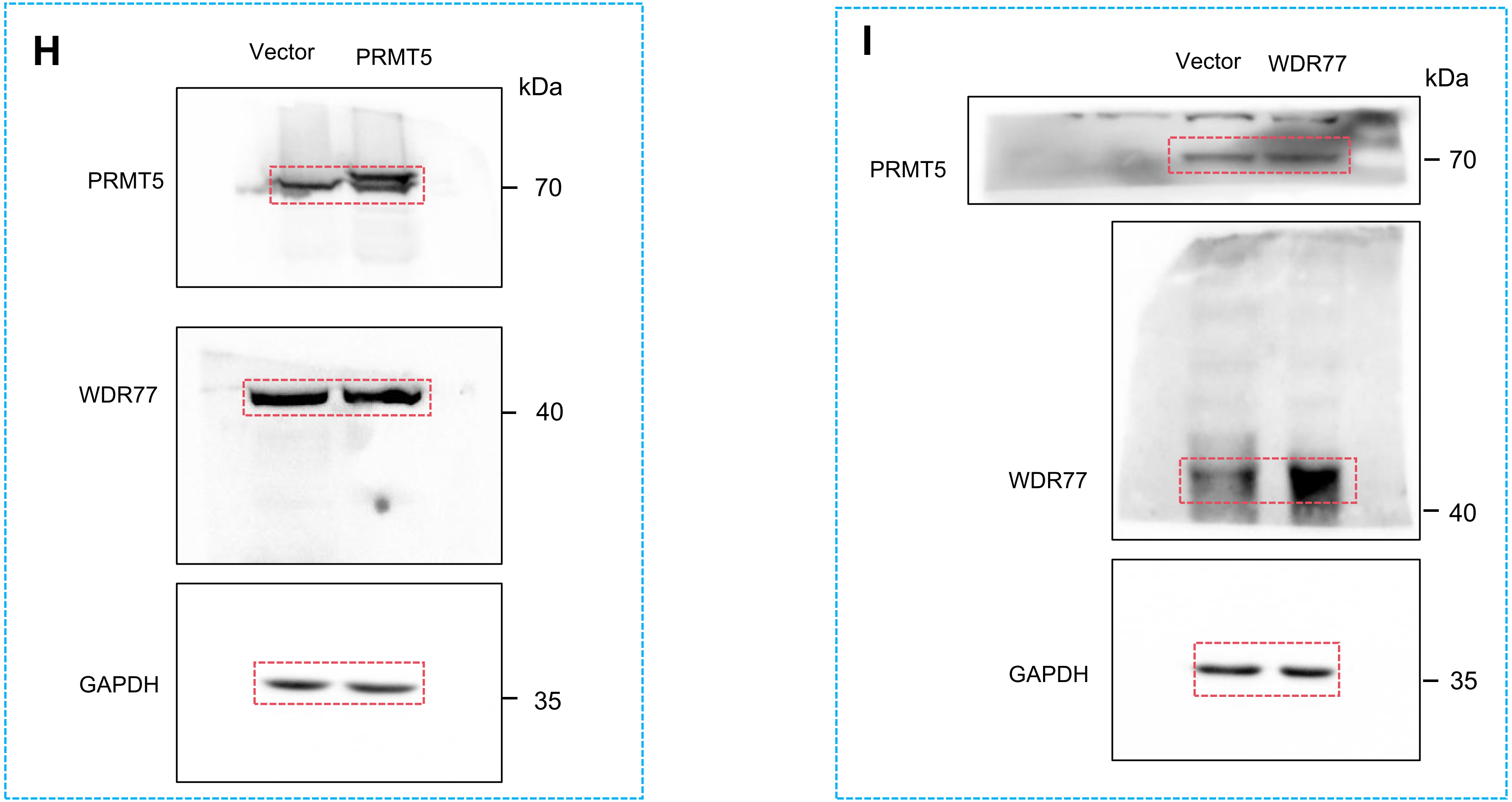

H
Vector
PRMT5
kDa
PRMT5
70
WDR77
40
GAPDH
35
I
Vector
WDR77
kDa
PRMT5
70
WDR77
40
GAPDH
35

Figure 3

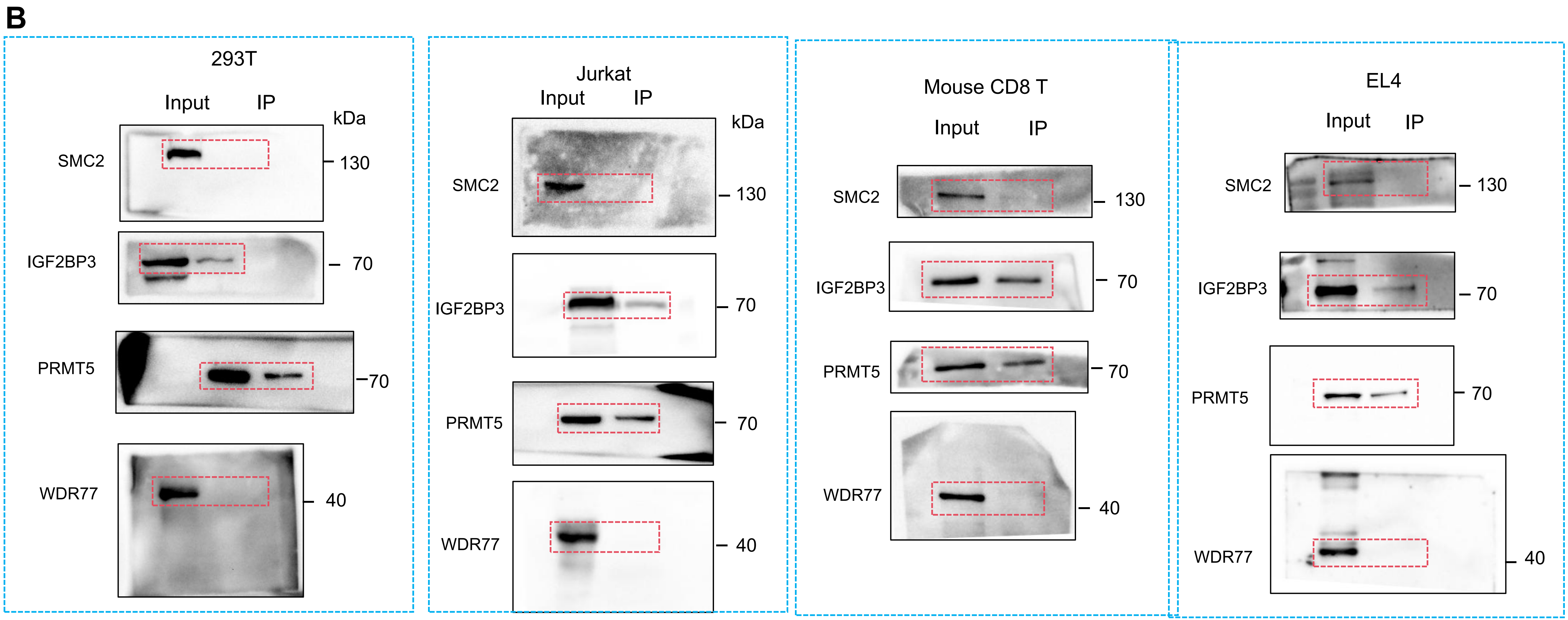
B
293T
Input
IP
kDa
SMC2
130
IGF2BP3
70
PRMT5
70
WDR77
40
Jurkat
Input
IP
kDa
SMC2
130
IGF2BP3
70
PRMT5
70
WDR77
40
Mouse CD8 T
Input
IP
SMC2
130
IGF2BP3
70
PRMT5
70
WDR77
40
EL4
Input
IP
SMC2
130
IGF2BP3
70
PRMT5
70
WDR77
40

Figure 3

C

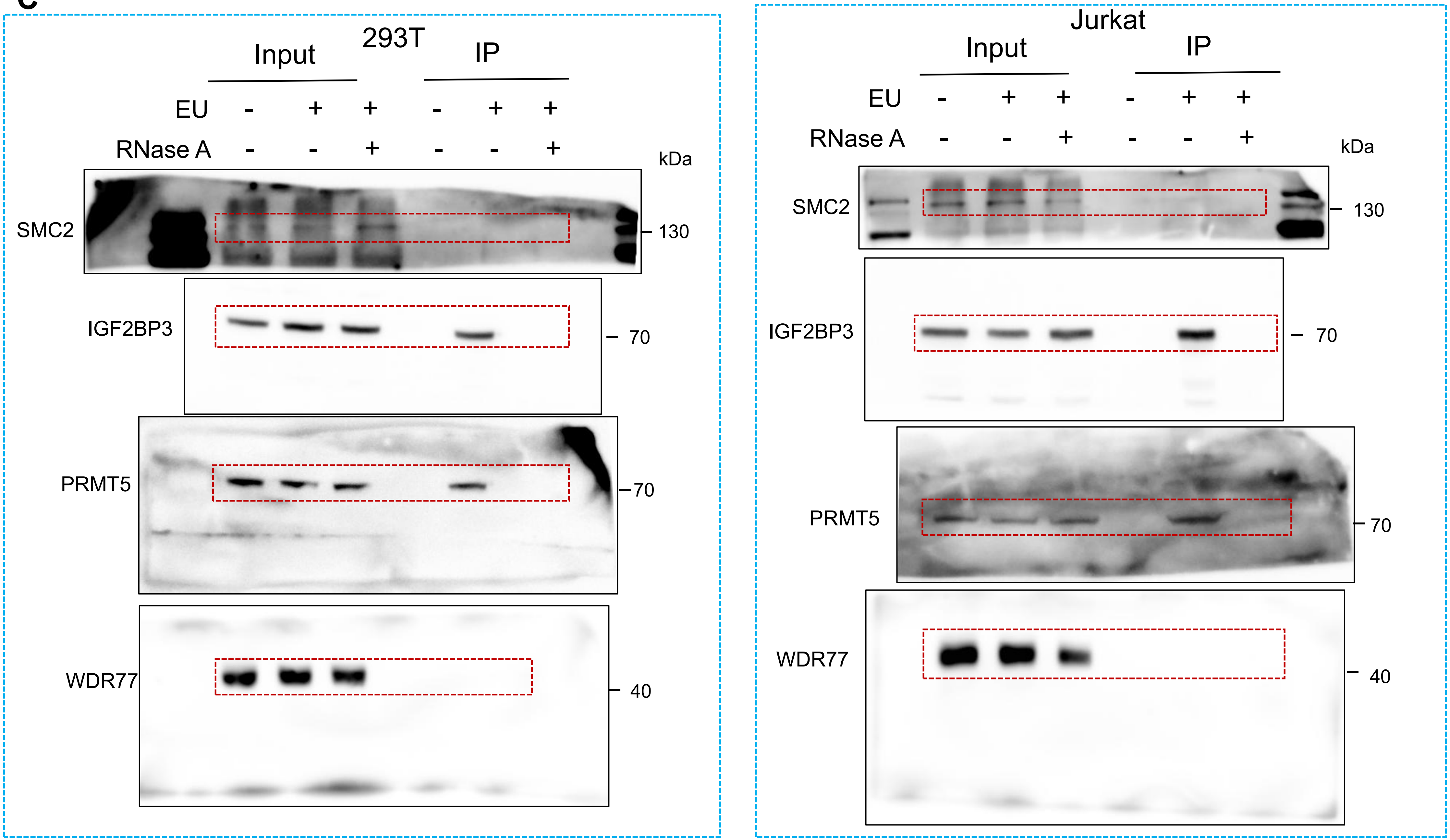

293T
Input
IP
EU - + + - + +
RNase A - - + - - +
kDa
SMC2
130
IGF2BP3
70
PRMT5
70
WDR77
40
Jurkat
Input
IP
EU - + + - + +
RNase A - - + - - +
kDa
SMC2
130
IGF2BP3
70
PRMT5
70
WDR77
40

Figure 3

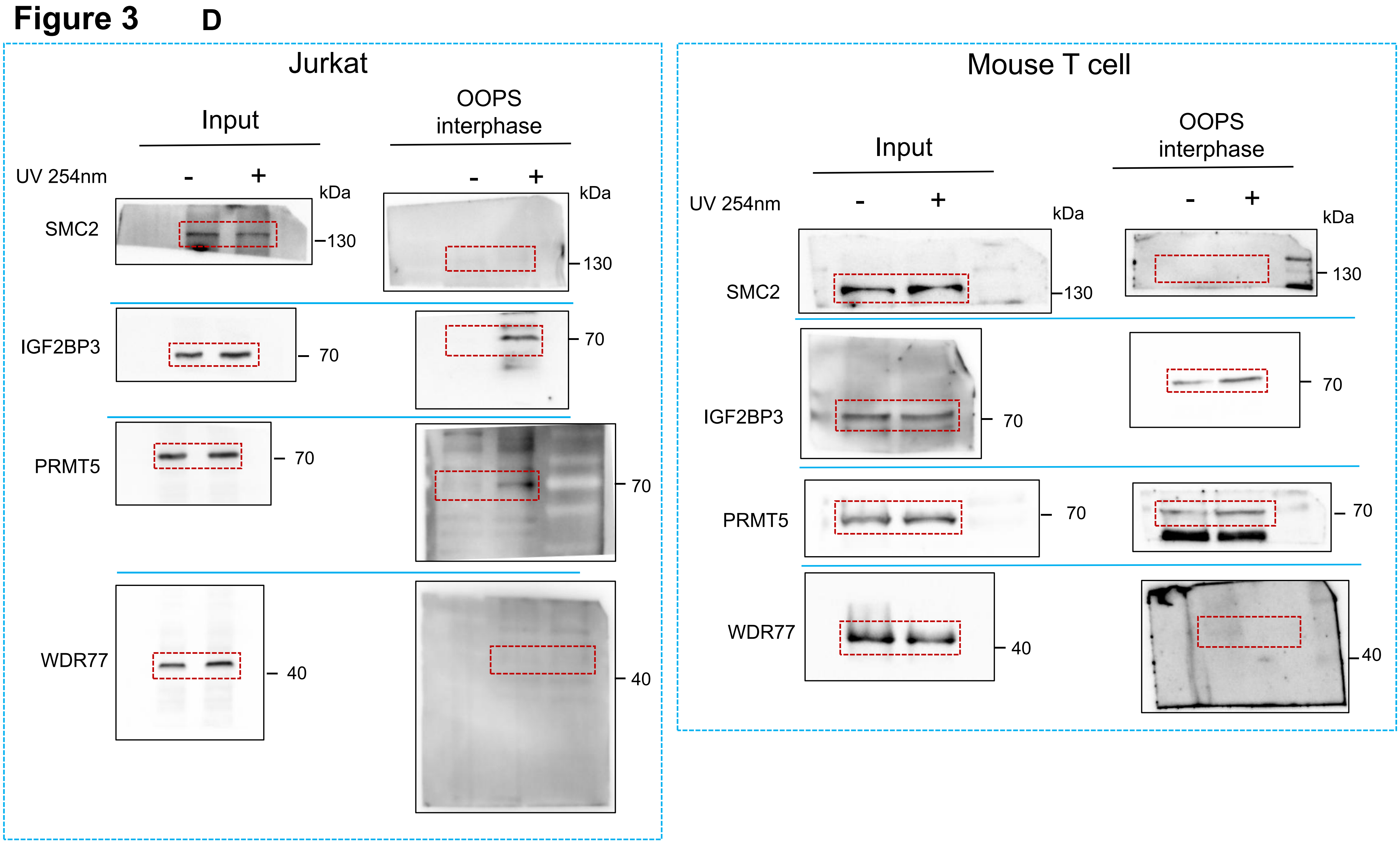
D
Jurkat
Input
OOPS interphase
UV 254nm
-
+
kDa
SMC2
130
IGF2BP3
70
PRMT5
70
WDR77
40
Mouse T cell
Input
OOPS interphase
UV 254nm
-
+
kDa
SMC2
130
IGF2BP3
70
PRMT5
70
WDR77
40

Figure 3

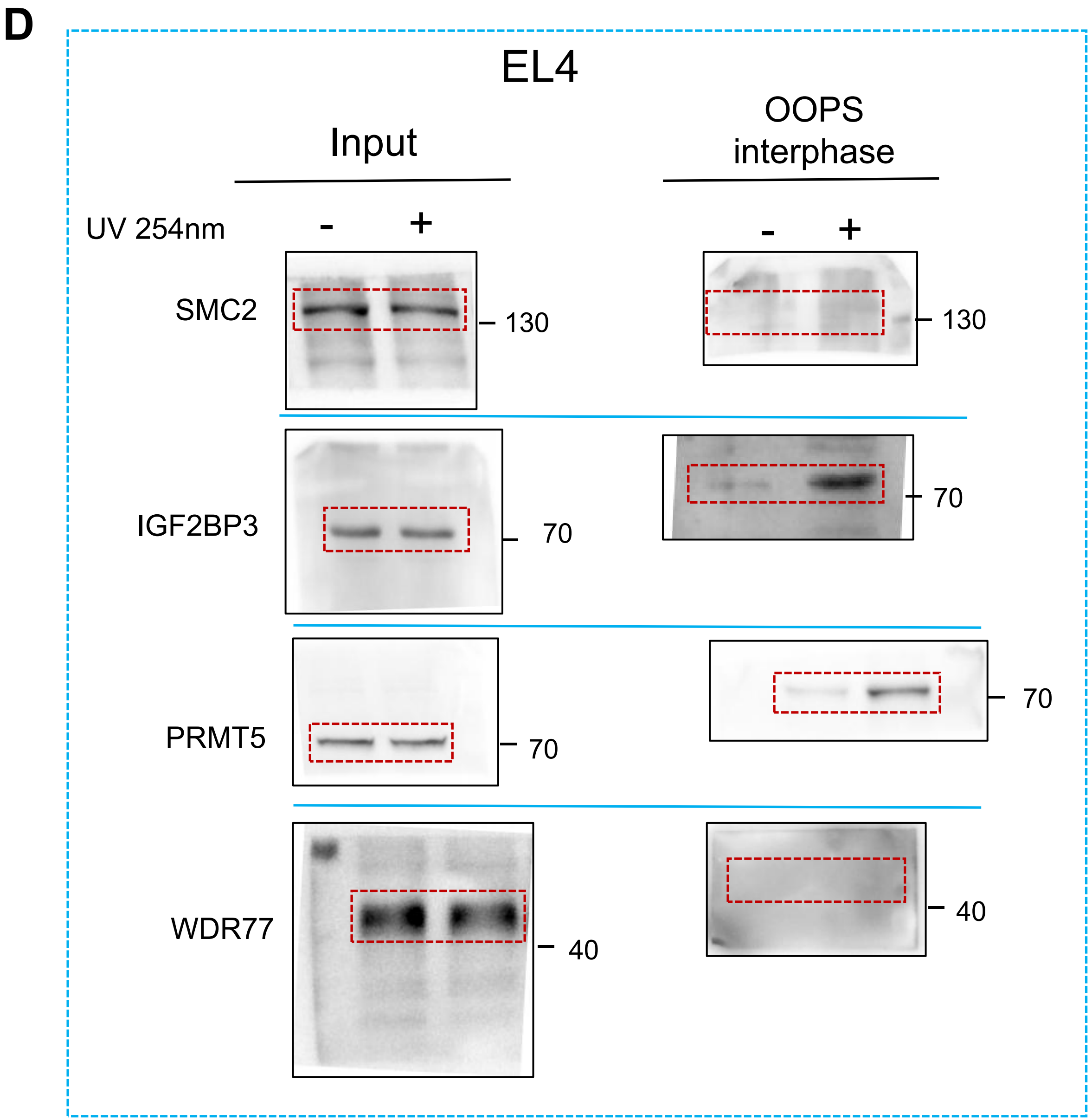
D
EL4
Input
OOPS
interphase
UV 254nm
-
+
-
+
SMC2
130
130
IGF2BP3
70
70
PRMT5
70
70
WDR77
40
40

Figure 3

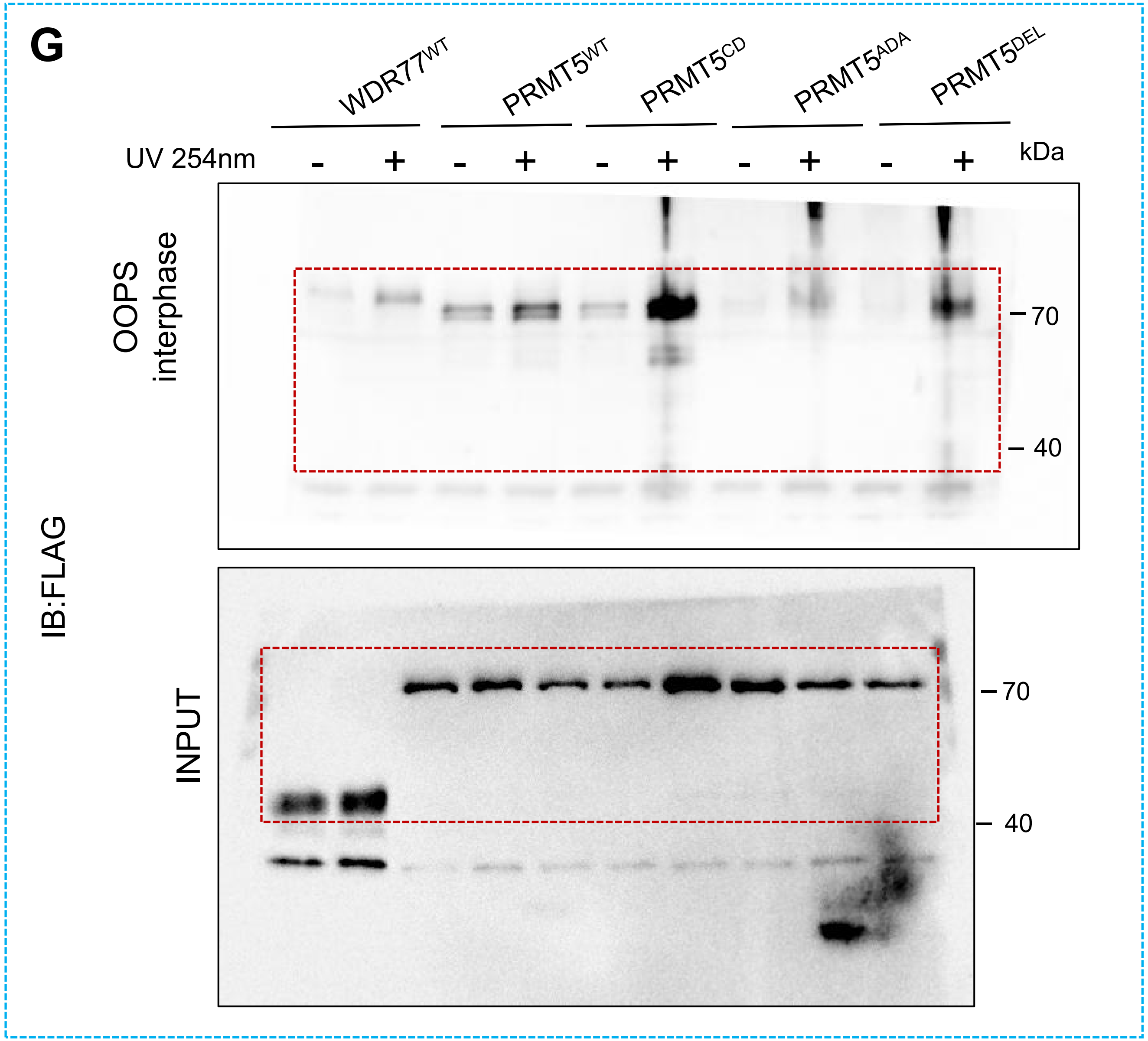
G
WDR77^WT
PRMT5^WT
PRMT5^CD
PRMT5^ADA
PRMT5^DEL
UV 254nm
- + - + - + - + - +
kDa
OOPS interphase
70
40
IB:FLAG
INPUT
70
40

Figure 3

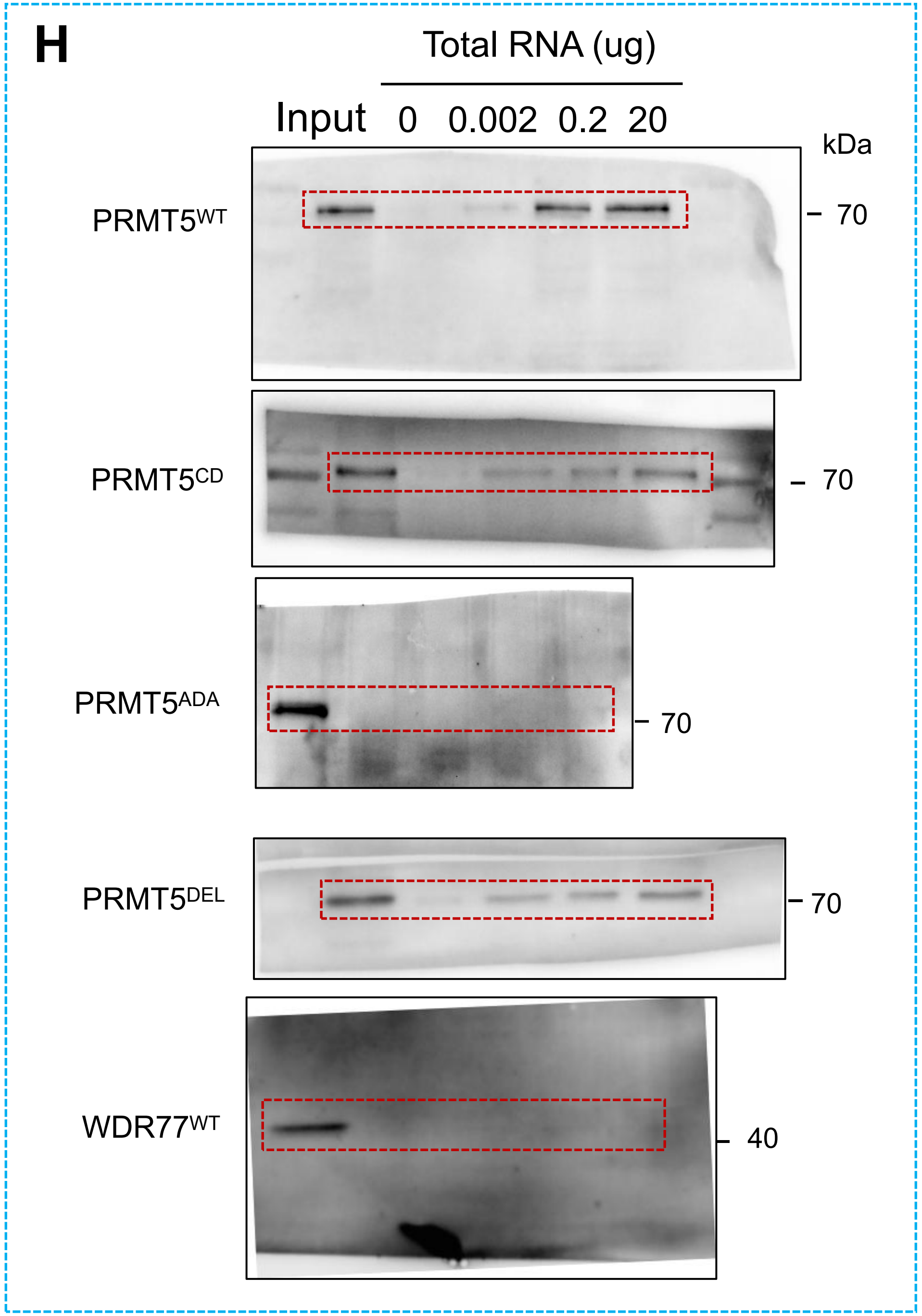
H
Total RNA (ug)
Input
0
0.002
0.2
20
kDa
PRMT5WT
70
PRMT5CD
70
PRMT5ADA
70
PRMT5DEL
70
WDR77WT
40

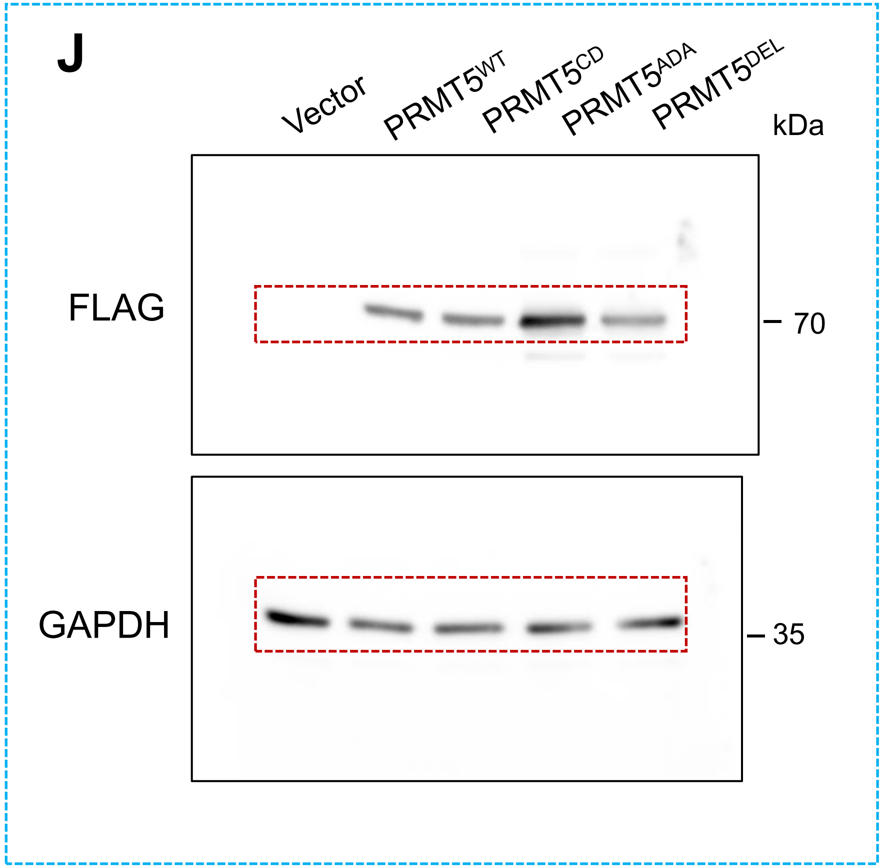
J
Vector
PRMT5WT
PRMT5CD
PRMT5ADA
PRMT5DEL
kDa
FLAG
70
GAPDH
35

Figure 4

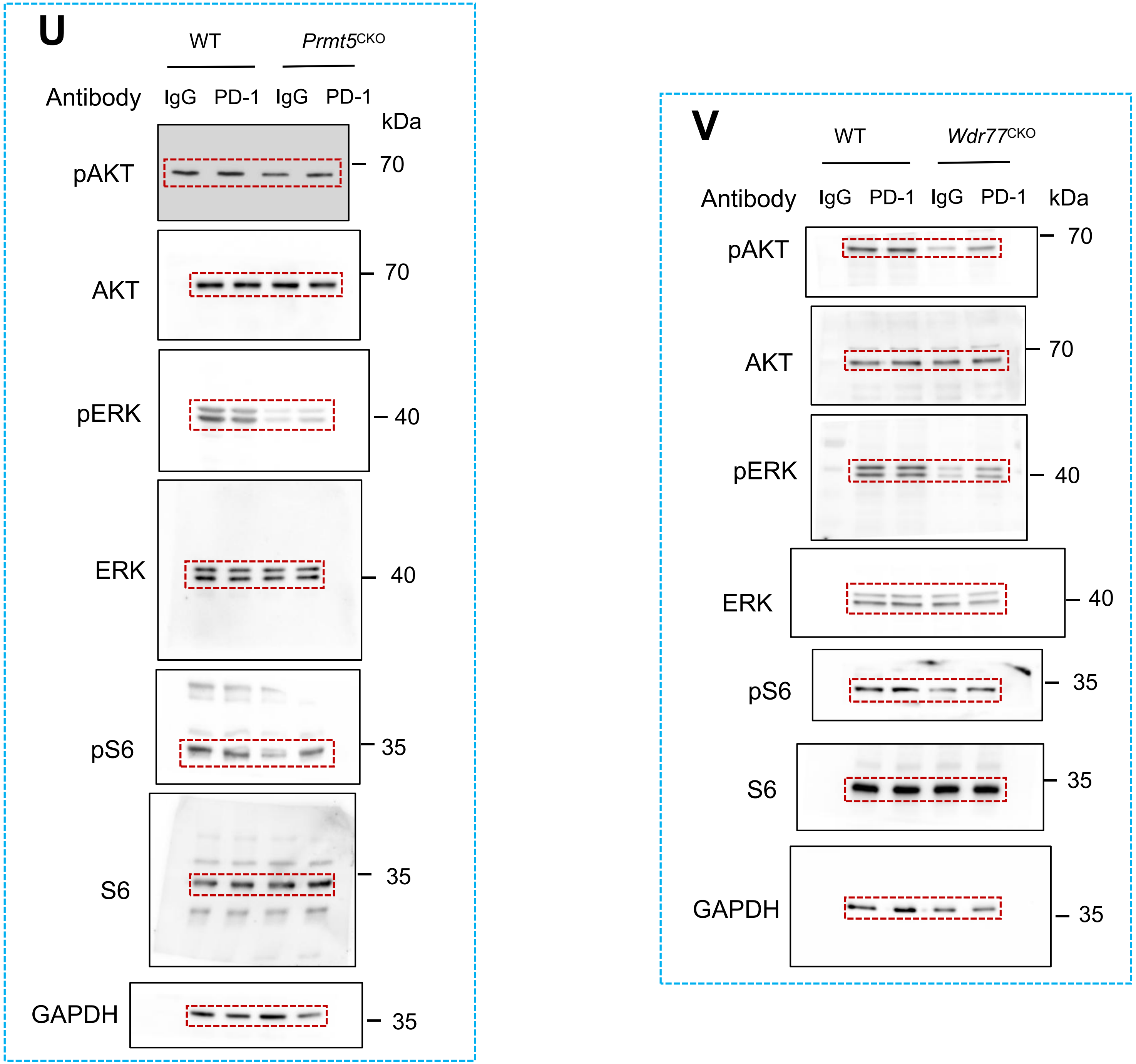
U
WT
Prmt5CKO
Antibody
IgG
PD-1
IgG
PD-1
kDa
pAKT
70
AKT
70
pERK
40
ERK
40
pS6
35
S6
35
GAPDH
35
V
WT
Wdr77CKO
Antibody
IgG
PD-1
IgG
PD-1
kDa
pAKT
70
AKT
70
pERK
40
ERK
40
pS6
35
S6
35
GAPDH
35

Figure 6

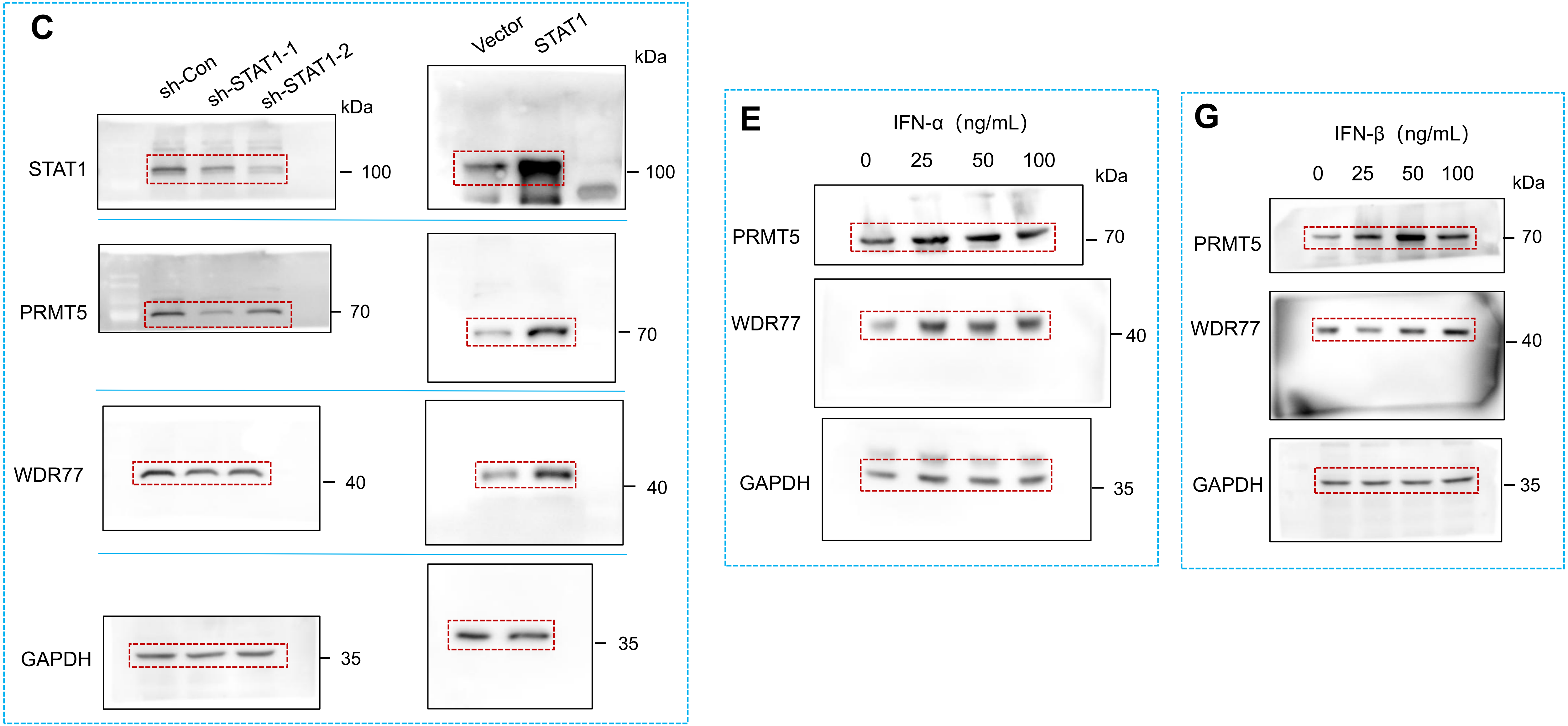

C
sh-Con
sh-STAT1-1
sh-STAT1-2
Vector
STAT1
kDa
STAT1
100
PRMT5
70
WDR77
40
GAPDH
35
E
IFN-α (ng/mL)
0
25
50
100
kDa
PRMT5
70
WDR77
40
GAPDH
35
G
IFN-β (ng/mL)
0
25
50
100
kDa
PRMT5
70
WDR77
40
GAPDH
35

Supplemental Figure 1

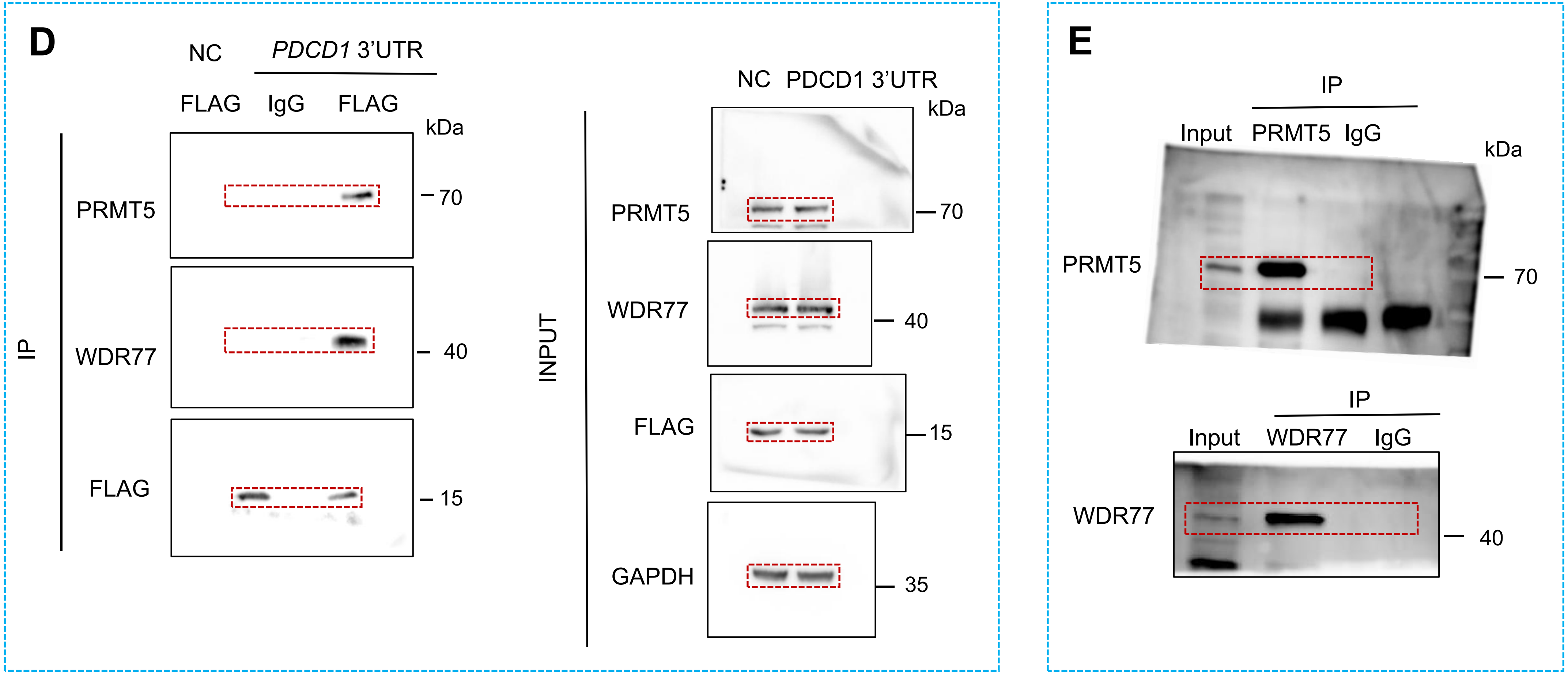

Supplemental Figure 1

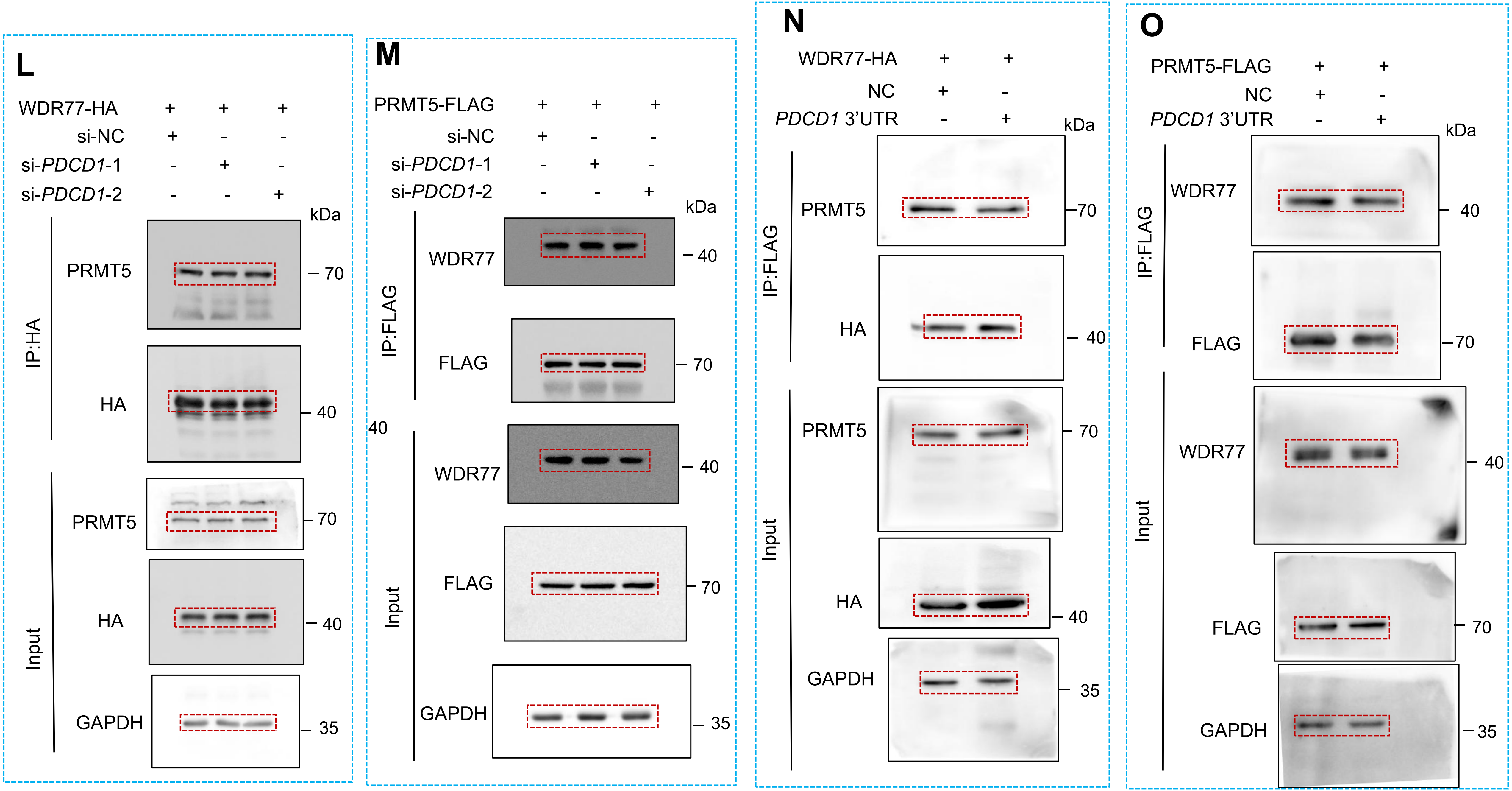

Supplemental Figure 2

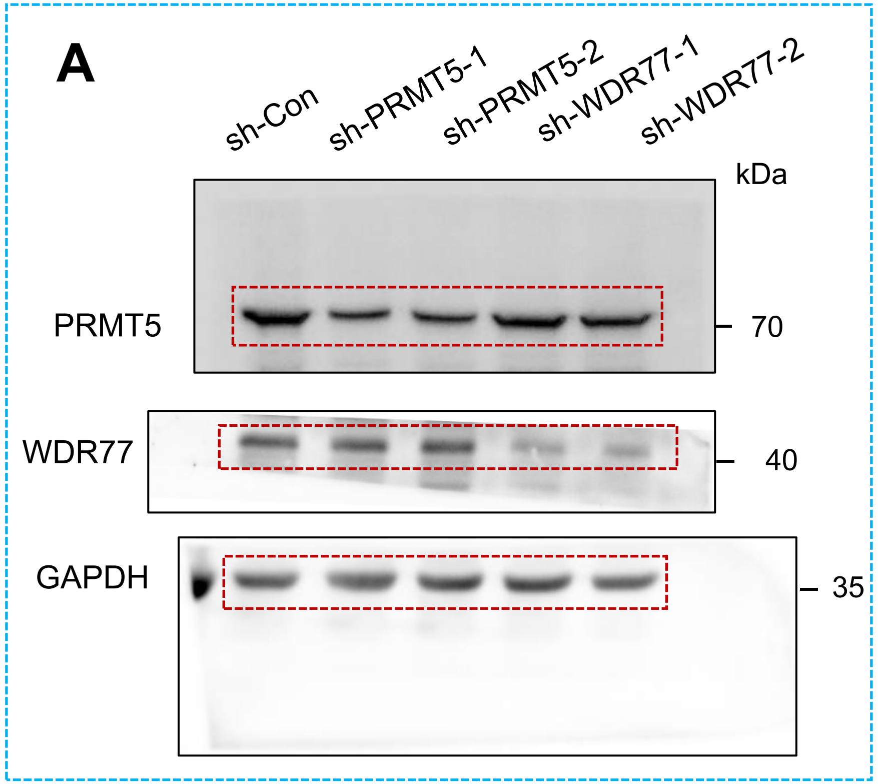

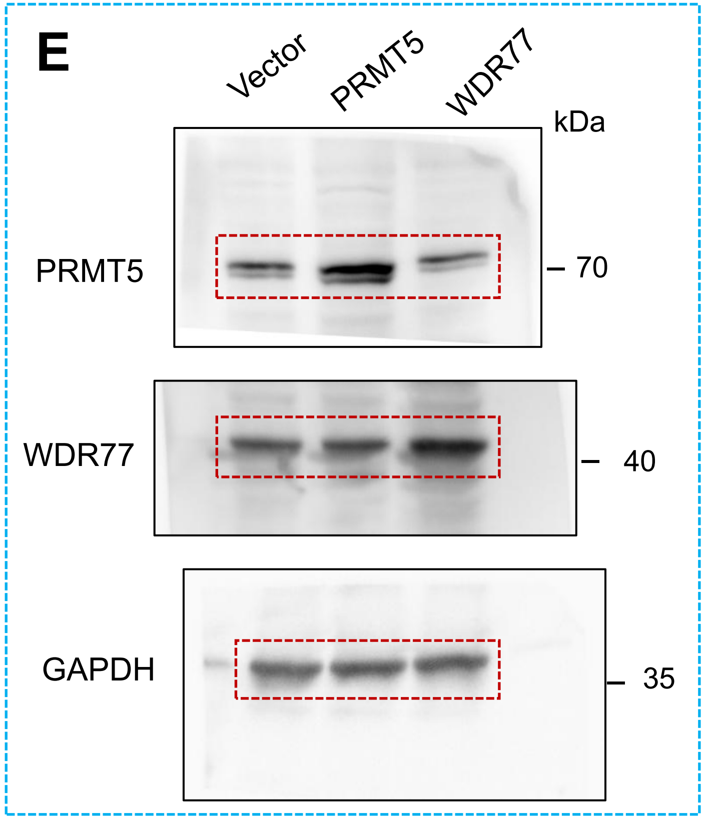

Supplemental Figure 3

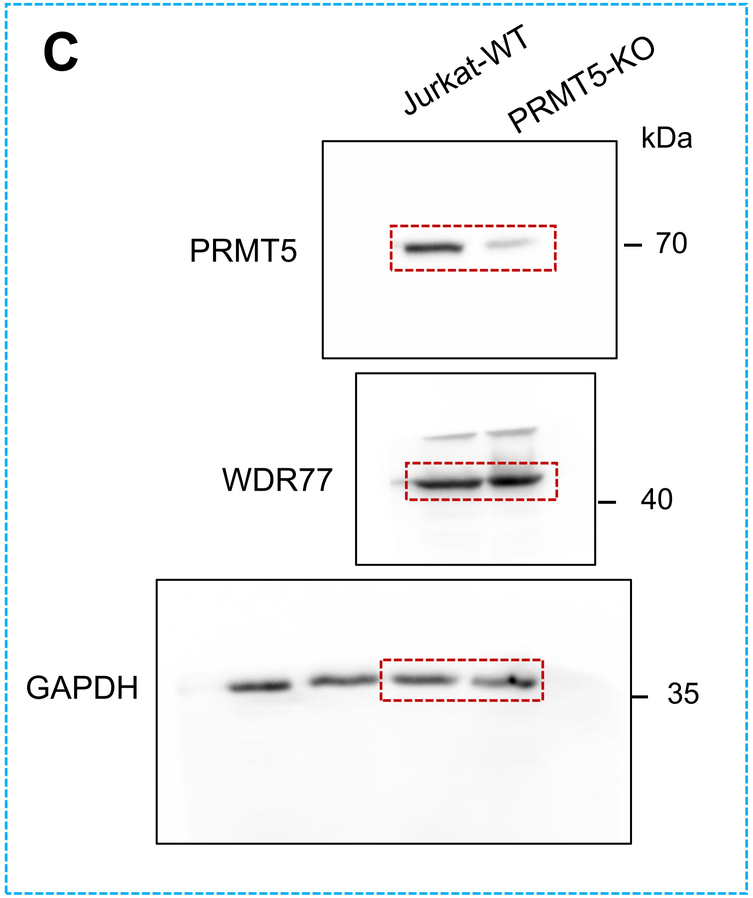

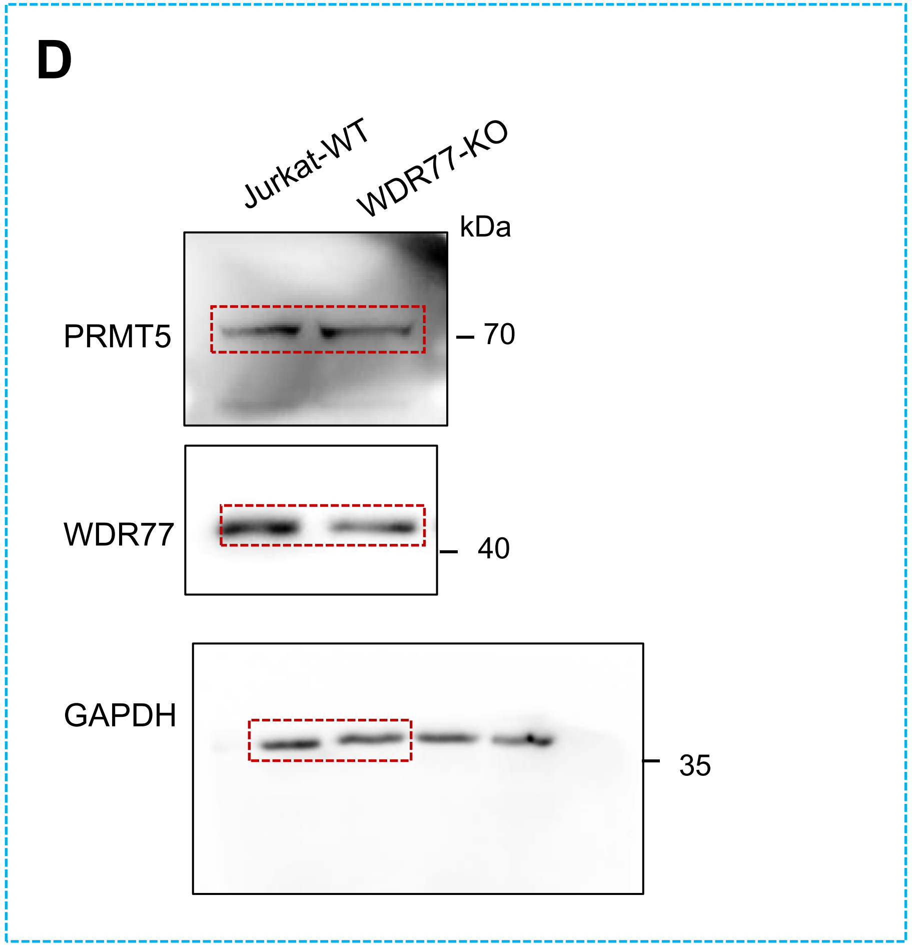

Supplemental Figure 4

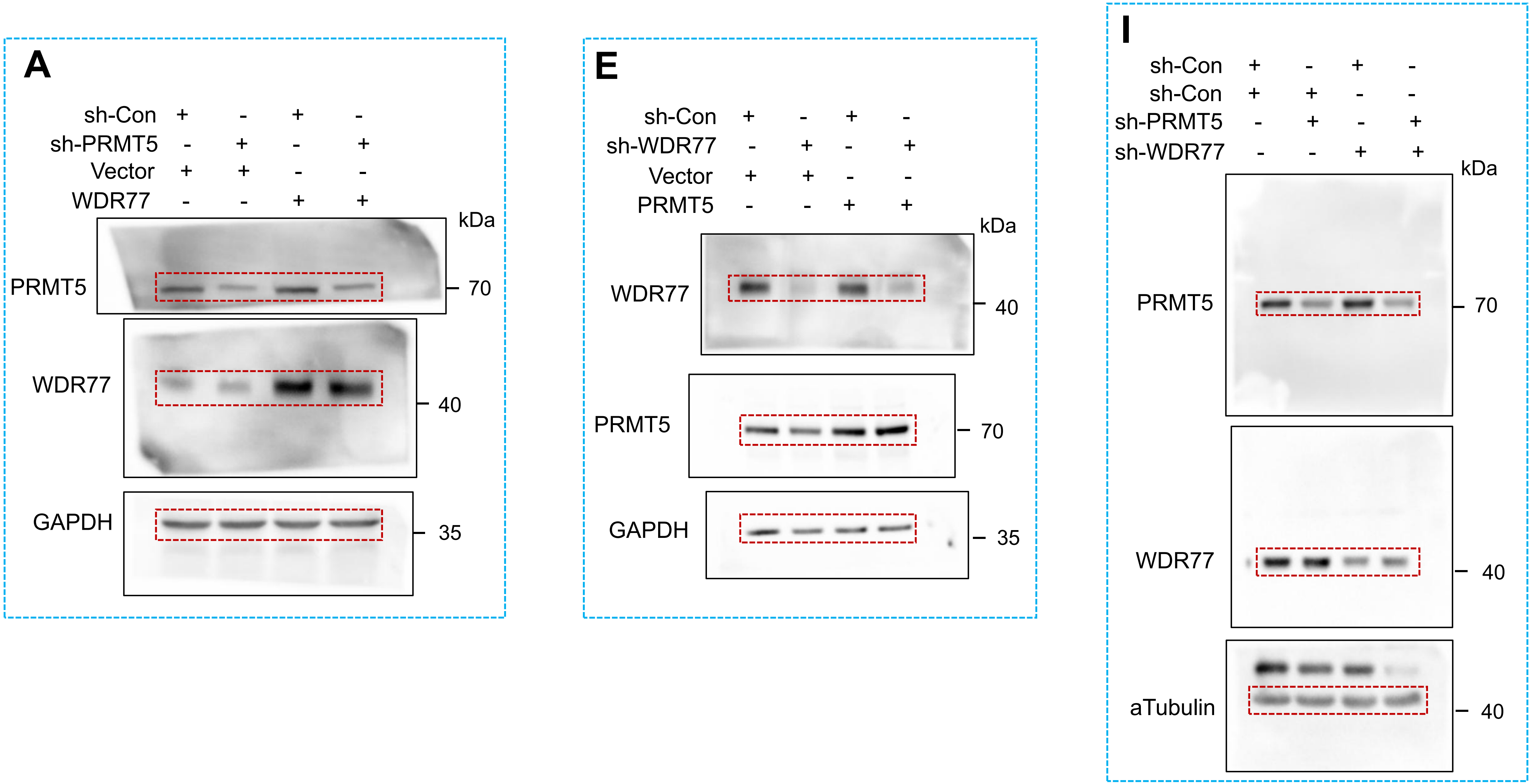

Supplemental Figure 4

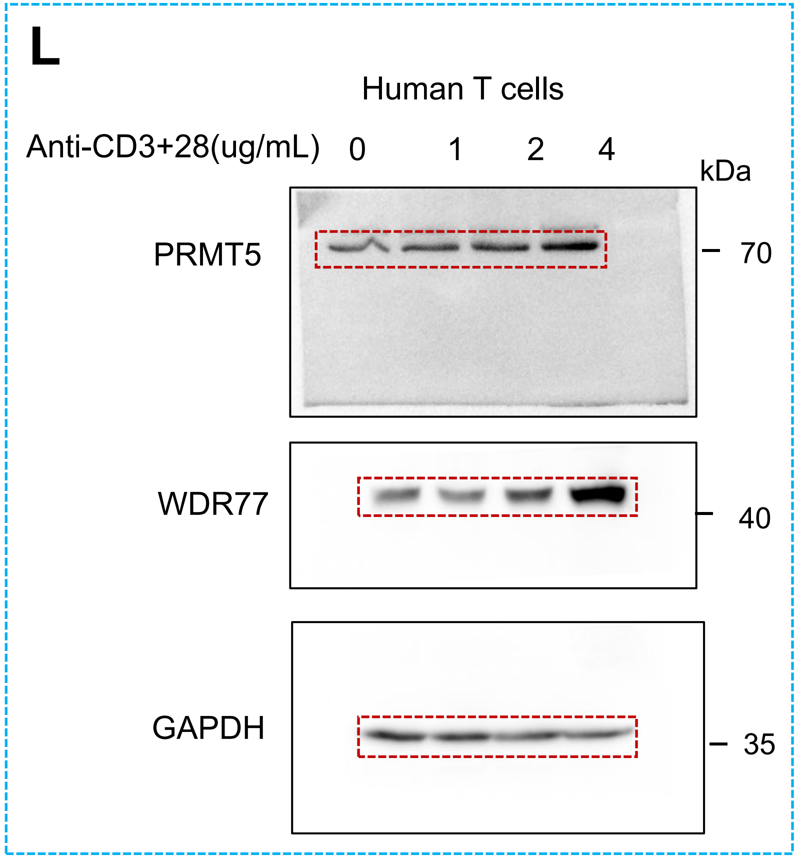

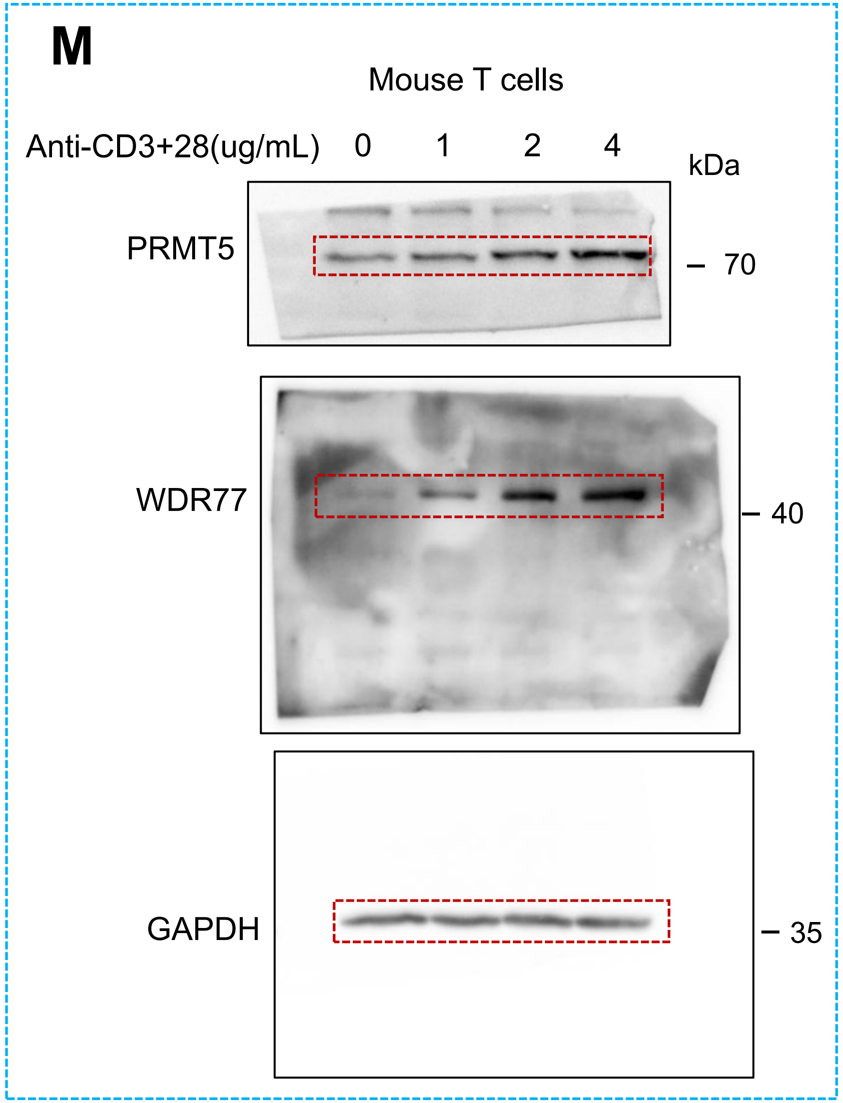

Supplemental Figure 5

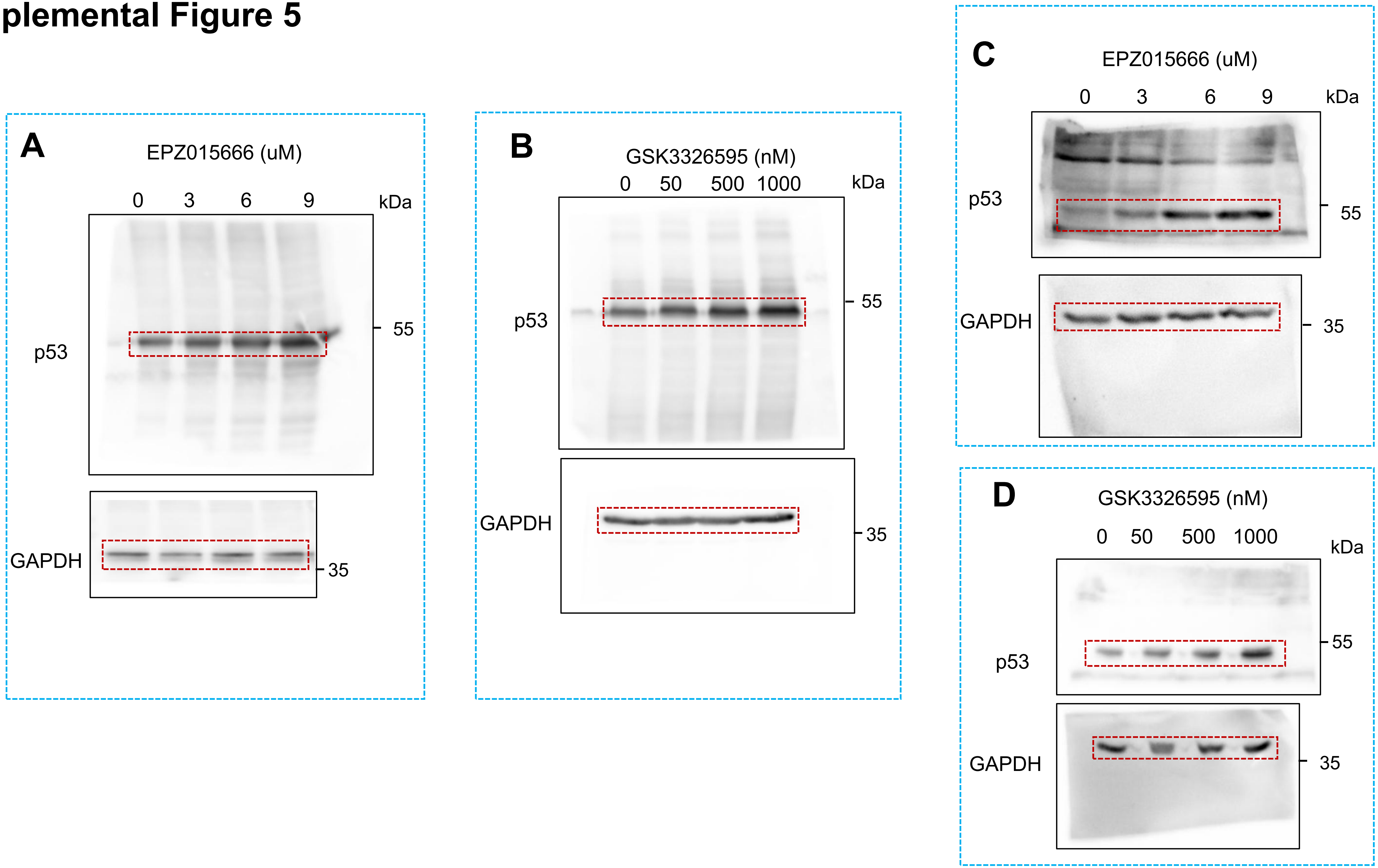

Supplemental Figure 6

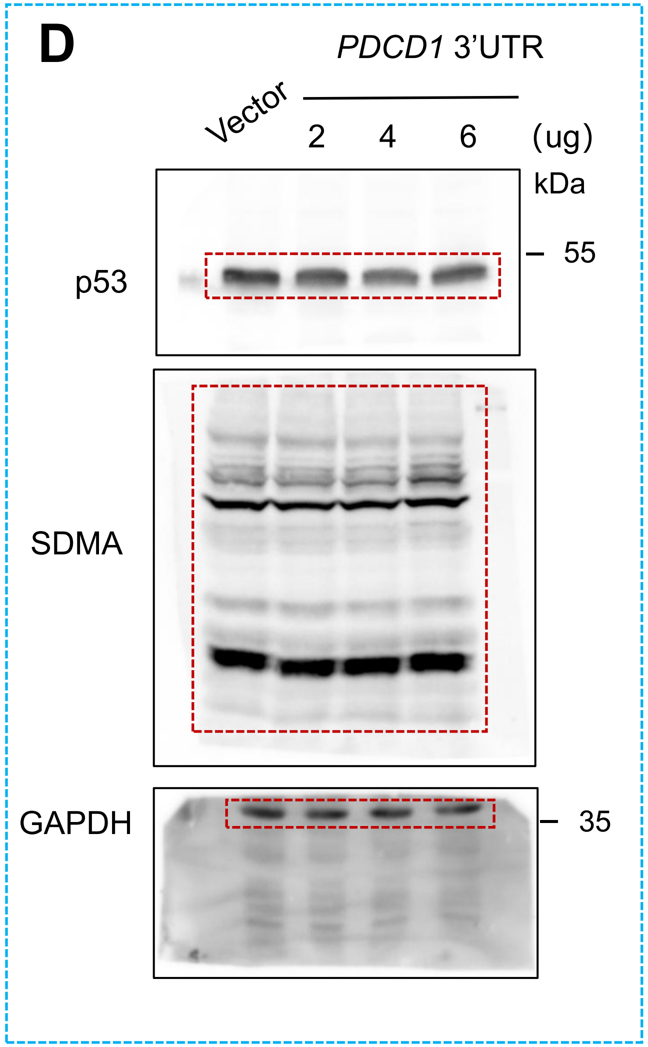

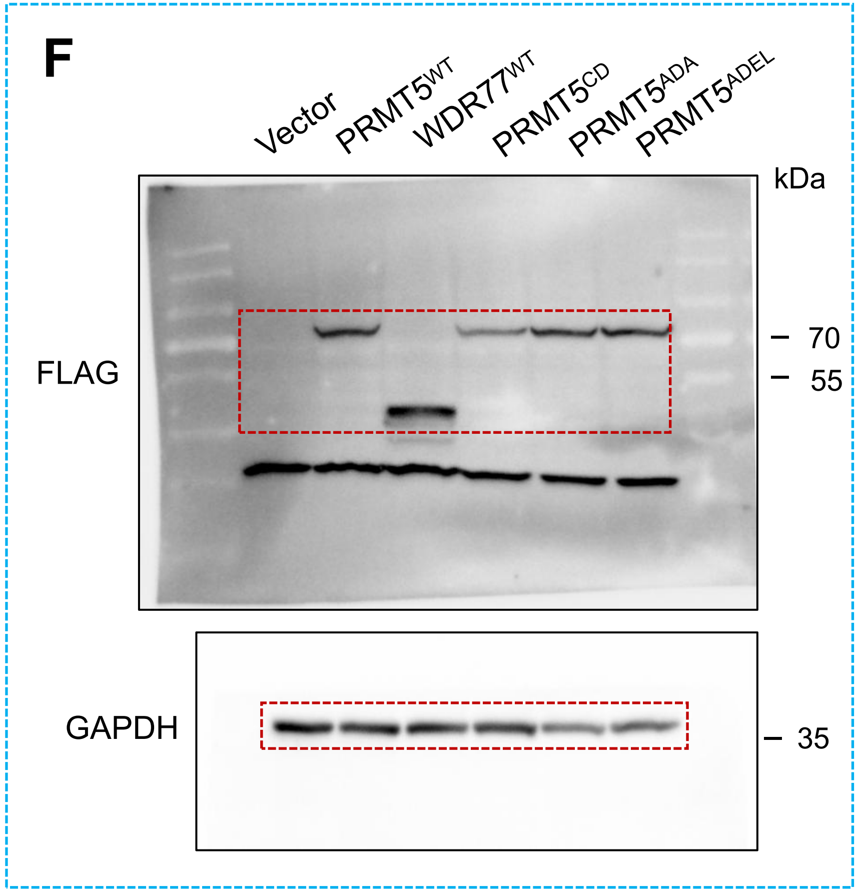

Supplemental Figure 7

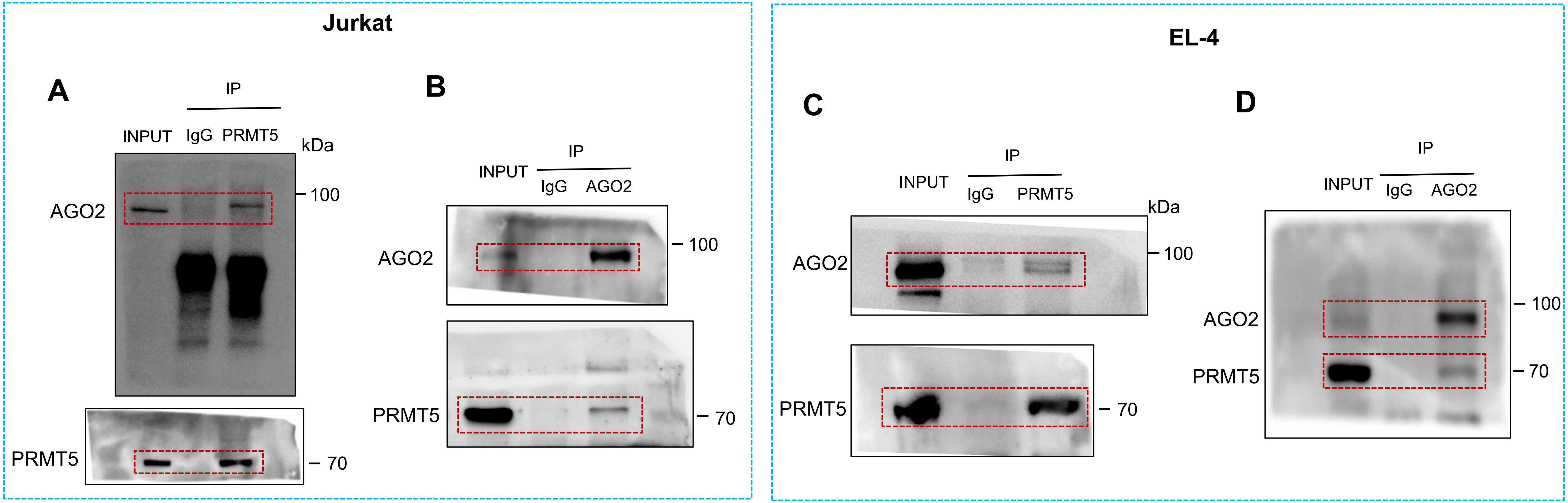

Supplemental Figure 7

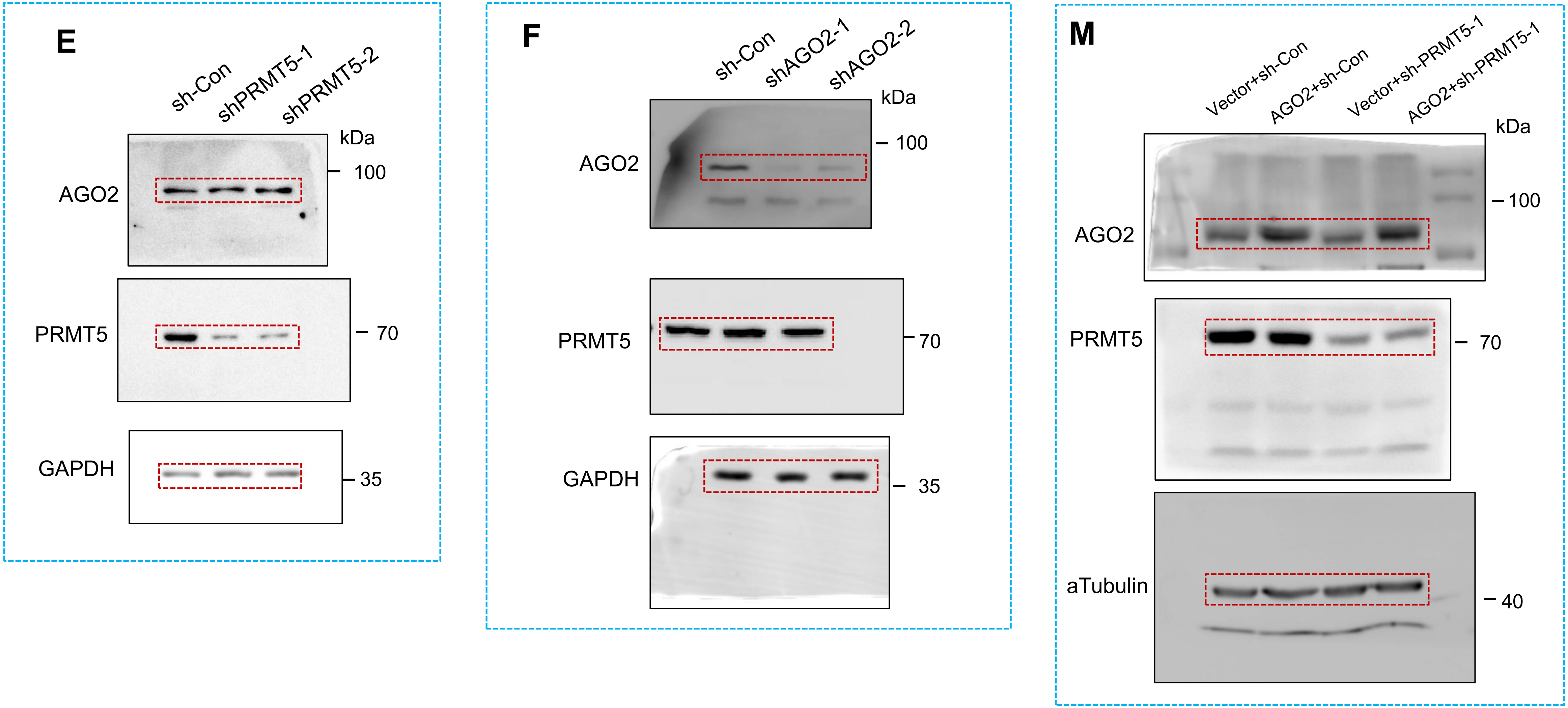

Supplemental Figure 8

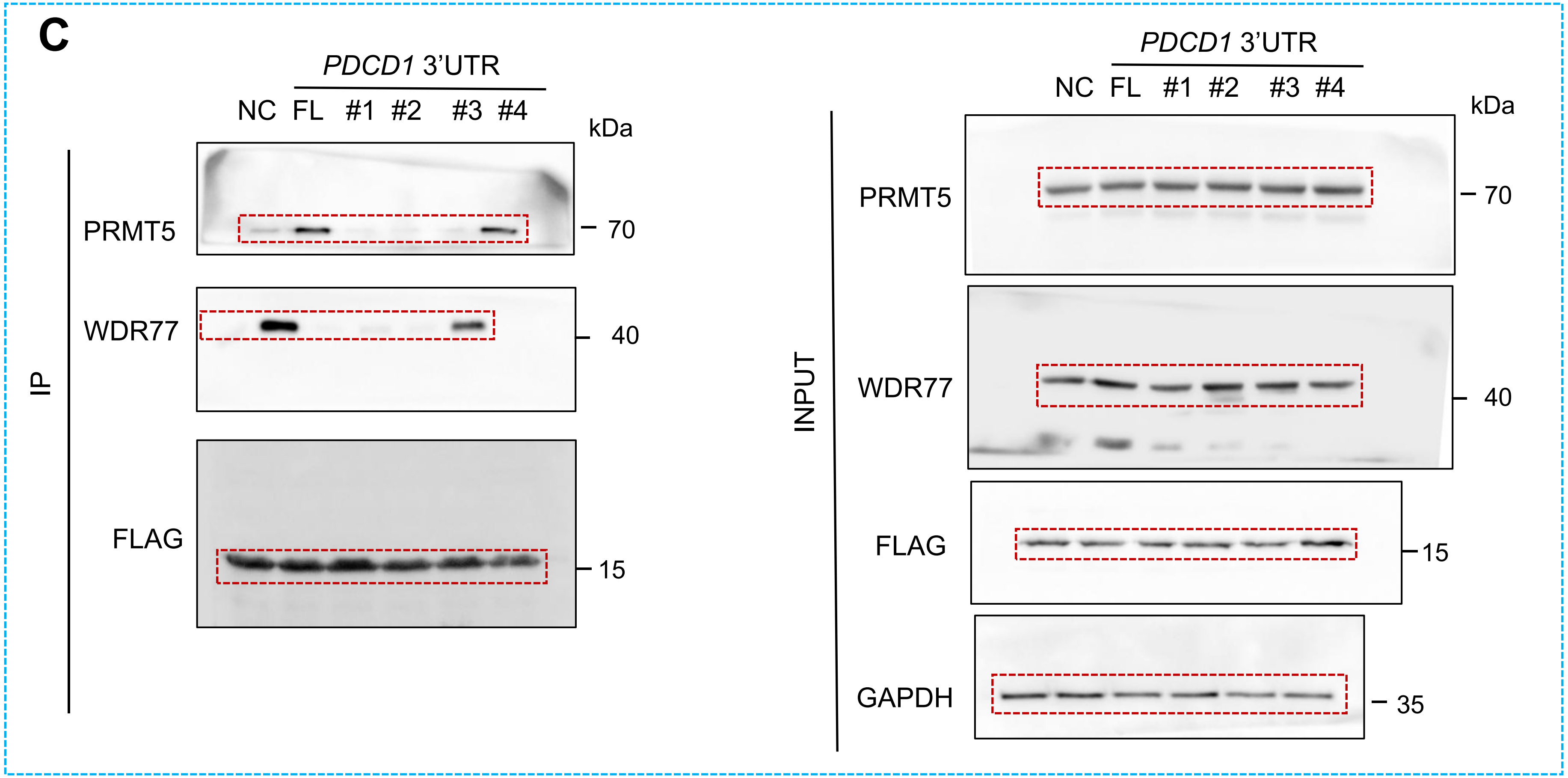

Supplemental Figure 8

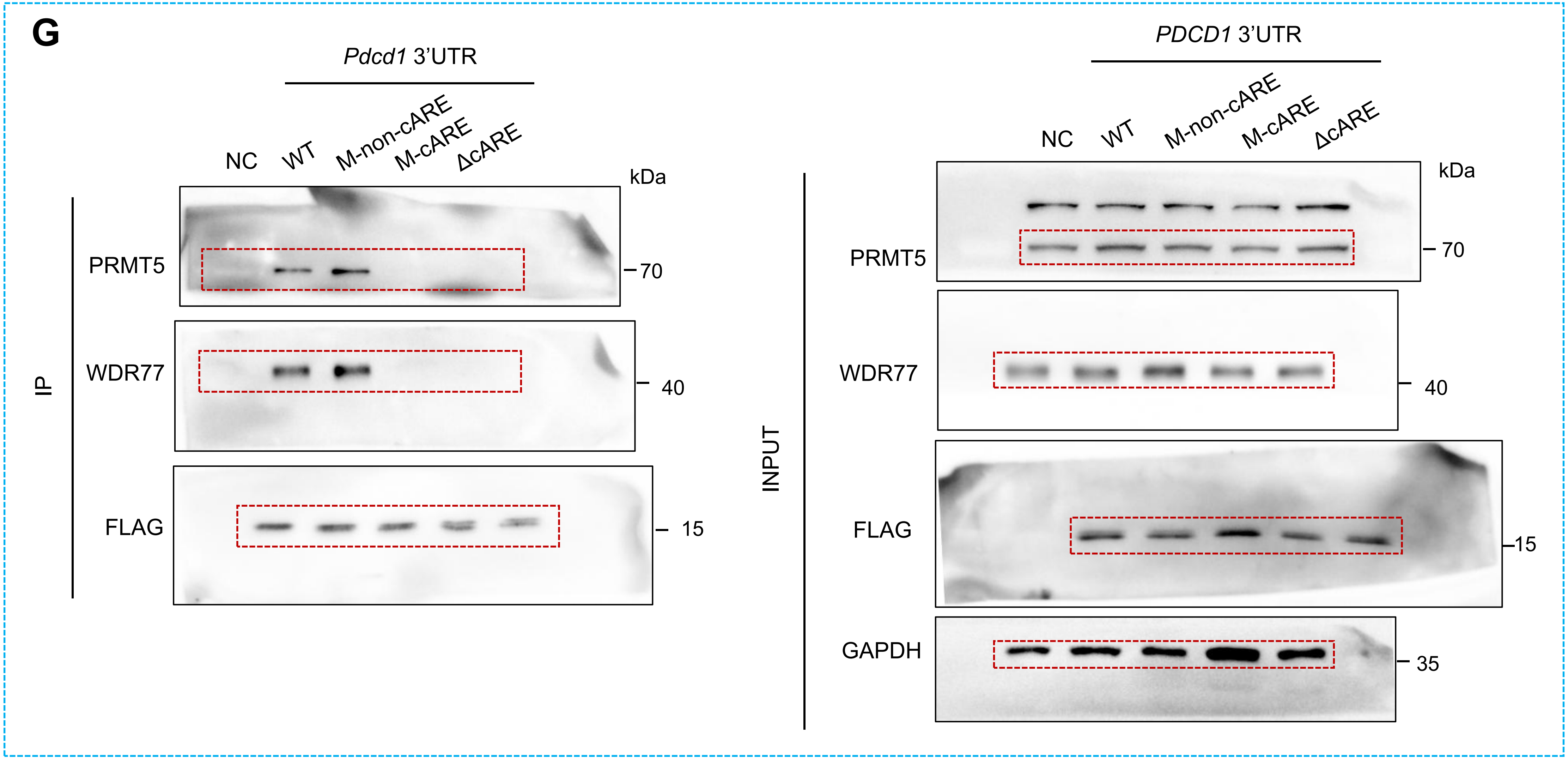

Supplemental Figure 8

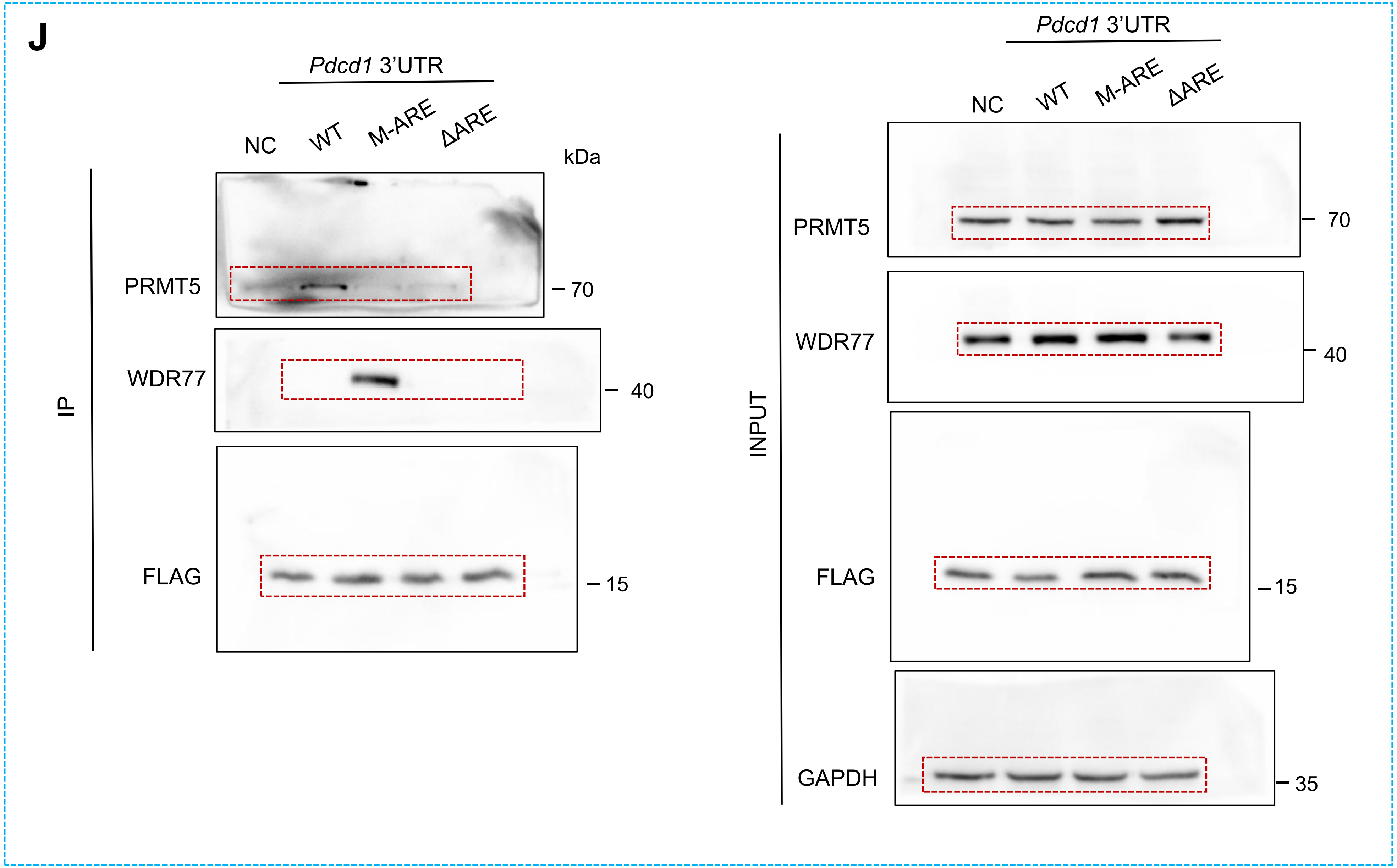

Supplemental Figure 9

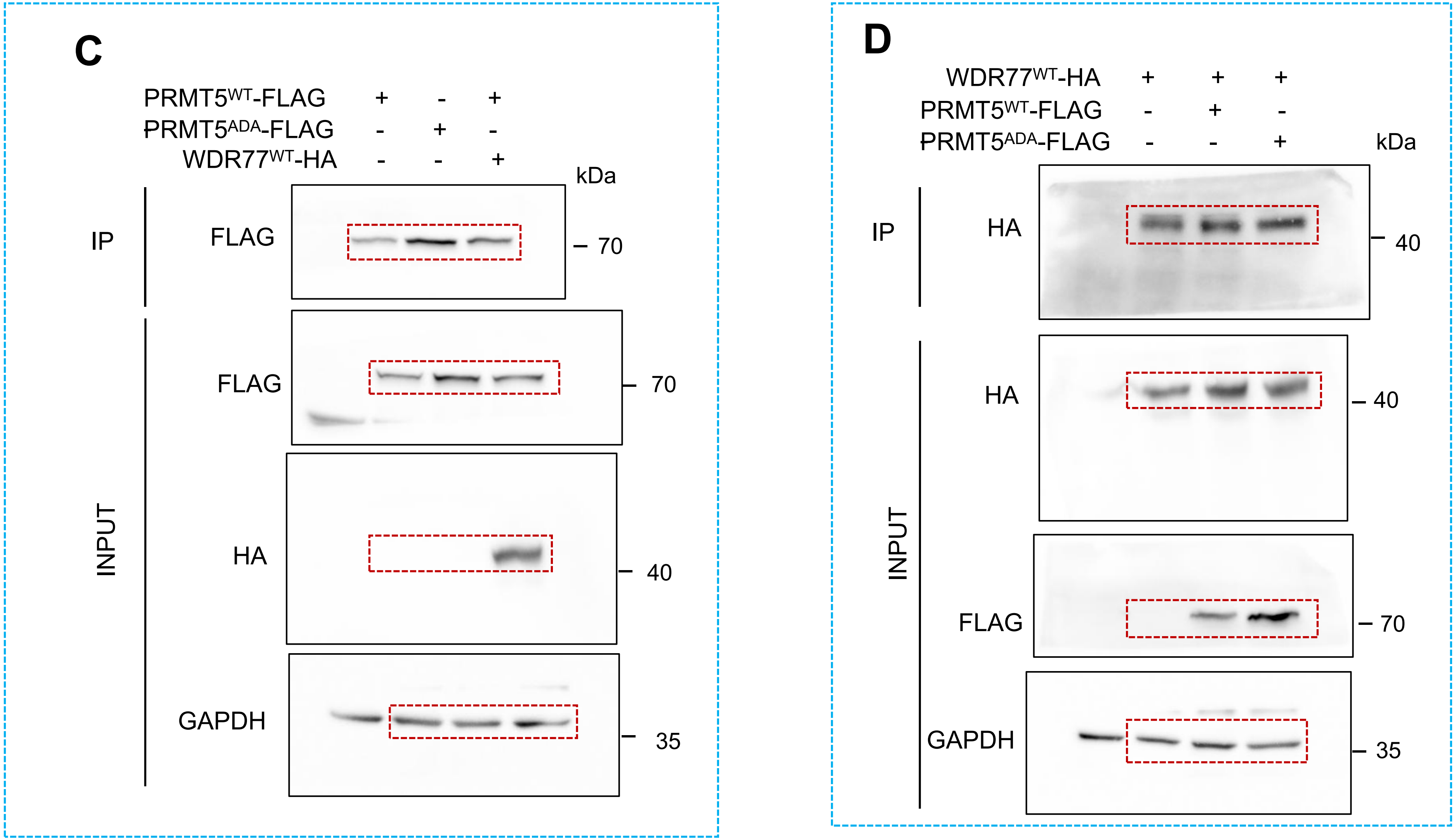

Supplemental Figure 9

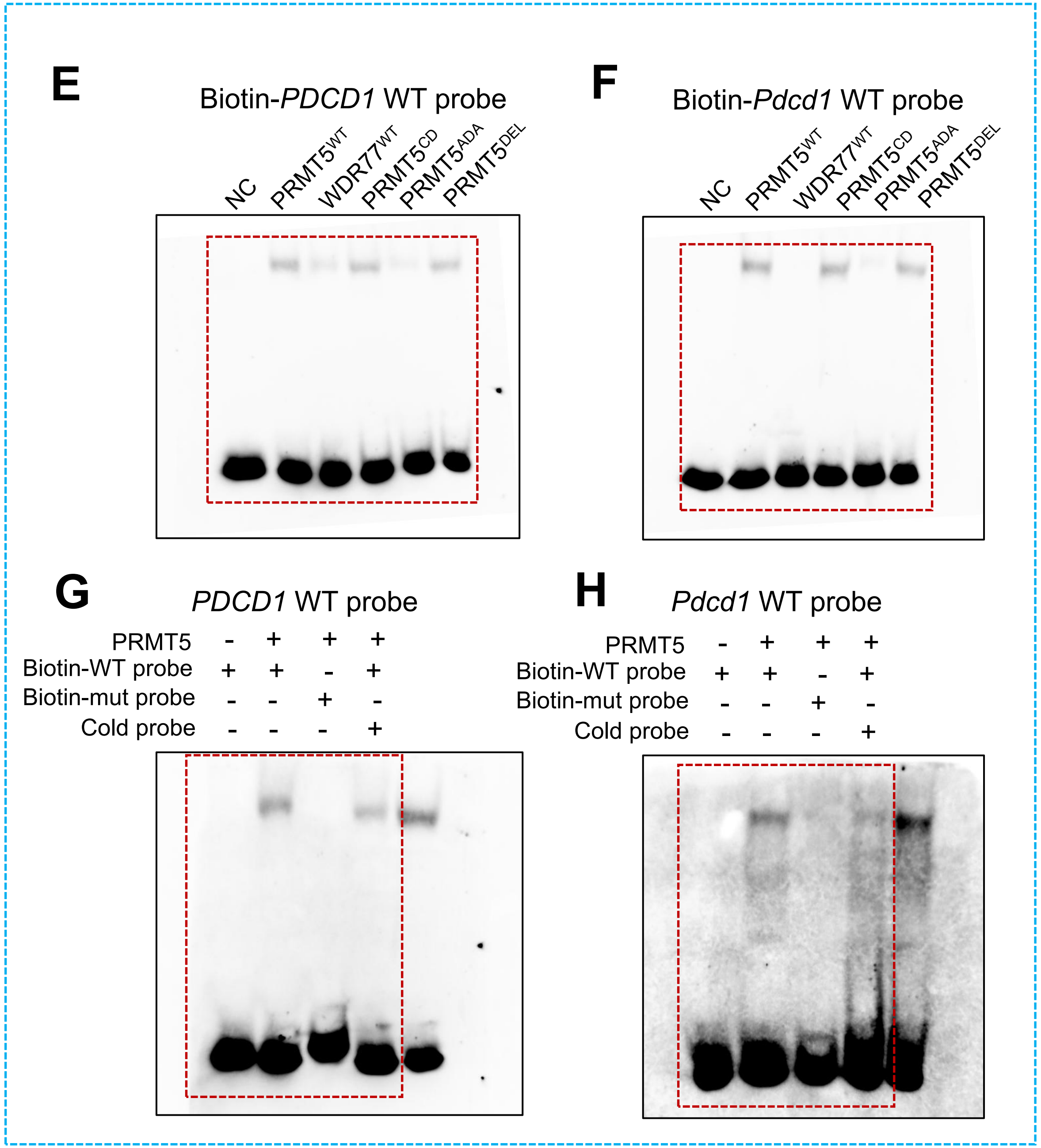

Supplemental Figure 10

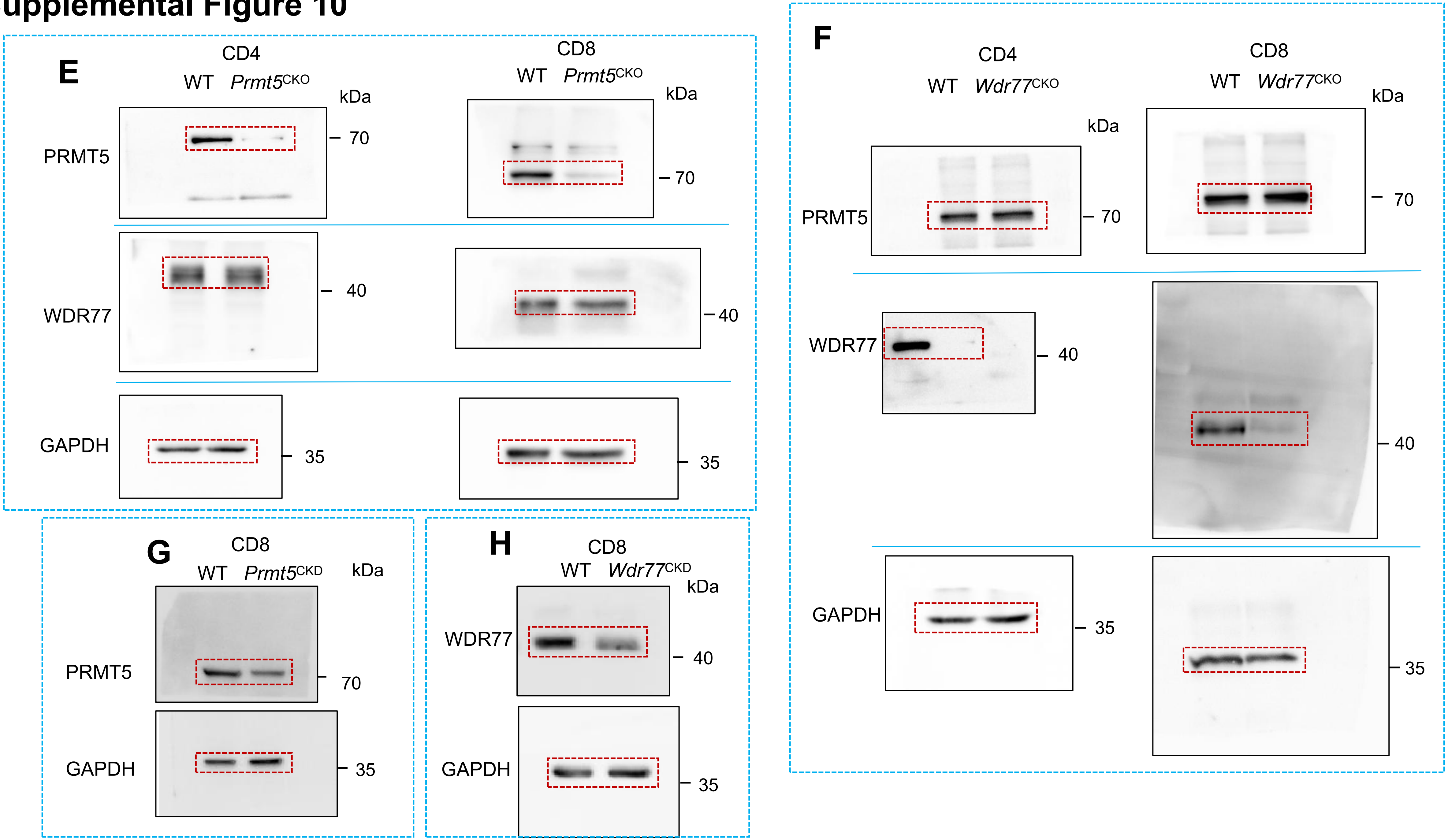

Supplemental Figure 16

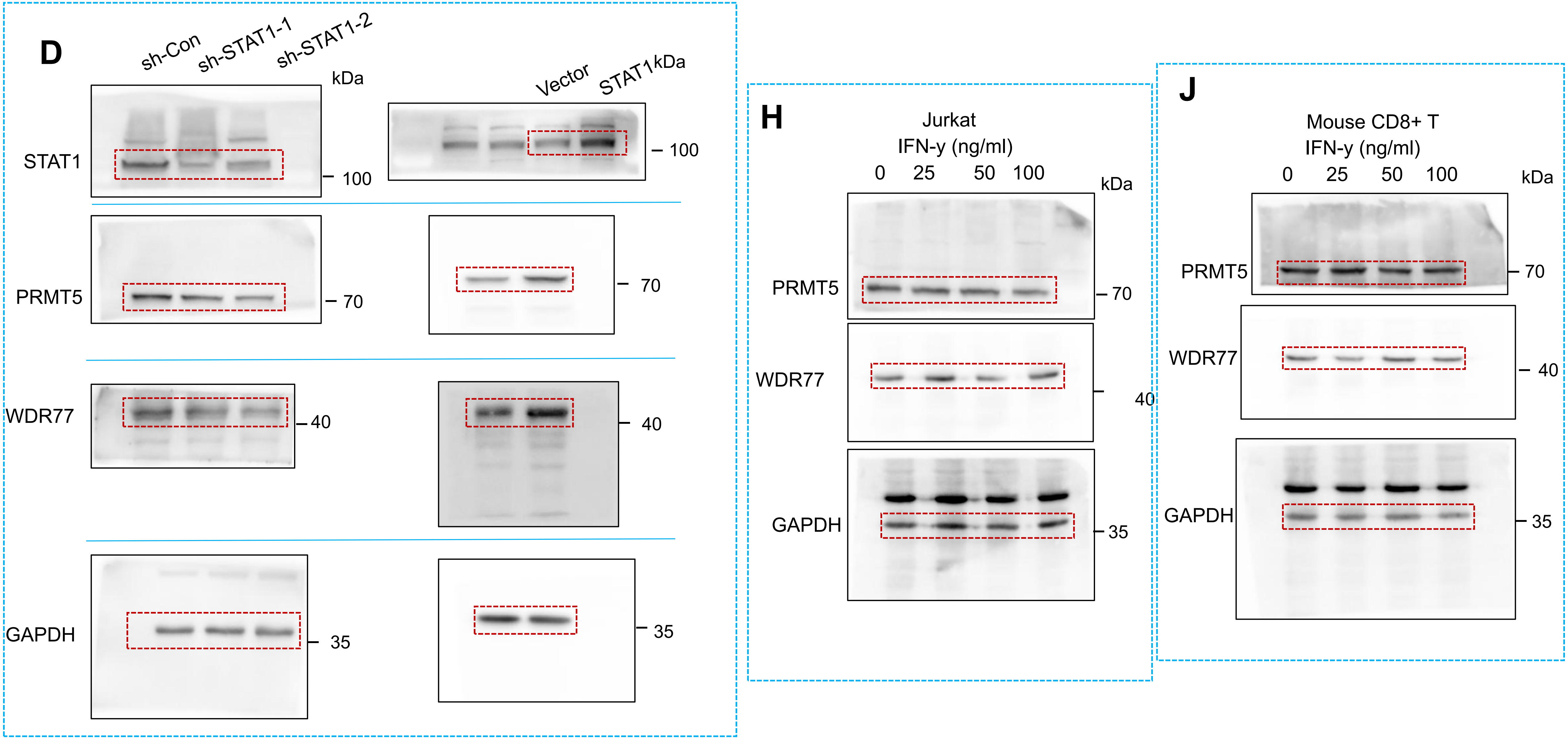

Supplemental Figure 16

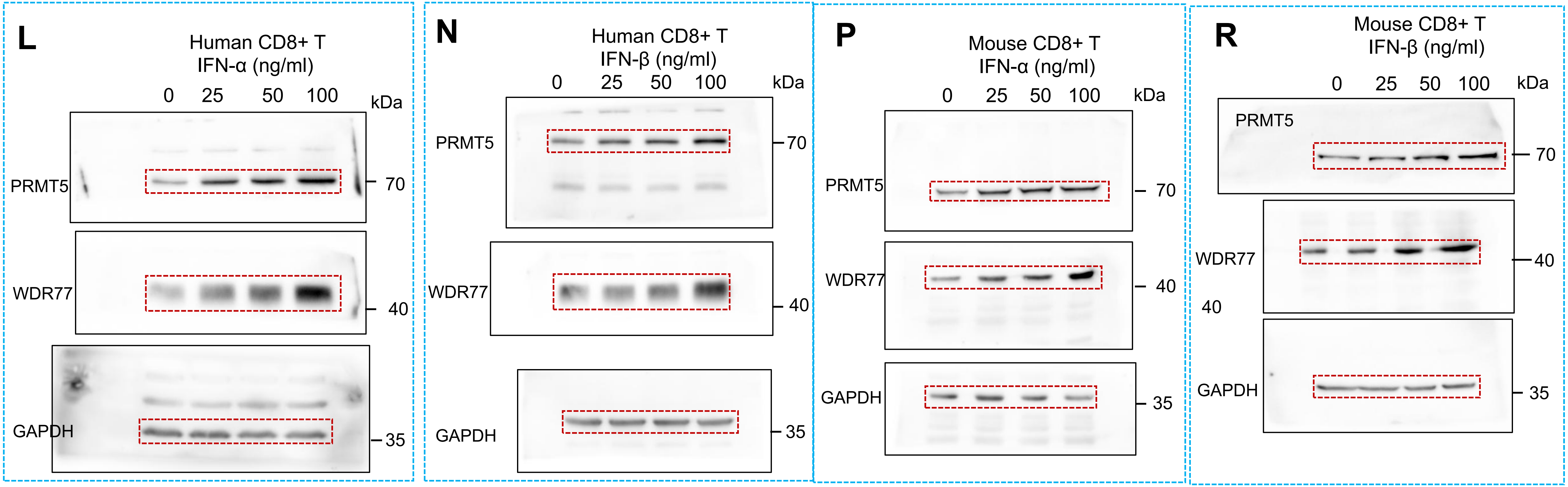

Supplemental Figure 17

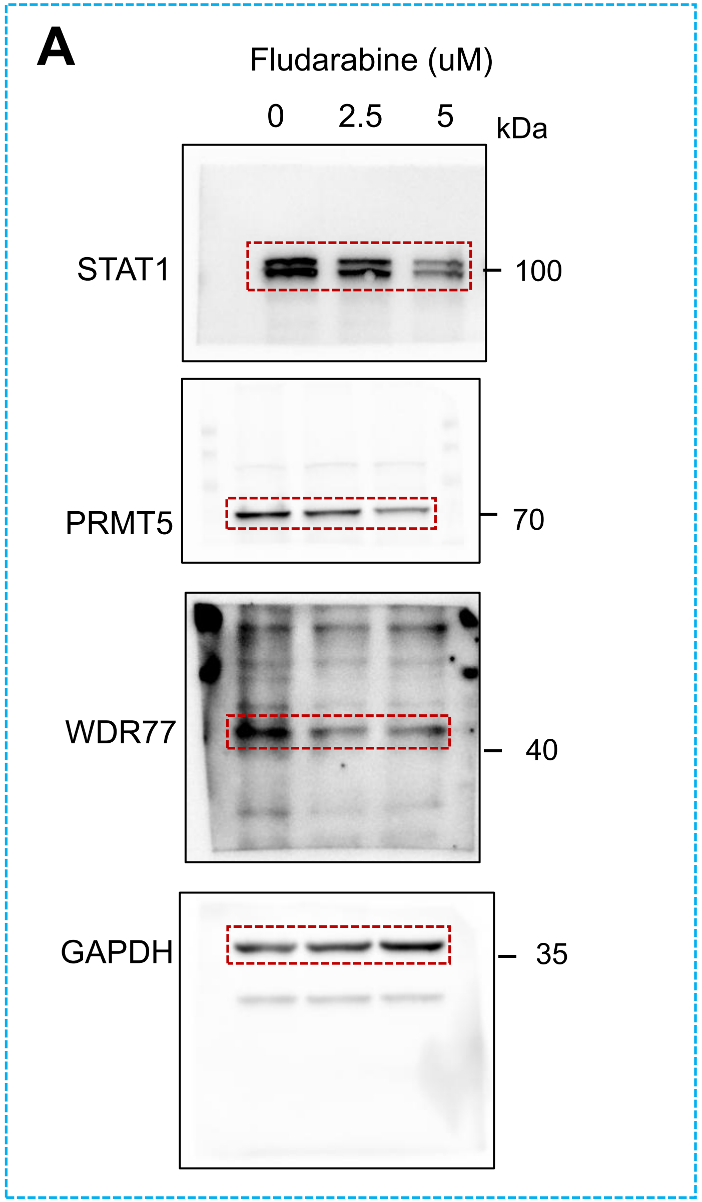

Supplement: Unedited blot and gel images [file jci-136-191469-s365.pdf]
